# Positive Effects of Prosocial Cartoon Viewing on Aggression Among Children: The Potential Mediating Role of Aggressive Motivation

**DOI:** 10.3389/fpsyg.2021.742568

**Published:** 2021-12-22

**Authors:** Qian Zhang

**Affiliations:** ^1^Center for Studies of Education and Psychology of Minorities in Southwest Area, Southwest University, Chongqing, China; ^2^Department of Early Childhood Education, Faculty of Education, Southwest University, Chongqing, China

**Keywords:** prosocial cartoon, aggression, aggressive motivation, children, hot sauce task

## Abstract

Prosocial cartoon is characterized by helping others solve difficulties, including helping, donating, sharing, comforting, and cooperating. The current study examined whether viewing a prosocial cartoon decreases aggression immediately upon exposure and the potential mediating role of aggressive motivation. Participants involve 168 children (*M*_age_ = 5.87 years, SD = 0.41) nominated by teachers as aggressive from three Chinese kindergartens. Children in the treatment group watched a prosocial cartoon (American cartoon “Handy Manny”), while children in the control group watched a nonprosocial cartoon (Chinese cartoon “Fruity Robo”). Afterward, the Hot Sauce Task (HST) was employed to assess aggressive behavior, and Aggressive Motivation Questionnaire (AMQ) was employed to assess aggressive motivation. Results revealed that viewing a prosocial cartoon (vs. a nonprosocial cartoon) did reduce children’s aggression immediately upon exposure. Specifically, males showed less aggressive behavior than females upon prosocial cartoon exposure, while males showed more aggressive behavior than females upon nonprosocial cartoon exposure. Mediational analysis suggested that the prosocial cartoon effect on aggression was partially mediated by aggressive motivation, especially for males. Consistent with general aggression model (GAM), findings of the study indicated that short-term exposure to a prosocial cartoon decreased children’s aggression by reducing aggressive motivation.

## Introduction

Previous researchers have investigated the negative effects of cartoon violence on aggression (e.g., Kirsh, 2006). However, experimental studies on the positive effects of prosocial cartoons on aggression are relatively sparse. The problem of children’s aggression may need to be addressed in depth by prosocial cartoon exposure. What is meant by prosocial cartoons? Based on previous literature that children’s prosocial behavior includes helping, donating, sharing, comforting, and cooperating (Drummond et al., 2017), we apply this definition to cartoon characters. The core of prosocial behavior is the intention to help others. Accordingly, prosocial cartoons refer to the types of cartoon video in which prosocial characters help others solve difficulties, including help, donation, sharing, comfort, and cooperation.

The definition of aggression is contrary to the definition of prosocial cartoons, which means that any behavior directed toward another individual that is carried out with the proximate intent to cause harm (Anderson and Bushman, 2002a). The concepts of aggression can be thoroughly defined or explained as developmentally relevant to young children. For example, the development and rapidly changing nature of aggression in early childhood (Crick et al., 2006; Perry et al., 2021); the shift from physical to more verbal and relational form (Grotpeter and Crick, 1996; Swit et al., 2018; Ostrov et al., 2019; Hart and Ostrov, 2020). In the present study, we use an “artificial measure of aggression” (spicy hot sauce given to a child) as the operational definition of aggressive behavior in laboratory settings for ethical reasons based on previous research (e.g., Lieberman et al., 1999). How is the spicy hot sauce an artificial measure of aggression? In the spicy hot sauce test/task, children can select a certain rank of hot sauce to another child who does not like spicy food. The rank of hot sauce (0–5 points) is indicative of aggressive behavior. Media is a significant inducement and a societal concern of children’s aggression (Bandura, 2001; Anderson and Bushman, 2002b). Noticeably, cartoon has become an attractive media for children to acquire social behavior, which may potentially affect children’s aggression. Given that aggressive and prosocial motivation are usually opposed to each other, we assume that aggressive motivation is an important mediator in the association between type of cartoon viewed and aggressive behavior, although the positive media research in this field is relative sparse compared to violent media.

### Theoretical Background

The general aggression model (GAM) of aggression, a further development of the general learning model (GLM), might be considered and a more explicit theory of aggressive cognitions or motivation developed to support this study. The GAM, is used as a theoretical framework to explain the mechanism underlying the media effects on aggressive behavior (Buckley and Anderson, 2006). The GAM details that the interaction between situational variables (violence and prosociality) and personal variables (e.g., gender and trait) activates the internal state (e.g., cognition and physiological arousal) to influence individual’s aggressive behavior. Although previous research into the effects of prosocial media indicate that prosocial media exposure decreases aggression (Greitemeyer, 2011; Greitemeyer et al., 2012; Whitaker and Bushman, 2012; Gentile et al., 2014; Prot et al., 2014; Teng, 2015), but why should prosocial media exposure reduce aggression? Based on this model, we attempt to investigate how viewing a prosocial cartoon (situational variable) and gender (personal variable) affect aggressive behavior through aggressive motivation after exposure.

### Cartoons and Aggression

Cartoon has become children’s favorite media program since the 1990s (Jiang, 2013). Many researchers assume that cartoon can affect children’s morality, socialization, and behavior (Ogle et al., 2016). Specifically, there is a positive link between aggressive cartoons and children’s aggression, but a negative link between aggressive cartoons and positive behavior (Sanson and Muccio, 1993; Sprafkin et al., 2010). Importantly, cartoon preference is a significant predictor of aggressive behaviors among 48- to 66-month-old children (Soydan et al., 2017). With the development of positive psychology, child psychologists began to pay attention to the positive effects of cartoon on children’s aggression (e.g., Zhang et al., 2020). Exposure to cartoons is an important way to promote children’s good behavior (e.g., healthy eating) and alleviate their problem behavior (Gonçalves et al., 2018).

### Gender and Aggression

Generally, for the most part the literature is clear regarding boys’ greater displays of physical aggression than girls in early childhood (Zhang et al., 2003; Ostrov and Keating, 2004; Alink et al., 2006; Baillargeon et al., 2007; Lussier et al., 2012; Björkqvist, 2018; Kung, 2018). Specifically, the studies that have looked specifically at cartoon viewing, aggression and gender have shown that boys exhibit more physical aggression than girls after watching violent television cartoons (Luther and Legg, 2010). Noticeably, boys show less aggressive behaviors than girls after prosocial media exposure (Li et al., 2016; Zhang et al., 2020). However, other researchers hold the opposite view, arguing that girls show less cognitive and affective aggression than boys when they hear songs with prosocial lyrics (Böhm et al., 2016). Coplaying video games between parents and children can reduce sibling conflict among females rather than males (Coyne et al., 2016). In addition, previous meta-analysis has shown that gender does not moderate any effects of media exposure (Anderson et al., 2010). Given boys typically and developmentally should be expected to have higher levels of aggression compared to girls in this age group. In view of this, it is reasonable to include gender in the experimental design.

### Age and Aggression

Age is negatively correlated with physical aggression, and children with younger age yield higher aggression than those with older age (Bukowski, 1990). Likewise, media has produced a greater effect on adults than children (Bushman and Huesmann, 2006). On the contrary, a handful of researchers did not find age differences in the media effects on aggression (Kirsh, 2006; Tear and Nielsen, 2014). As such, there is a dispute over age effects in aggression in the previous literature. In the present study, given such a limited time span between 5 and 6 year olds, it is unlikely age to have an impact. In other words, there is not a qualitative difference between 5-year-old vs. 6-year-old children in relation to aggression. Thus, we do not plan to include age in the experimental design.

### Aggressive Motivation and Aggressive Behavior

Motives (motivations) are the main indicators of subsequent aggression (Hellström et al., 2012). In particular, revenge aggressive motivation can mediate the violent video game effect on aggression among young women in the United States (Anderson and Murphy, 2003). More specifically, researchers have once used the Aggressive Motivation Questionnaire (AMQ) to measure children’s aggressive motivation (see Anderson et al., 2008; Anderson and Carnagey, 2009). Aggressive motivation includes instrumental and revenge motivations (Lindsay and Anderson, 2000). Specifically, the aggressive motivation is the sum-score of instrumental and revenge motivation (Anderson and Murphy, 2003). Meanwhile, exposure to prosocial media increases prosocial behavior through cognition and affect (Saleem et al., 2012), and the prosocial video game effect on aggressive behavior is mediated by reduced retaliation (Greitemeyer et al., 2012). Thus, aggressive motivation is a possible mediator of aggression.

### Developmental Challenges of Aggression During Early Childhood

In the present study, young children in early childhood were included because this is an age group that is largely understudied concerning the positive effects of prosocial television (Mares and Woodard, 2005). Mass media can help people prepare for the challenges they will encounter in life by modeling such situations and effective ways of overcoming them (Bandura, 2001, 2004). Considering the developmental challenges that children face during childhood (Eisenberg et al., 2007), prosocial cartoon may function as a prevention measure of aggression.

### The Present Study

To date, experimental studies have shown that exposure to cartoon violence causes children to behave aggressively afterwards (Kirsh, 2006). In particular, prosocial video games decrease children’s accessibility of aggressive cognition and aggressive behavior (Greitemeyer and Osswald, 2009; Greitemeyer, 2011; Greitemeyer et al., 2012). Notably, the main participants are adolescents instead of young children (e.g., Liang, 2015). To our understanding, it is not so much the effect of cartoon violence that is of concern, but rather whether prosocial cartoon can be used as an intervention measure to reduce children’s aggression. Thus, the first goal is to test whether prosocial cartoon viewing influences aggression in children with aggressive behavior by conducting an experiment. The second goal is to find out which group (i.e., gender) is most likely to be affected by this effect. The final goal is to test whether aggressive motivation mediates the prosocial cartoon effect. Based on the literature review, we hypothesize that as:

*Hypothesis 1: Viewing a prosocial cartoon will reduce aggressive behavior compared to viewing a nonprosocial cartoon.*

*Hypothesis 2: Boys in the prosocial condition will have higher levels of aggression than girls in this condition.*

*Hypothesis 3: Aggressive motivation will mediate the prosocial cartoon effect on aggressive behavior.*

## Materials and Methods

### Participants

In the fall semester of 2019, a total of 168 children (50% females, *M*_age_ = 5.87, SD = 0.41) were recruited from three kindergartens with branches in China. Given that previous research literature showed that teacher nominations can be a good alternative to peer nominations for social preference and popularity (cf. Van et al., 2015), we recruited aggressive children based on teacher nominations. Specifically, each teacher selected the top 10 aggressive children in their class who were most likely to show aggression toward other peers.

Also, we attempt to avoid that something other than the prosocial content accounts for the impact on aggression. For example, because the cartoons are fun to watch, they may have had beneficial effects on the children’s mood (which then accounted for the effect on aggression). Thus, half of the kindergartners viewing a prosocial cartoon were regarded as the treatment group, and the other half viewing a nonprosocial cartoon were regarded as the control group. Participants were randomly assigned to either the treatment or control condition (a randomized controlled trial). All participants have a normal vision or corrected visual acuity, without any mental disorders. This experiment has been reviewed by the institutional review board affiliated with Southwest University that approved human subject protections. No participants failed to complete the experiment.

### The Prosocial Cartoon

The theoretical basis of the cartoon selection and types of prosocial behaviors are selected according to the previous literature (Zhang et al., 2020). In the present study, we included a manipulation check procedure. Five cartoons were selected as the preliminary cartoon materials, including Doraemon, Fruity Robo, Smart Ikkyu San, Handy Manny, and Peppa Pig. The Japanese cartoon “Doraemon” tells a story about a robot cat from the 22nd century who is entrusted by his master Nobiyoshi. Back in the 20th century. The robot cat helps Nobiyoshi’s great-grandfather, Nobiyoshio, a primary school student, to solve all kinds of difficulties around him with the help of various future props from his four-dimensional pocket. The Chinese cartoon “Fruity Robo” tells a story that jelly people study and play happily in Jelly Martial Arts College. Unexpectedly, one day, an unexpected guest came, who was one of the four evil thieves. Because the evil thieves disturbed the peace in the college. In order to maintain peace, the abbot drove Yan Ye and ZiYi ShangGuan to Tianshan Mountain. No prosocial contents and scenes are included in this cartoon. The Japanese cartoon “Smart Ikkyu San” tells a story that the wise monk Ikkyu left home at a young age. He is usually diligent and smart, eager to help others, and likes to use his brain to solve problems. His intelligence is often admired by adults, and he can teach children a lot of common sense in daily life. The American cartoon “Handy Manny” tells a story that Oman and his tools rush to repair the school’s climbing frame to save the trapped children. British cartoon “Peppa Pig” (the episode fund-raising long-distance running) tells that the pig father to run through the long distance to Peppa’s kindergarten to earn money to repair the roof, and the other episode “middle pig” tells a story that Peppa Pig, brother George, Peppa mother, and Peppa father play ball-throwing game.

Based on the time duration of media exposure (Barlett et al., 2009), we limited the broadcasting time of each cartoon to 15 min. To do a manipulation check of cartoon materials, we invited 30 college students, 10 cartoon developers, 20 child psychology postgraduates, 20 postgraduates, 10 child parents, and 10 kindergarten teachers to rate the prosocial attributes of the five cartoons in terms of Interest (e.g., Is this cartoon interesting?), Difficulty (Is this cartoon difficult to watch?), Enjoyment (Is this cartoon enjoyful?), Prosocial Content (Does this cartoon have prosocial plot?), Prosocial Scene (Does this cartoon have prosocial images?), and Familiarity (Are you familiar with this cartoon?). The six dimensions anchored by a Likert five-point rating scale ranged from 1 point (very inconsistent) to 5 point (very consistent). One fifth of them (*n* = 20) watched one of five cartoons and made their assessments according to these six dimensions. The respondents evaluating the cartoons were voluntarily recruited. After their rating, they were given a nice gift for their supports. We delivered the cartoons to them *via* QQ software (a widely used chat APP in China), so they viewed the cartoons separately and made manipulation checks. One-way analysis of variance (ANOVA) was used to run data analysis to rate prosocial attributes of the five cartoons.

As shown in Table 1, there were significant differences in Prosocial Content [*F*(4, 95) = 19.04, *p* < 0.001, *d* = 0.88]. Handy Manny yielded significant highest Prosocial Content [*M* = 4.05, SD = 0.94] and Fruity Robo yielded lowest Prosocial Content [*M* = 1.75, SD = 0.64]. There were significant differences in Prosocial Scene [*F*(4, 95) = 34.06, *p* < 0.001, *d* = 1.18]. Handy Manny yielded significant highest Prosocial Scene [*M* = 4.15, SD = 0.59] and Fruity Robo yielded lowest Prosocial Scene [*M* = 1.65, SD = 0.59]. However, we did not find significant differences in Interest [*F*(4, 95) = 1.17, *p* = 0.33, *d* = 0.22], Difficulty [*F*(4, 95) = 0.24, *p* = 0.92, *d* = 0.10], Enjoyment [*F*(4, 95) = 1.55, *p* = 0.19, *d* = 0.25], and Familiarity [*F*(4, 95) = 1.22, *p* = 0.31, *d* = 0.22] among the five cartoons. Based on the rating standard of media violence mainly included violent scenes and contents (Anderson and Dill, 2000), we finally chose Handy Manny as a prosocial cartoon and Fruity Robo as a nonprosocial cartoon for follow-up experiment.

**Table 1:** Rating results of prosocial attributes of five cartoons (*N* = 100).

Dimension	Doraemon *M* ± SD	Fruity Robo *M* ± SD	Smart Ikkyu San *M* ± SD	Handy Manny *M* ± SD	Peppa pig *M* ± SD	*F*	*d*
Interest	3.95 ± 0.94	4.05 ± 1.10	4.30 ± 0.57	4.30 ± 0.66	4.40 ± 0.50	1.17	0.26
Difficulty	2.15 ± 1.09	2.00 ± 0.73	2.10 ± 0.91	1.90 ± 0.85	2.00 ± 0.86	0.24	0.27
Enjoyment	3.50 ± 1.19	3.85 ± 0.99	3.15 ± 0.93	3.75 ± 0.91	3.35 ± 1.09	1.55	1.35
Content	3.25 ± 0.85	1.75 ± 0.64	3.15 ± 1.04	4.05 ± 0.94	3.65 ± 0.93	19.04^***^	1.39
Scene	3.40 ± 0.75	1.65 ± 0.59	3.35 ± 0.88	4.15 ± 0.59	3.80 ± 0.83	34.06^**^	0.13
Familiarity	3.20 ± 1.20	3.30 ± 1.26	3.10 ± 0.85	3.55 ± 1.19	3.75 ± 0.79	1.22	0.13

** *p* < 0.01 and ***p < 0.001.

### Measures

#### Aggressive Behavior

The hot sauce paradigm (HSP), an artificial/proxy measure of aggression, was used to assess aggressive behavior due to ethical reasons. After viewing cartoons, the children were asked to feed hot sauce to another child in the picture immediately. The cover story was that participants were told that the child in a picture had many food preferences, but he was most afraid of eating hot sauce and other spicy food. Lab assistants told the participants that they must choose one rank/level of hot sauce powder for the child to eat. We also told each participants “If you do not set one level of hot sauce powder for this child, or he will set one level of hot sauce powder for you!” Participants can choose one of six ranks/levels of hot sauce powder (0 point = no sauce powder at all and 5 point = hottest sauce powder) for the child in the picture. Thus, the levels of hot sauce powder chosen by the participants for a child in the picture represents a measure of aggressive behavior. Zero point represents no aggressive behavior, and five point represents highest aggressive behavior. Existing research has demonstrated that the HSP is positively linked to trait aggression scores measured by Buss-Perry Aggression Questionnaire (Buss and Perry, 1992) and has good reliability and validity (Lieberman et al., 1999; Sun and Liu, 2019). Notably, children in the study were selected based on teacher nomination of aggressive children. It should be noted that 5-point scale is used for the rating of aggression. We only considered the aggressive behavior tendency and teacher did not have bias in behavioral nominations.

#### Aggressive Motivation

Aggressive Motivation Questionnaire, including instrumental aggressive motivation and revenge aggressive motivation, is used to measure aggressive motivation. The experimenters asked participants why they set the particular level of hot sauce powder for the child in the picture who did not like eating pepper or spicy food. Participants should answer six questions/items to reflect how motivation function when deciding on the setting level of hot sauce powder. Responses were given on a 5-point scale anchored at 1 (not consistent at all), 2 (a little bit consistent), 3 (somewhat consistent), 4 (consistent), and 5 (a lot consistent). The items of instrumental motivation are as: (1) “*I want to impair the child in the picture”*; (2) *“I want to make the child in the picture like eating pepper.”* The items of revenge motivation are as: *(1) “I want to make this child feel uncomfortable”*; (2) *“I want to hurt this child”*; (3) *“I want to attack the child with the same level of hot sauce powder”*; and (4) *“I want to select higher hot sauce powder than the child does for me.”* The experimenters evaluate the consistency of the motivation. In the present study, the internal consistency reliability coefficient of AMQ completed by participants is 0.96.

### Procedure

In a cover story, participants were asked to watch cartoons for 15 min in the quiet hall of the kindergartens. Afterward, the AMQ captured children’s aggressive motivation was filled out by the children individually. All participants and their parents gave written informed consent for participation and acknowledged that their data would be fully anonymized. The experiment was approved by the researchers’ university Ethics Committee in accordance with the Declaration of Helsinki. Their consent rates reached 100%. Then, all participants completed the HSP to measure the baseline levels of aggressive behavior (pretest). In the experimental condition, 84 children were randomly assigned to view Handy Manny. In the control condition, 84 children were randomly assigned to view Fruity Robo. Both clips were both 15 min in length. When the cartoon clip ended, the children completed a hot sauce task (HST). Participants completed the experiment in groups of six, 28 groups in total. After the HST was finished, the children had to fill out the AMQ individually by an interview. In line with the cover story, the questionnaire contained questions about the cartoon characters. Upon completing the questionnaire, children received a sheet of nice stickers and were accompanied back to the classroom.

### Experimental Design

A 2 (cartoon: prosocial vs. nonprosocial) × 2 (gender: male vs. female) experimental-control mixed design was employed. The independent variables are cartoon and gender. The main outcome variable is aggressive behavior (setting level of hot sauce powder).

## Results

### Pretest Levels of Aggression

We used an independent sample *t*-test to compare differences in baseline aggressive behavior between the treatment and the control group. Overall, there were no significant differences in aggressive behavior between the two groups [*t*(166) = 0.45, *p* = 0.65, *d* = 0.07; *η_p_^2^* = 0.001]. Finally, there were no significant gender differences in aggressive behavior at baseline [*t*(166) = 0.27, *p* = 0.79, *d* = 0.04; *η_p_^2^* < 0.001]. Thus, the pretest levels of aggression between the treatment and the control group were homogeneous. Although this may at first appear surprising, recall that we selected more aggressive children for this sample.

### Descriptive Statistics of Post-tested Aggressive Motivation and Behavior

Tables 2 and 3 show the mean and standard deviation of aggressive motivation and aggressive behavior under four conditions. Overall, children in a prosocial cartoon condition demonstrate less aggressive motivation and aggressive behavior than those in a nonprosocial cartoon condition. In addition, males show less aggressive motivation and aggressive behavior (hot sauce powder level) than females only in a prosocial cartoon condition (experimental condition), whereas males show more aggressive behavior than females in a nonprosocial cartoon condition (control condition). Based on these preliminary analyses, we further conducted specific analyses of these main variables.

**Table 2:** Means and standard deviation of post-tested aggressive motivation.

Gender	Prosocial *M* ± SD	*N*	Nonprosocial *M* ± SD	*N*
Male	1.79 ± 1.18	42	3.17 ± 1.38	42
Female	2.21 ± 1.20	42	3.31 ± 1.49	42
Total	2.00 ± 1.20	84	3.24 ± 1.43	84

**Table 3:** Means and standard deviation of post-tested aggressive behavior.

Gender	Prosocial *M* ± SD	*N*	Nonprosocial *M* ± SD	*N*
Male	2.17 ± 0.99	42	3.52 ± 1.06	42
Female	2.64 ± 0.91	42	3.02 ± 1.00	42
Total	2.40 ± 0.97	84	3.27 ± 1.06	84

### Prosocial Cartoon Effect on Aggressive Motivation

A 2 (Cartoon: prosocial vs. nonprosocial) × 2 (Gender: male vs. female) analysis of covariance (ANCOVA) was performed to test the main effect and interaction on aggressive motivation, with age included as a covariate (continuous variable). The main effect of cartoon on aggressive motivation was significant. Children in the prosocial cartoon condition showed lower aggressive motivation than those in the nonprosocial cartoon condition [*F*(1, 163) = 37.11, *p < 0*.001, *d* = 0.95, *η_p_^2^* = 0.18; *M* = 2.00 (SE = 0.14) < *M* = 3.24 (SE = 0.14)]. However, the main effects of gender [*F*(1, 163) = 1.89, *p =* 0.17, *d* = 0.21, *η_p_^2^* = 0.01] and age [*F*(1, 163) = 0.41, *p =* 0.52, *d* = 0.10, *η_p_^2^* = 0.002] on aggressive motivation were not significant. Similarly, the cartoon x gender interaction on aggressive motivation was not significant [*F*(1, 163) = 0.54, *p =* 0.46, *d* = 0.11, *η_p_^2^* = 0.003].

### Prosocial Cartoon Effect on Aggressive Behavior

**Figure 1:**
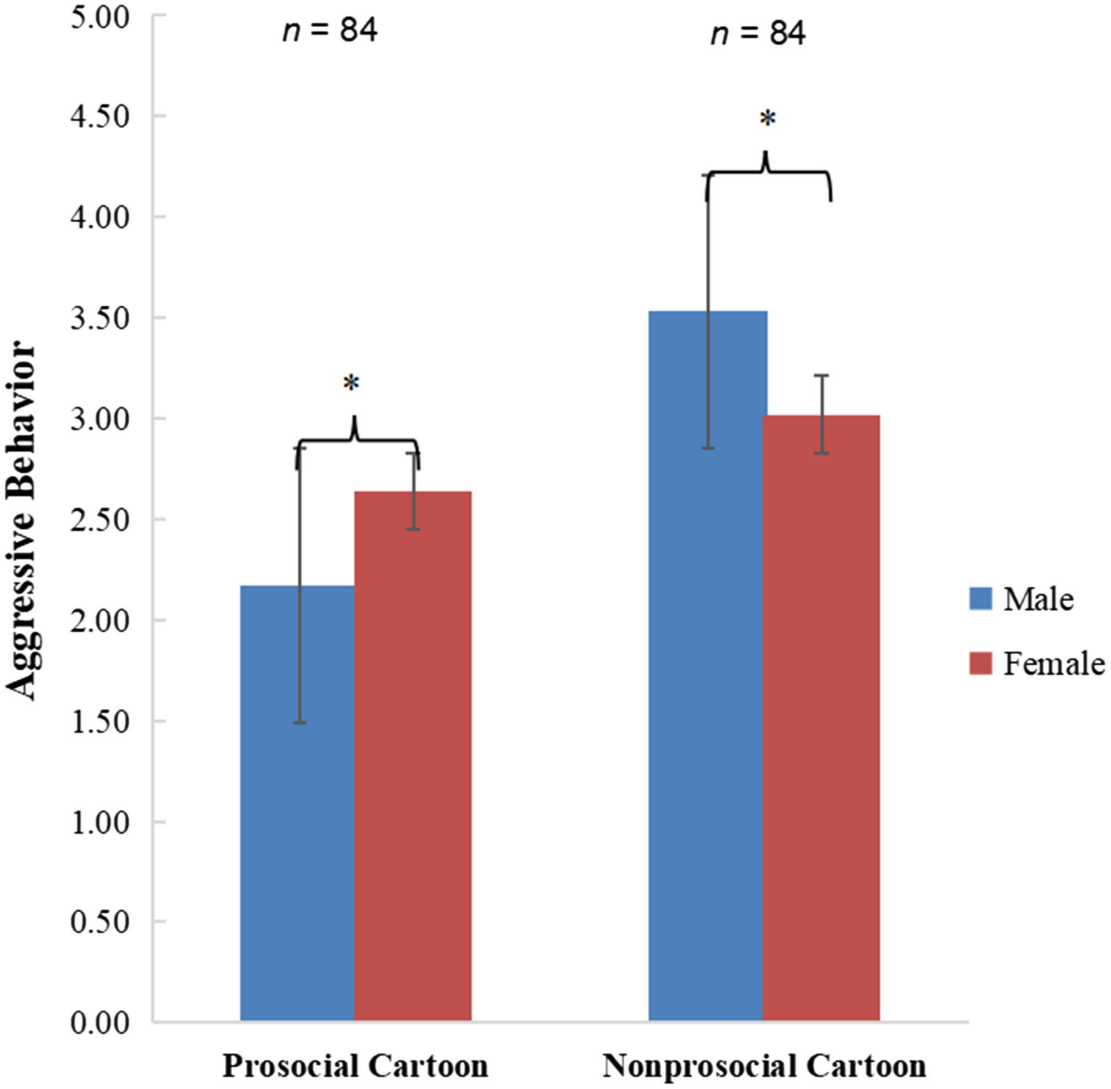
Cartoon × gender interaction on aggressive behavior. ^*^p < 0.05.

To test Hypotheses 1 and 2, we conducted a 2 (Cartoon: prosocial vs. nonprosocial) × 2 (Gender: male vs. female) ANCOVA on aggressive behavior, with age controlled as a covariate. The main effect of cartoon on aggressive behavior was significant. Children in a prosocial cartoon condition displayed less aggressive behavior than those in a nonprosocial cartoon condition [*F*(1, 163) = 32.24, *p <* 0.001, *d* = 0.88, *η_p_^2^* = 0.16; *M* = 2.40 (SE = 0.11) < *M* = 3.28 (SE = 0.11)]. However, the main effects of gender [*F*(1, 163) = 0.01, *p =* 0.93, *d* = 0.02, *η_p_^2^* < 0.001] and age [*F*(1, 163) = 0.13, *p =* 0.72, *d* = 0.06, part. *η*^2^ < 0.001] on aggressive behavior were not significant. In addition, the cartoon x gender interaction on aggressive behavior was significant [*F*(1, 163) = 10.24, *p =* 0.002, *d* = 0.50, *η_p_^2^* = 0.06; Figure 1]. A simple effect analysis indicated that males displayed less aggressive behavior than females in a prosocial cartoon condition [*F*(1, 163) = 4.84, *p =* 0.03, *d* = 0.34, *η_p_^2^* = 0.03; *M* = 2.17 (SE = 0.15) < *M* = 2.64 (SE = 0.15)], while males displayed more aggressive behavior than females in a nonprosocial cartoon condition [*F*(1, 163) = 5.41, *p =* 0.02, *d* = 0.36, *η_p_^2^* = 0.03; *M* = 3.53 (SE = 0.15) > *M* = 3.02 (SE = 0.15)].

### Aggressive Motivation as a Mediator of Aggressive Behavior

Since watching a prosocial cartoon could decrease aggressive motivation and aggressive behavior, we further tested Hypothesis 3 that aggressive motivation would mediate the prosocial cartoon effect on aggressive behavior. Meanwhile, given that the correlation between aggressive motivation and aggressive behavior was significant [*r* = 0.34, *p* < 0.001], aggressive motivation was a potential mediator in the relationship between prosocial cartoon and aggressive behavior.

**Figure 2:**
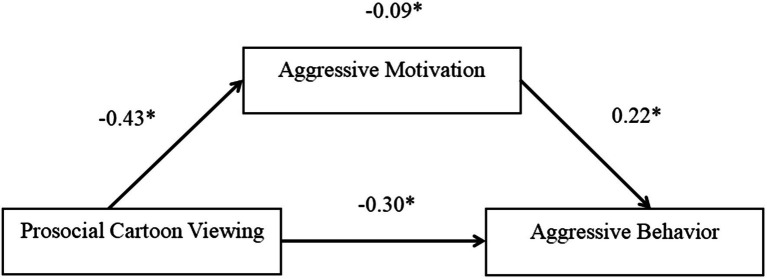
Partial mediation model of prosocial cartoon viewing on aggressive behavior through aggressive motivation. 1 = prosocial and 0 = nonprosocial; standardized path coefficients; and solid lines represent significant paths. ^*^p < 0.05.

We ran regression analysis by using the PROCESS 3.0 macro Model 4 of SPSS with all data standardized (Hayes, 2013). In this model, prosocial cartoon viewing was the predictor, aggressive motivation was a mediator, aggressive behavior was the outcome variable. Age and gender were controlled as covariates. The direct effect of prosocial cartoon viewing on aggressive behavior was significant [*β* = −0.31; SE = 0.08; 95% CI = (−0.46, −0.15)]. Prosocial cartoon viewing significantly predicted less aggressive motivation [*β* = −0.43; SE = 0.07; 95% CI = (−0.57, −0.29)]. Less aggressive motivation significantly predicted less aggressive behavior [*β* = 0.22; SE = 0.08; 95% CI = (0.06, 0.37)], and the mediated path from prosocial cartoon viewing through aggressive motivation to aggressive behavior was significant [*β* = −0.09; SE = 0.04; 95% CI = (−0.17, −0.02); Figure 2].

**Figure 3:**
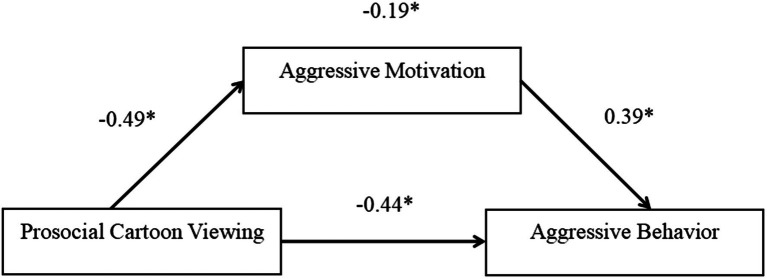
Partial mediation model of prosocial cartoon viewing on aggressive behavior through aggressive motivation for males. 1 = prosocial and 0 = nonprosocial; standardized path coefficients; and solid lines represent significant paths. ^*^p < 0.05.

In particular, we tested males and females in a separate mediation model (while controlling for age as a covariate). The direct effect of prosocial cartoon viewing on aggressive behavior was significant for males [*β* = −0.44; SE = 0.11; 95% CI = (−0.65, −0.22)], but not significant for females [*β* = −0.16; SE = 0.10; 95% CI = (−0.37, 0.05)]. Prosocial cartoon viewing significantly predicted aggressive motivation for males [*β* = −0.49; SE = 0.10; 95% CI = (−0.67, −0.28)] and for females [*β* = −0.38; SE = 0.10; 95% CI = (−0.58, −0.17)]. Aggressive motivation significantly predicted aggressive behavior for males [*β* = 0.39; SE = 0.11; 95% CI = (0.17, 0.61)], but not significant for females [*β* = 0.03; SE = 0.10; 95% CI = (−0.17, 0.24)]. The mediated path from prosocial cartoon viewing through aggressive motivation to aggressive behavior was significant for males [*β* = −0.19; SE = 0.07; 95% CI = (−0.35, −0.07); Figure 3], but not significant for females [*β* = −0.01; SE = 0.04; 95% CI = (−0.10, 0.08)].

## Discussion

### Interpretations of the Findings

The findings of our experiment contribute to the relatively small number of studies on the positive effects of prosocial media on children (Mares and Woodard, 2005; Christakis et al., 2013) by adding that exposure to a prosocial cartoon can decrease children’s aggression. The present findings imply that children exposed to a cartoon with prosocial behavior display less aggressive behavior than those exposed to a cartoon without prosocial behavior, at least in the short term. The dominant point is that pretest levels of aggression are changed for those who watch the prosocial cartoon, compared to what the levels are before. Thus, we can conclude that watching a prosocial cartoon leads to lower levels of aggression in comparison with watching a nonprosocial cartoon.

Consistent with Hypothesis 1, our results found that watching a prosocial cartoon reduces aggressive behavior in comparison with watching a nonprosocial cartoon based on non-significant differences in pretest levels of aggressive behavior between the experimental and control groups. The finding fits with the GLM that prosocial media exposure can inhibit aggression (Buckley and Anderson, 2006; Gentile et al., 2014) and replicates most literature that prosocial media exposure decreases aggression and increases prosocial behavior (Greitemeyer, 2011; Greitemeyer et al., 2012; Whitaker and Bushman, 2012; Prot et al., 2014; Leeuw et al., 2015; Teng, 2015). Why does prosocial cartoon exposure affect aggressive behavior? One possible explanation is that children may imitate prosocial behavior from cartoon characters and thus attenuate aggression according to social cognitive theory of mass communication (Bandura et al., 1961; Bandura, 2001). The second possible explanation is that the prosocial contents of media reduce aggression by enhancing the judgment and cognition of prosocial behavior (Teng, 2015). The third possible explanation is that the children’s TV programs are filled with more prosocial role models than adult TV programs (Smith et al., 2006; Padilla-Walker et al., 2013). For example, the prosocial behavior of Disney children’s animated films is very common among peers, families, and strangers (Padilla-Walker et al., 2013). Cartoon developers, teachers, and parents should utilize prosocial scripts in media for children’s social behavior (Thakkar et al., 2006; Kirkorian et al., 2008). Given the positive effects of prosocial cartoon, educators can make full use of prosocial cartoons as measures in the intervention and reduction of aggressive behavior among Chinese children.

Consistent with Hypothesis 2, our results found that males display less aggressive behavior than females in a prosocial cartoon condition, while males display more aggressive behavior than females in a nonprosocial cartoon condition. The result is in line with the finding that males are more affected by prosocial TV program or video games than females (Gentile et al., 2014; Shuai, 2015). Why are males more likely to be affected by prosocial cartoon than females? The explanation is that males have a bad inhibitory control ability than females in preschool children with aggressive behavior (Raaijmakers et al., 2008). Therefore, males should be regarded as a key group of aggression prevention and intervention. Educators can make males with aggressive behavior to watch prosocial cartoons to reduce their potential aggression.

Consistent with Hypothesis 3, our results found that the prosocial cartoon effect on aggression is partially mediated by aggressive motivation, especially for males. Similarly, brief exposure to prosocial video games can increase prosocial thoughts and thus reduce aggressive behavior (Greitemeyer and Osswald, 2010; Zhang et al., 2016). Like the opposite research that violent video games increase aggressive behavior through motivation and intention (e.g., Anderson and Murphy, 2003; Greitemeyer and Mugge, 2014; Adachi and Willoughby, 2016; McGloin et al., 2016), prosocial cartoon attenuates aggressive behavior through reduced aggressive motivation. Prosocial cartoon viewing negatively predicted aggressive motivation, which, in turn, led to decreased aggression. Therefore, educators can decrease children’s aggressive behavior through lessening aggressive motivation (e.g., motivation training), especially for males.

### Limitations and Future Directions

Overall, there has been a lot of research into the effects of violent media on aggression. In contrast, much less experimental research has been devoted on positive effects of prosocial media on children’s social behavior. A strength of the study is evident in the attempt to assess the hypotheses with very young children. The current experiment examines the effects of watching a prosocial cartoon on aggression with the sample drawn from China. Hence, this is an interesting study that could be of wide interest. Our study extends the earlier work (Bushman and Huesmann, 2006) by examining the positive short-term media effects with Chinese aggressive children on the short-term. To our knowledge, not only has prior research concerned merely on the negative content of cartoons, the number of empirical studies on the favorable effects of cartoons on children is also scarce. Findings of this study demonstrate that engagement with prosocial cartoons predicts less aggressive motivation and behavior, especially for males with aggressive behavior. First, the random sample experimental-control mixed design, interesting age, gender effect, and randomization of participants ensure more balanced levels of aggression across groups as strong points. Second, not only are we examining the impact of prosocial cartoons on child aggression but also using a cross-national sample. The origin of the sample is a uniqueness and a strength of the study. It also has important cross-cultural implications, especially since we are using a variety of cartoons from different nations. Third, we can conclude that watching a prosocial cartoon led to lower levels of aggression than watching a nonprosocial cartoon. The dominant finding is that levels of aggression were changed for children who watched the prosocial cartoon, compared to what the levels were before based on their pretest levels of aggression. A final strength is that our finding supports GLM that prosocial cartoon viewing reduces aggressive behavior through aggressive motivation. This finding provides us with a new insight into children’s aggression intervention by increasing prosocial components in cartoons and decreasing aggressive motivation.

However, this experiment has several limitations: First, the study only tested the short-term prosocial cartoon effects, and longitudinal evidences with similar independent and outcome variables may be obtained in future research. Second, the homogeneous age (5–6 years) may limit the generalizability of these findings. Future research may consider expand the age range to provide more robust evidences for other age groups of children. Third, pre-screening levels of children’s aggression could have been considered and a more balanced random assignment to groups design considered. The fact that boys showed lower levels of aggression in the prosocial group is not a natural reflection of boys expected level of aggression (especially physical aggression) in this age group in comparison with girls; therefore, the lower levels of aggression in boys in this group may suggest they are atypical and in fact, Figure 1 shows they have the lowest levels of aggression among all the study children and therefore may be more likely to respond well to the cartoon intervention, especially given their aggressive motivation also appear to be low as assessed in the motivation scale etc. This is somewhat explained in the finding that aggressive motivation mediated the association between prosocial cartoon viewing and aggression for these lower aggression males only. As such given that males and females in the prosocial viewing condition had lower levels of aggression compared to the control condition, this is a suggestion that the intervention only worked for the lowest aggressive children in the group and may be more likely to work for children with low aggressive motivation, who also happened to be males. Future research should complete new analyses and new hypotheses developed with a more balanced sample design. Finally, the study used a proxy measure of aggression (HSP) in a laboratory setting, which may be criticized because this measure may lack ecological validity. We should note that there are differences between realistic measures of aggression in field experiments and laboratory measures of aggression.

The question that arises whether prosocial cartoon viewing definitely reduces aggression in the long run for children who are nominated by teachers as aggressive. Based on the current knowledge, it is difficult to provide a straightforward answer to this. For instance, empathy and self-regulation can affect children’s prosocial behaviors toward strangers, friends, and family after prosocial animated movie exposure (Padilla-Walker and Christensen, 2011; Padilla-Walker et al., 2013). Altogether, more experimental studies will be warranted to provide a definite answer to the question whether viewing prosocial cartoons can decrease children’s aggression in the long term.

## Data Availability Statement

The original data-sets presented in the study are included in the article/Supplementary Material, further inquiries can be directed to the corresponding author at reasonable request.

## Ethics Statement

The studies involving human participants were reviewed and approved by Southwest University. Written informed consent to participate in this study was provided by the participants’ legal guardian/next of kin. Written informed consent was obtained from the minor(s)’ legal guardian/next of kin for the publication of any potentially identifiable images or data included in this article.

## Author Contributions

QZ designed this experimental study, collected the data, and made analyses.

## Funding

The study was supported by the Chongqing Talent Plan Project (2021YC043), the 111 program (B21036), and the Central University’s Fundamental Grant (SWU2009201).

## Conflict of Interest

The author declares that the research was conducted in the absence of any commercial or financial relationships that could be construed as a potential conflict of interest.

## Publisher’s Note

All claims expressed in this article are solely those of the authors and do not necessarily represent those of their affiliated organizations, or those of the publisher, the editors and the reviewers. Any product that may be evaluated in this article, or claim that may be made by its manufacturer, is not guaranteed or endorsed by the publisher.
