# Supplementary material for: Positive Effects of Prosocial Cartoon Viewing on Aggression Among Children: The Potential Mediating Role of Aggressive Motivation
Source: Front Psychol. 2021 Dec 22;12:742568. doi: 10.3389/fpsyg.2021.742568 (PMC8782158; doi:10.3389/fpsyg.2021.742568)
Supplement: Supplementary file 2 [file Data_Sheet_2.doc]

GLM motivation 后AB BY gender cartoon WITH age
  /METHOD=SSTYPE(3)
  /INTERCEPT=INCLUDE
  /EMMEANS=TABLES(OVERALL) WITH(age=MEAN)
  /EMMEANS=TABLES(gender) WITH(age=MEAN)
  /EMMEANS=TABLES(cartoon) WITH(age=MEAN)
  /EMMEANS=TABLES(gender*cartoon) WITH(age=MEAN)
  /PRINT=DESCRIPTIVE
  /CRITERIA=ALPHA(.05)
  /DESIGN=age gender cartoon gender*cartoon.


一般线性模型


附注	
创建的输出	25-SEP-2020 12:51:33	
注释		
输入	数据	F:\准备投稿的文章\目前投稿的文章\亲社会动画与攻击行为：动机中介-辣酱范式168\投搞材料\数据\实验数据\总数据.sav	
	活动的数据集	数据集1	
	过滤器	<none>	
	权重	<none>	
	拆分文件	<none>	
	工作数据文件中的 N 行	181	
缺失值处理	对缺失的定义	用户定义的缺失值作为缺失数据对待。	
	使用的案例	统计量的计算将基于带有有效数据的所有案例，而这些有效数据适用于模型中的所有变量。	
语法	GLM motivation 后AB BY gender cartoon WITH age
  /METHOD=SSTYPE(3)
  /INTERCEPT=INCLUDE
  /EMMEANS=TABLES(OVERALL) WITH(age=MEAN)
  /EMMEANS=TABLES(gender) WITH(age=MEAN)
  /EMMEANS=TABLES(cartoon) WITH(age=MEAN)
  /EMMEANS=TABLES(gender*cartoon) WITH(age=MEAN)
  /PRINT=DESCRIPTIVE
  /CRITERIA=ALPHA(.05)
  /DESIGN=age gender cartoon gender*cartoon.	
资源	处理器时间	00:00:00.03	
	已用时间	00:00:00.03	


[数据集1] F:\准备投稿的文章\目前投稿的文章\亲社会动画与攻击行为：动机中介-辣酱范式168\投搞材料\数据\实验数据\总数据.sav


主体间因子	
	值标签	N	
gender	1	male	84	
	2	female	84	
cartoon	0	nonprosocial	84	
	1	prosocial	84	


描述性统计量	
	gender	cartoon	均值	标准 偏差	N	
motivation	male	nonprosocial	3.1667	1.37781	42	
		prosocial	1.7857	1.17982	42	
		总计	2.4762	1.45185	84	
	female	nonprosocial	3.3095	1.48961	42	
		prosocial	2.2143	1.20032	42	
		总计	2.7619	1.45303	84	
	总计	nonprosocial	3.2381	1.42794	84	
		prosocial	2.0000	1.20241	84	
		总计	2.6190	1.45516	168	
后AB	male	nonprosocial	3.5238	1.06469	42	
		prosocial	2.1667	.98567	42	
		总计	2.8452	1.22714	84	
	female	nonprosocial	3.0238	.99971	42	
		prosocial	2.6429	.90585	42	
		总计	2.8333	.96734	84	
	总计	nonprosocial	3.2738	1.05683	84	
		prosocial	2.4048	.97089	84	
		总计	2.8393	1.10161	168	


多变量检验a	
效应	值	F	假设 df	误差 df	Sig.	
截距	Pillai 的跟踪	.070	6.091b	2.000	162.000	.003	
	Wilks 的 Lambda	.930	6.091b	2.000	162.000	.003	
	Hotelling 的跟踪	.075	6.091b	2.000	162.000	.003	
	Roy 的最大根	.075	6.091b	2.000	162.000	.003	
age	Pillai 的跟踪	.003	.231b	2.000	162.000	.794	
	Wilks 的 Lambda	.997	.231b	2.000	162.000	.794	
	Hotelling 的跟踪	.003	.231b	2.000	162.000	.794	
	Roy 的最大根	.003	.231b	2.000	162.000	.794	
gender	Pillai 的跟踪	.012	1.009b	2.000	162.000	.367	
	Wilks 的 Lambda	.988	1.009b	2.000	162.000	.367	
	Hotelling 的跟踪	.012	1.009b	2.000	162.000	.367	
	Roy 的最大根	.012	1.009b	2.000	162.000	.367	
cartoon	Pillai 的跟踪	.261	28.662b	2.000	162.000	.000	
	Wilks 的 Lambda	.739	28.662b	2.000	162.000	.000	
	Hotelling 的跟踪	.354	28.662b	2.000	162.000	.000	
	Roy 的最大根	.354	28.662b	2.000	162.000	.000	
gender * cartoon	Pillai 的跟踪	.059	5.091b	2.000	162.000	.007	
	Wilks 的 Lambda	.941	5.091b	2.000	162.000	.007	
	Hotelling 的跟踪	.063	5.091b	2.000	162.000	.007	
	Roy 的最大根	.063	5.091b	2.000	162.000	.007	

a. 设计 : 截距 + age + gender + cartoon + gender * cartoon	
b. 精确统计量	


主体间效应的检验	
源	因变量	III 型平方和	df	均方	F	Sig.	
校正模型	motivation	69.378a	4	17.345	9.946	.000	
	后AB	41.863b	4	10.466	10.609	.000	
截距	motivation	10.353	1	10.353	5.937	.016	
	后AB	8.622	1	8.622	8.740	.004	
age	motivation	.712	1	.712	.408	.524	
	后AB	.130	1	.130	.132	.717	
gender	motivation	3.287	1	3.287	1.885	.172	
	后AB	.009	1	.009	.009	.926	
cartoon	motivation	64.707	1	64.707	37.107	.000	
	后AB	31.809	1	31.809	32.244	.000	
gender * cartoon	motivation	.940	1	.940	.539	.464	
	后AB	10.099	1	10.099	10.238	.002	
误差	motivation	284.241	163	1.744			
	后AB	160.798	163	.986			
总计	motivation	1506.000	168				
	后AB	1557.000	168				
校正的总计	motivation	353.619	167				
	后AB	202.661	167				

a. R 方 = .196（调整 R 方 = .176）	
b. R 方 = .207（调整 R 方 = .187）	


估算边际均值


1. 总均值	
因变量	均值	标准 误差	95% 置信区间	
			下限	上限	
motivation	2.619a	.102	2.418	2.820	
后AB	2.839a	.077	2.688	2.991	

a. 模型中出现的协变量在下列值处进行评估: age = 5.8723.	


2. gender	
因变量	gender	均值	标准 误差	95% 置信区间	
				下限	上限	
motivation	male	2.479a	.144	2.194	2.764	
	female	2.759a	.144	2.474	3.044	
后AB	male	2.846a	.108	2.632	3.061	
	female	2.832a	.108	2.618	3.046	

a. 模型中出现的协变量在下列值处进行评估: age = 5.8723.	


3. cartoon	
因变量	cartoon	均值	标准 误差	95% 置信区间	
				下限	上限	
motivation	nonprosocial	3.240a	.144	2.955	3.524	
	prosocial	1.998a	.144	1.714	2.283	
后AB	nonprosocial	3.275a	.108	3.061	3.489	
	prosocial	2.404a	.108	2.190	2.618	

a. 模型中出现的协变量在下列值处进行评估: age = 5.8723.	


4. gender * cartoon	
因变量	gender	cartoon	均值	标准 误差	95% 置信区间	
					下限	上限	
motivation	male	nonprosocial	3.175a	.204	2.772	3.578	
		prosocial	1.783a	.204	1.381	2.186	
	female	nonprosocial	3.305a	.204	2.902	3.708	
		prosocial	2.213a	.204	1.811	2.615	
后AB	male	nonprosocial	3.527a	.154	3.224	3.830	
		prosocial	2.166a	.153	1.863	2.468	
	female	nonprosocial	3.022a	.153	2.719	3.325	
		prosocial	2.642a	.153	2.340	2.945	

a. 模型中出现的协变量在下列值处进行评估: age = 5.8723.	

GLM motivation 后AB BY gender cartoon WITH age
  /METHOD=SSTYPE(3)
  /INTERCEPT=INCLUDE
  /EMMEANS=TABLES(OVERALL) WITH(age=MEAN)
  /EMMEANS=TABLES(gender) WITH(age=MEAN)
  /EMMEANS=TABLES(cartoon) WITH(age=MEAN) COMPARE(cartoon) ADJ(BONFERRONI)
  /EMMEANS=TABLES(gender*cartoon) WITH(age=MEAN)
  /PRINT=DESCRIPTIVE
  /CRITERIA=ALPHA(.05)
  /DESIGN=age gender cartoon gender*cartoon.


一般线性模型


附注	
创建的输出	25-SEP-2020 12:52:05	
注释		
输入	数据	F:\准备投稿的文章\目前投稿的文章\亲社会动画与攻击行为：动机中介-辣酱范式168\投搞材料\数据\实验数据\总数据.sav	
	活动的数据集	数据集1	
	过滤器	<none>	
	权重	<none>	
	拆分文件	<none>	
	工作数据文件中的 N 行	181	
缺失值处理	对缺失的定义	用户定义的缺失值作为缺失数据对待。	
	使用的案例	统计量的计算将基于带有有效数据的所有案例，而这些有效数据适用于模型中的所有变量。	
语法	GLM motivation 后AB BY gender cartoon WITH age
  /METHOD=SSTYPE(3)
  /INTERCEPT=INCLUDE
  /EMMEANS=TABLES(OVERALL) WITH(age=MEAN)
  /EMMEANS=TABLES(gender) WITH(age=MEAN)
  /EMMEANS=TABLES(cartoon) WITH(age=MEAN) COMPARE(cartoon) ADJ(BONFERRONI)
  /EMMEANS=TABLES(gender*cartoon) WITH(age=MEAN)
  /PRINT=DESCRIPTIVE
  /CRITERIA=ALPHA(.05)
  /DESIGN=age gender cartoon gender*cartoon.	
资源	处理器时间	00:00:00.03	
	已用时间	00:00:00.08	


[数据集1] F:\准备投稿的文章\目前投稿的文章\亲社会动画与攻击行为：动机中介-辣酱范式168\投搞材料\数据\实验数据\总数据.sav


主体间因子	
	值标签	N	
gender	1	male	84	
	2	female	84	
cartoon	0	nonprosocial	84	
	1	prosocial	84	


描述性统计量	
	gender	cartoon	均值	标准 偏差	N	
motivation	male	nonprosocial	3.1667	1.37781	42	
		prosocial	1.7857	1.17982	42	
		总计	2.4762	1.45185	84	
	female	nonprosocial	3.3095	1.48961	42	
		prosocial	2.2143	1.20032	42	
		总计	2.7619	1.45303	84	
	总计	nonprosocial	3.2381	1.42794	84	
		prosocial	2.0000	1.20241	84	
		总计	2.6190	1.45516	168	
后AB	male	nonprosocial	3.5238	1.06469	42	
		prosocial	2.1667	.98567	42	
		总计	2.8452	1.22714	84	
	female	nonprosocial	3.0238	.99971	42	
		prosocial	2.6429	.90585	42	
		总计	2.8333	.96734	84	
	总计	nonprosocial	3.2738	1.05683	84	
		prosocial	2.4048	.97089	84	
		总计	2.8393	1.10161	168	


多变量检验a	
效应	值	F	假设 df	误差 df	Sig.	
截距	Pillai 的跟踪	.070	6.091b	2.000	162.000	.003	
	Wilks 的 Lambda	.930	6.091b	2.000	162.000	.003	
	Hotelling 的跟踪	.075	6.091b	2.000	162.000	.003	
	Roy 的最大根	.075	6.091b	2.000	162.000	.003	
age	Pillai 的跟踪	.003	.231b	2.000	162.000	.794	
	Wilks 的 Lambda	.997	.231b	2.000	162.000	.794	
	Hotelling 的跟踪	.003	.231b	2.000	162.000	.794	
	Roy 的最大根	.003	.231b	2.000	162.000	.794	
gender	Pillai 的跟踪	.012	1.009b	2.000	162.000	.367	
	Wilks 的 Lambda	.988	1.009b	2.000	162.000	.367	
	Hotelling 的跟踪	.012	1.009b	2.000	162.000	.367	
	Roy 的最大根	.012	1.009b	2.000	162.000	.367	
cartoon	Pillai 的跟踪	.261	28.662b	2.000	162.000	.000	
	Wilks 的 Lambda	.739	28.662b	2.000	162.000	.000	
	Hotelling 的跟踪	.354	28.662b	2.000	162.000	.000	
	Roy 的最大根	.354	28.662b	2.000	162.000	.000	
gender * cartoon	Pillai 的跟踪	.059	5.091b	2.000	162.000	.007	
	Wilks 的 Lambda	.941	5.091b	2.000	162.000	.007	
	Hotelling 的跟踪	.063	5.091b	2.000	162.000	.007	
	Roy 的最大根	.063	5.091b	2.000	162.000	.007	

a. 设计 : 截距 + age + gender + cartoon + gender * cartoon	
b. 精确统计量	


主体间效应的检验	
源	因变量	III 型平方和	df	均方	F	Sig.	
校正模型	motivation	69.378a	4	17.345	9.946	.000	
	后AB	41.863b	4	10.466	10.609	.000	
截距	motivation	10.353	1	10.353	5.937	.016	
	后AB	8.622	1	8.622	8.740	.004	
age	motivation	.712	1	.712	.408	.524	
	后AB	.130	1	.130	.132	.717	
gender	motivation	3.287	1	3.287	1.885	.172	
	后AB	.009	1	.009	.009	.926	
cartoon	motivation	64.707	1	64.707	37.107	.000	
	后AB	31.809	1	31.809	32.244	.000	
gender * cartoon	motivation	.940	1	.940	.539	.464	
	后AB	10.099	1	10.099	10.238	.002	
误差	motivation	284.241	163	1.744			
	后AB	160.798	163	.986			
总计	motivation	1506.000	168				
	后AB	1557.000	168				
校正的总计	motivation	353.619	167				
	后AB	202.661	167				

a. R 方 = .196（调整 R 方 = .176）	
b. R 方 = .207（调整 R 方 = .187）	


估算边际均值


1. 总均值	
因变量	均值	标准 误差	95% 置信区间	
			下限	上限	
motivation	2.619a	.102	2.418	2.820	
后AB	2.839a	.077	2.688	2.991	

a. 模型中出现的协变量在下列值处进行评估: age = 5.8723.	


2. gender	
因变量	gender	均值	标准 误差	95% 置信区间	
				下限	上限	
motivation	male	2.479a	.144	2.194	2.764	
	female	2.759a	.144	2.474	3.044	
后AB	male	2.846a	.108	2.632	3.061	
	female	2.832a	.108	2.618	3.046	

a. 模型中出现的协变量在下列值处进行评估: age = 5.8723.	


3. cartoon


估计	
因变量	cartoon	均值	标准 误差	95% 置信区间	
				下限	上限	
motivation	nonprosocial	3.240a	.144	2.955	3.524	
	prosocial	1.998a	.144	1.714	2.283	
后AB	nonprosocial	3.275a	.108	3.061	3.489	
	prosocial	2.404a	.108	2.190	2.618	

a. 模型中出现的协变量在下列值处进行评估: age = 5.8723.	


成对比较	
因变量	(I) cartoon	(J) cartoon	均值差值 (I-J)	标准 误差	Sig.b	差分的 95% 置信区间b	
						下限	
motivation	nonprosocial	prosocial	1.242*	.204	.000	.839	
	prosocial	nonprosocial	-1.242*	.204	.000	-1.644	
后AB	nonprosocial	prosocial	.871*	.153	.000	.568	
	prosocial	nonprosocial	-.871*	.153	.000	-1.173	

成对比较	
因变量	(I) cartoon	(J) cartoon	差分的 95% 置信区间	
			上限	
motivation	nonprosocial	prosocial	1.644*	
	prosocial	nonprosocial	-.839*	
后AB	nonprosocial	prosocial	1.173*	
	prosocial	nonprosocial	-.568*	

基于估算边际均值	
*. 均值差值在 .05 级别上较显著。	
b. 对多个比较的调整： Bonferroni。	


多变量检验	
	值	F	假设 df	误差 df	Sig.	
Pillai 的跟踪	.261	28.662a	2.000	162.000	.000	
Wilks 的 lambda	.739	28.662a	2.000	162.000	.000	
Hotelling 的跟踪	.354	28.662a	2.000	162.000	.000	
Roy 的最大根	.354	28.662a	2.000	162.000	.000	

每个 F 检验 cartoon 的多变量效应。这些检验基于估算边际均值间的线性独立成对比较。	
a. 精确统计量	


单变量检验	
因变量	平方和	df	均方	F	Sig.	
motivation	对比	64.707	1	64.707	37.107	.000	
	误差	284.241	163	1.744			
后AB	对比	31.809	1	31.809	32.244	.000	
	误差	160.798	163	.986			

F 检验 cartoon 的效应。该检验基于估算边际均值间的线性独立成对比较。	


4. gender * cartoon	
因变量	gender	cartoon	均值	标准 误差	95% 置信区间	
					下限	上限	
motivation	male	nonprosocial	3.175a	.204	2.772	3.578	
		prosocial	1.783a	.204	1.381	2.186	
	female	nonprosocial	3.305a	.204	2.902	3.708	
		prosocial	2.213a	.204	1.811	2.615	
后AB	male	nonprosocial	3.527a	.154	3.224	3.830	
		prosocial	2.166a	.153	1.863	2.468	
	female	nonprosocial	3.022a	.153	2.719	3.325	
		prosocial	2.642a	.153	2.340	2.945	

a. 模型中出现的协变量在下列值处进行评估: age = 5.8723.	

GLM motivation 后AB BY gender cartoon WITH age
  /METHOD=SSTYPE(3)
  /INTERCEPT=INCLUDE
  /EMMEANS=TABLES(OVERALL) WITH(age=MEAN)
  /EMMEANS=TABLES(gender) WITH(age=MEAN)
  /EMMEANS=TABLES(cartoon) WITH(age=MEAN)
  /EMMEANS=TABLES(gender*cartoon) WITH(age=MEAN) COMPARE(gender) ADJ(BONFERRONI)
  /PRINT=DESCRIPTIVE
  /CRITERIA=ALPHA(.05)
  /DESIGN=age gender cartoon gender*cartoon.


一般线性模型


附注	
创建的输出	25-SEP-2020 12:52:34	
注释		
输入	数据	F:\准备投稿的文章\目前投稿的文章\亲社会动画与攻击行为：动机中介-辣酱范式168\投搞材料\数据\实验数据\总数据.sav	
	活动的数据集	数据集1	
	过滤器	<none>	
	权重	<none>	
	拆分文件	<none>	
	工作数据文件中的 N 行	181	
缺失值处理	对缺失的定义	用户定义的缺失值作为缺失数据对待。	
	使用的案例	统计量的计算将基于带有有效数据的所有案例，而这些有效数据适用于模型中的所有变量。	
语法	GLM motivation 后AB BY gender cartoon WITH age
  /METHOD=SSTYPE(3)
  /INTERCEPT=INCLUDE
  /EMMEANS=TABLES(OVERALL) WITH(age=MEAN)
  /EMMEANS=TABLES(gender) WITH(age=MEAN)
  /EMMEANS=TABLES(cartoon) WITH(age=MEAN)
  /EMMEANS=TABLES(gender*cartoon) WITH(age=MEAN) COMPARE(gender) ADJ(BONFERRONI)
  /PRINT=DESCRIPTIVE
  /CRITERIA=ALPHA(.05)
  /DESIGN=age gender cartoon gender*cartoon.	
资源	处理器时间	00:00:00.03	
	已用时间	00:00:00.03	


[数据集1] F:\准备投稿的文章\目前投稿的文章\亲社会动画与攻击行为：动机中介-辣酱范式168\投搞材料\数据\实验数据\总数据.sav


主体间因子	
	值标签	N	
gender	1	male	84	
	2	female	84	
cartoon	0	nonprosocial	84	
	1	prosocial	84	


描述性统计量	
	gender	cartoon	均值	标准 偏差	N	
motivation	male	nonprosocial	3.1667	1.37781	42	
		prosocial	1.7857	1.17982	42	
		总计	2.4762	1.45185	84	
	female	nonprosocial	3.3095	1.48961	42	
		prosocial	2.2143	1.20032	42	
		总计	2.7619	1.45303	84	
	总计	nonprosocial	3.2381	1.42794	84	
		prosocial	2.0000	1.20241	84	
		总计	2.6190	1.45516	168	
后AB	male	nonprosocial	3.5238	1.06469	42	
		prosocial	2.1667	.98567	42	
		总计	2.8452	1.22714	84	
	female	nonprosocial	3.0238	.99971	42	
		prosocial	2.6429	.90585	42	
		总计	2.8333	.96734	84	
	总计	nonprosocial	3.2738	1.05683	84	
		prosocial	2.4048	.97089	84	
		总计	2.8393	1.10161	168	


多变量检验a	
效应	值	F	假设 df	误差 df	Sig.	
截距	Pillai 的跟踪	.070	6.091b	2.000	162.000	.003	
	Wilks 的 Lambda	.930	6.091b	2.000	162.000	.003	
	Hotelling 的跟踪	.075	6.091b	2.000	162.000	.003	
	Roy 的最大根	.075	6.091b	2.000	162.000	.003	
age	Pillai 的跟踪	.003	.231b	2.000	162.000	.794	
	Wilks 的 Lambda	.997	.231b	2.000	162.000	.794	
	Hotelling 的跟踪	.003	.231b	2.000	162.000	.794	
	Roy 的最大根	.003	.231b	2.000	162.000	.794	
gender	Pillai 的跟踪	.012	1.009b	2.000	162.000	.367	
	Wilks 的 Lambda	.988	1.009b	2.000	162.000	.367	
	Hotelling 的跟踪	.012	1.009b	2.000	162.000	.367	
	Roy 的最大根	.012	1.009b	2.000	162.000	.367	
cartoon	Pillai 的跟踪	.261	28.662b	2.000	162.000	.000	
	Wilks 的 Lambda	.739	28.662b	2.000	162.000	.000	
	Hotelling 的跟踪	.354	28.662b	2.000	162.000	.000	
	Roy 的最大根	.354	28.662b	2.000	162.000	.000	
gender * cartoon	Pillai 的跟踪	.059	5.091b	2.000	162.000	.007	
	Wilks 的 Lambda	.941	5.091b	2.000	162.000	.007	
	Hotelling 的跟踪	.063	5.091b	2.000	162.000	.007	
	Roy 的最大根	.063	5.091b	2.000	162.000	.007	

a. 设计 : 截距 + age + gender + cartoon + gender * cartoon	
b. 精确统计量	


主体间效应的检验	
源	因变量	III 型平方和	df	均方	F	Sig.	
校正模型	motivation	69.378a	4	17.345	9.946	.000	
	后AB	41.863b	4	10.466	10.609	.000	
截距	motivation	10.353	1	10.353	5.937	.016	
	后AB	8.622	1	8.622	8.740	.004	
age	motivation	.712	1	.712	.408	.524	
	后AB	.130	1	.130	.132	.717	
gender	motivation	3.287	1	3.287	1.885	.172	
	后AB	.009	1	.009	.009	.926	
cartoon	motivation	64.707	1	64.707	37.107	.000	
	后AB	31.809	1	31.809	32.244	.000	
gender * cartoon	motivation	.940	1	.940	.539	.464	
	后AB	10.099	1	10.099	10.238	.002	
误差	motivation	284.241	163	1.744			
	后AB	160.798	163	.986			
总计	motivation	1506.000	168				
	后AB	1557.000	168				
校正的总计	motivation	353.619	167				
	后AB	202.661	167				

a. R 方 = .196（调整 R 方 = .176）	
b. R 方 = .207（调整 R 方 = .187）	


估算边际均值


1. 总均值	
因变量	均值	标准 误差	95% 置信区间	
			下限	上限	
motivation	2.619a	.102	2.418	2.820	
后AB	2.839a	.077	2.688	2.991	

a. 模型中出现的协变量在下列值处进行评估: age = 5.8723.	


2. gender	
因变量	gender	均值	标准 误差	95% 置信区间	
				下限	上限	
motivation	male	2.479a	.144	2.194	2.764	
	female	2.759a	.144	2.474	3.044	
后AB	male	2.846a	.108	2.632	3.061	
	female	2.832a	.108	2.618	3.046	

a. 模型中出现的协变量在下列值处进行评估: age = 5.8723.	


3. cartoon	
因变量	cartoon	均值	标准 误差	95% 置信区间	
				下限	上限	
motivation	nonprosocial	3.240a	.144	2.955	3.524	
	prosocial	1.998a	.144	1.714	2.283	
后AB	nonprosocial	3.275a	.108	3.061	3.489	
	prosocial	2.404a	.108	2.190	2.618	

a. 模型中出现的协变量在下列值处进行评估: age = 5.8723.	


4. gender * cartoon


估计	
因变量	gender	cartoon	均值	标准 误差	95% 置信区间	
					下限	上限	
motivation	male	nonprosocial	3.175a	.204	2.772	3.578	
		prosocial	1.783a	.204	1.381	2.186	
	female	nonprosocial	3.305a	.204	2.902	3.708	
		prosocial	2.213a	.204	1.811	2.615	
后AB	male	nonprosocial	3.527a	.154	3.224	3.830	
		prosocial	2.166a	.153	1.863	2.468	
	female	nonprosocial	3.022a	.153	2.719	3.325	
		prosocial	2.642a	.153	2.340	2.945	

a. 模型中出现的协变量在下列值处进行评估: age = 5.8723.	


成对比较	
因变量	cartoon	(I) gender	(J) gender	均值差值 (I-J)	标准 误差	Sig.b	
							
motivation	nonprosocial	male	female	-.130	.289	.653	
		female	male	.130	.289	.653	
	prosocial	male	female	-.430	.288	.138	
		female	male	.430	.288	.138	
后AB	nonprosocial	male	female	.505*	.217	.021	
		female	male	-.505*	.217	.021	
	prosocial	male	female	-.477*	.217	.029	
		female	male	.477*	.217	.029	

成对比较	
因变量	cartoon	(I) gender	(J) gender	差分的 95% 置信区间	
				下限	上限	
motivation	nonprosocial	male	female	-.701	.440	
		female	male	-.440	.701	
	prosocial	male	female	-.999	.139	
		female	male	-.139	.999	
后AB	nonprosocial	male	female	.076*	.934	
		female	male	-.934*	-.076	
	prosocial	male	female	-.905*	-.049	
		female	male	.049*	.905	

基于估算边际均值	
*. 均值差值在 .05 级别上较显著。	
b. 对多个比较的调整： Bonferroni。	


多变量检验	
cartoon	值	F	假设 df	误差 df	Sig.	
nonprosocial	Pillai 的跟踪	.037	3.132a	2.000	162.000	.046	
	Wilks 的 lambda	.963	3.132a	2.000	162.000	.046	
	Hotelling 的跟踪	.039	3.132a	2.000	162.000	.046	
	Roy 的最大根	.039	3.132a	2.000	162.000	.046	
prosocial	Pillai 的跟踪	.035	2.970a	2.000	162.000	.054	
	Wilks 的 lambda	.965	2.970a	2.000	162.000	.054	
	Hotelling 的跟踪	.037	2.970a	2.000	162.000	.054	
	Roy 的最大根	.037	2.970a	2.000	162.000	.054	

每个 F 在其他显示效应的每个级别组合中检验 gender 的多变量简单效应。 这些检验基于估算边际均值间的线性独立成对比较。	
a. 精确统计量	


单变量检验	
因变量	cartoon	平方和	df	均方	F	Sig.	
motivation	nonprosocial	对比	.355	1	.355	.203	.653	
		误差	284.241	163	1.744			
	prosocial	对比	3.879	1	3.879	2.225	.138	
		误差	284.241	163	1.744			
后AB	nonprosocial	对比	5.339	1	5.339	5.412	.021	
		误差	160.798	163	.986			
	prosocial	对比	4.772	1	4.772	4.838	.029	
		误差	160.798	163	.986			

每个 F 在其他显示效应的每个级别组合中检验 gender 的简单效应。 这些检验基于估算边际均值间的线性独立成对比较。	

GLM motivation 后AB BY gender cartoon WITH age
  /METHOD=SSTYPE(3)
  /INTERCEPT=INCLUDE
  /PLOT=PROFILE(cartoon cartoon*gender)
  /EMMEANS=TABLES(OVERALL) WITH(age=MEAN)
  /EMMEANS=TABLES(gender) WITH(age=MEAN)
  /EMMEANS=TABLES(cartoon) WITH(age=MEAN)
  /EMMEANS=TABLES(gender*cartoon) WITH(age=MEAN)
  /PRINT=DESCRIPTIVE
  /CRITERIA=ALPHA(.05)
  /DESIGN=age gender cartoon gender*cartoon.


一般线性模型


附注	
创建的输出	25-SEP-2020 12:54:13	
注释		
输入	数据	F:\准备投稿的文章\目前投稿的文章\亲社会动画与攻击行为：动机中介-辣酱范式168\投搞材料\数据\实验数据\总数据.sav	
	活动的数据集	数据集1	
	过滤器	<none>	
	权重	<none>	
	拆分文件	<none>	
	工作数据文件中的 N 行	181	
缺失值处理	对缺失的定义	用户定义的缺失值作为缺失数据对待。	
	使用的案例	统计量的计算将基于带有有效数据的所有案例，而这些有效数据适用于模型中的所有变量。	
语法	GLM motivation 后AB BY gender cartoon WITH age
  /METHOD=SSTYPE(3)
  /INTERCEPT=INCLUDE
  /PLOT=PROFILE(cartoon cartoon*gender)
  /EMMEANS=TABLES(OVERALL) WITH(age=MEAN)
  /EMMEANS=TABLES(gender) WITH(age=MEAN)
  /EMMEANS=TABLES(cartoon) WITH(age=MEAN)
  /EMMEANS=TABLES(gender*cartoon) WITH(age=MEAN)
  /PRINT=DESCRIPTIVE
  /CRITERIA=ALPHA(.05)
  /DESIGN=age gender cartoon gender*cartoon.	
资源	处理器时间	00:00:01.88	
	已用时间	00:00:01.41	


[数据集1] F:\准备投稿的文章\目前投稿的文章\亲社会动画与攻击行为：动机中介-辣酱范式168\投搞材料\数据\实验数据\总数据.sav


主体间因子	
	值标签	N	
gender	1	male	84	
	2	female	84	
cartoon	0	nonprosocial	84	
	1	prosocial	84	


描述性统计量	
	gender	cartoon	均值	标准 偏差	N	
motivation	male	nonprosocial	3.1667	1.37781	42	
		prosocial	1.7857	1.17982	42	
		总计	2.4762	1.45185	84	
	female	nonprosocial	3.3095	1.48961	42	
		prosocial	2.2143	1.20032	42	
		总计	2.7619	1.45303	84	
	总计	nonprosocial	3.2381	1.42794	84	
		prosocial	2.0000	1.20241	84	
		总计	2.6190	1.45516	168	
后AB	male	nonprosocial	3.5238	1.06469	42	
		prosocial	2.1667	.98567	42	
		总计	2.8452	1.22714	84	
	female	nonprosocial	3.0238	.99971	42	
		prosocial	2.6429	.90585	42	
		总计	2.8333	.96734	84	
	总计	nonprosocial	3.2738	1.05683	84	
		prosocial	2.4048	.97089	84	
		总计	2.8393	1.10161	168	


多变量检验a	
效应	值	F	假设 df	误差 df	Sig.	
截距	Pillai 的跟踪	.070	6.091b	2.000	162.000	.003	
	Wilks 的 Lambda	.930	6.091b	2.000	162.000	.003	
	Hotelling 的跟踪	.075	6.091b	2.000	162.000	.003	
	Roy 的最大根	.075	6.091b	2.000	162.000	.003	
age	Pillai 的跟踪	.003	.231b	2.000	162.000	.794	
	Wilks 的 Lambda	.997	.231b	2.000	162.000	.794	
	Hotelling 的跟踪	.003	.231b	2.000	162.000	.794	
	Roy 的最大根	.003	.231b	2.000	162.000	.794	
gender	Pillai 的跟踪	.012	1.009b	2.000	162.000	.367	
	Wilks 的 Lambda	.988	1.009b	2.000	162.000	.367	
	Hotelling 的跟踪	.012	1.009b	2.000	162.000	.367	
	Roy 的最大根	.012	1.009b	2.000	162.000	.367	
cartoon	Pillai 的跟踪	.261	28.662b	2.000	162.000	.000	
	Wilks 的 Lambda	.739	28.662b	2.000	162.000	.000	
	Hotelling 的跟踪	.354	28.662b	2.000	162.000	.000	
	Roy 的最大根	.354	28.662b	2.000	162.000	.000	
gender * cartoon	Pillai 的跟踪	.059	5.091b	2.000	162.000	.007	
	Wilks 的 Lambda	.941	5.091b	2.000	162.000	.007	
	Hotelling 的跟踪	.063	5.091b	2.000	162.000	.007	
	Roy 的最大根	.063	5.091b	2.000	162.000	.007	

a. 设计 : 截距 + age + gender + cartoon + gender * cartoon	
b. 精确统计量	


主体间效应的检验	
源	因变量	III 型平方和	df	均方	F	Sig.	
校正模型	motivation	69.378a	4	17.345	9.946	.000	
	后AB	41.863b	4	10.466	10.609	.000	
截距	motivation	10.353	1	10.353	5.937	.016	
	后AB	8.622	1	8.622	8.740	.004	
age	motivation	.712	1	.712	.408	.524	
	后AB	.130	1	.130	.132	.717	
gender	motivation	3.287	1	3.287	1.885	.172	
	后AB	.009	1	.009	.009	.926	
cartoon	motivation	64.707	1	64.707	37.107	.000	
	后AB	31.809	1	31.809	32.244	.000	
gender * cartoon	motivation	.940	1	.940	.539	.464	
	后AB	10.099	1	10.099	10.238	.002	
误差	motivation	284.241	163	1.744			
	后AB	160.798	163	.986			
总计	motivation	1506.000	168				
	后AB	1557.000	168				
校正的总计	motivation	353.619	167				
	后AB	202.661	167				

a. R 方 = .196（调整 R 方 = .176）	
b. R 方 = .207（调整 R 方 = .187）	


估算边际均值


1. 总均值	
因变量	均值	标准 误差	95% 置信区间	
			下限	上限	
motivation	2.619a	.102	2.418	2.820	
后AB	2.839a	.077	2.688	2.991	

a. 模型中出现的协变量在下列值处进行评估: age = 5.8723.	


2. gender	
因变量	gender	均值	标准 误差	95% 置信区间	
				下限	上限	
motivation	male	2.479a	.144	2.194	2.764	
	female	2.759a	.144	2.474	3.044	
后AB	male	2.846a	.108	2.632	3.061	
	female	2.832a	.108	2.618	3.046	

a. 模型中出现的协变量在下列值处进行评估: age = 5.8723.	


3. cartoon	
因变量	cartoon	均值	标准 误差	95% 置信区间	
				下限	上限	
motivation	nonprosocial	3.240a	.144	2.955	3.524	
	prosocial	1.998a	.144	1.714	2.283	
后AB	nonprosocial	3.275a	.108	3.061	3.489	
	prosocial	2.404a	.108	2.190	2.618	

a. 模型中出现的协变量在下列值处进行评估: age = 5.8723.	


4. gender * cartoon	
因变量	gender	cartoon	均值	标准 误差	95% 置信区间	
					下限	上限	
motivation	male	nonprosocial	3.175a	.204	2.772	3.578	
		prosocial	1.783a	.204	1.381	2.186	
	female	nonprosocial	3.305a	.204	2.902	3.708	
		prosocial	2.213a	.204	1.811	2.615	
后AB	male	nonprosocial	3.527a	.154	3.224	3.830	
		prosocial	2.166a	.153	1.863	2.468	
	female	nonprosocial	3.022a	.153	2.719	3.325	
		prosocial	2.642a	.153	2.340	2.945	

a. 模型中出现的协变量在下列值处进行评估: age = 5.8723.	


概要文件图


motivation


后AB
