# Supplementary material for: Positive Effects of Prosocial Cartoon Viewing on Aggression Among Children: The Potential Mediating Role of Aggressive Motivation
Source: Front Psychol. 2021 Dec 22;12:742568. doi: 10.3389/fpsyg.2021.742568 (PMC8782158; doi:10.3389/fpsyg.2021.742568)
Supplement: Supplementary file 4 [file Data_Sheet_4.doc]

* Encoding: windows-1252.
/* PROCESS version 3.0 */.
/* Written by Andrew F. Hayes */.
/* www.afhayes.com */.
/* www.processmacro.org */.
/* Copyright 2018 by Andrew F. Hayes */.
/* Documented in http://www.guilford.com/p/hayes3 */.
/* PROCESS workshop schedule at http://www.processmacro.org/workshops.html */.
/* Distribution of this code in any form, except through processmacro.org, is prohibited */.
/* without the permission of the copyright holder */.
/* THIS SOFTWARE IS PROVIDED "AS IS", WITHOUT WARRANTY OF ANY KIND */.
/* EXPRESS OR IMPLIED, INCLUDING BUT NOT LIMITED TO THE WARRANTIES OF */.
/* MERCHANTABILITY, FITNESS FOR A PARTICULAR PURPOSE AND NONINFRINGEMENT */.
/* IN NO EVENT SHALL THE COPYRIGHT HOLDERS BE LIABLE FOR ANY CLAIM, */.
/* DAMAGES OR OTHER LIABILITY, WHETHER IN AN ACTION OF CONTRACT, TORT */.
/* OR OTHERWISE, ARISING FROM, OUT OF OR IN CONNECTION WITH THE */.
/* SOFTWARE OR THE USE OR OTHER DEALINGS IN THE SOFTWARE */.
/* USE OF THIS SOFTWARE IMPLIES AGREEMENT WITH THESE TERMS */.
set printback=off.


矩阵


附注	
创建的输出	23-OCT-2020 10:23:46	
注释		
输入	数据	F:\准备投稿的文章\目前投稿的文章\亲社会动画与攻击行为：动机中介-辣酱范式168\投搞材料word版\男生.sav	
	活动的数据集	数据集1	
	过滤器	<none>	
	权重	<none>	
	拆分文件	<none>	
	工作数据文件中的 N 行	97	
语法	MATRIX.
compute errcode=make(100,1,0).
compute notecode=make(100,1,0).
compute model = trunc( 4 ).
compute maxwwarn=0.
compute minwwarn=0.
compute maxzwarn=0.
compute minzwarn=0.
compute toomany=0.
compute wdich=0.
compute zdich=0.
compute wnotev=0.
compute znotev=0.
compute nxpval=1.
compute nwpval=1.
compute nzpval=1.
compute errs=1.
compute notes=1.
compute criterr=0.
compute novar=0.
compute adjust=0.
compute ncs=0.
compute serial=0.
compute sobelok=0.
compute hasw=0.
compute hasz=0.
compute printw=0.
compute printz=0.
compute counterf=0.
compute wmodcust=0.
compute zmodcust=0.
compute cov = 'age'.
compute varorder=( 0 <> 0).
compute nws=0.
compute w= 'xxxxx'.
compute nzs=0.
compute z = 'xxxxx'.
compute nms=0.
compute m = 'Zmotivation'.
compute nys=0.
compute y = 'Z后AB'.
compute nxs=0.
compute x = 'Zcartoon'.
compute v = 'xxxxx'.
compute q = 'xxxxx'.
compute mcxok=0.
compute mcwok=0.
compute mczok=0.
compute xprod=0.
compute zprod=0.
compute wprod=0.
compute modcok=0.
compute hc3=trunc( 0 ).
compute jn=( 0 = 1).
compute effsize=( 0 =1).
compute normal=( 0 =1).
compute sobelok=0.
compute normal=( 0 =1).
compute mdichok=( 0 =1).
compute ydichok=( 0 =1).
compute contrast={ 999 }.
compute ncontr=ncol(contrast).
compute ncontrow=nrow(contrast).
do if (contrast(1,1) = 999).
compute ncontr=1.
compute contrast=0.
end if.
do if (ncontr = 1).
compute contrast=trunc(contrast).
do if (contrast > 2 or contrast < 0)).
compute ncontr=1.
compute contrast = 0.
end if.
end if.
do if (ncontr > 1).
compute contvec=contrast.
compute contrast=3.
do if (ncontrow > 1).
compute contrast=0.
compute modcok=1.
compute wcontval=contvec(:,1).
compute zcontval=contvec(:,2).
do if ((ncontr <> 2) or (ncontrow <> 2)).
compute notecode(notes,1) = 19.
compute notes = notes + 1.
compute modcok=0.
end if.
end if.
end if.
do if (varorder = 1).
compute notecode(notes,1) = 21.
compute notes = notes + 1.
end if.
do if ( 999 <> 999 or 999 <> 999).
compute notecode(notes,1) = 22.
compute notes = notes + 1.
end if.
compute modelbt=( 0 =1).
compute cluster= 'xxxxx'.
compute matrices=( 0 =1).
compute covcoeff=( 0 =1).
compute covmy=trunc( 0 ).
do if (covmy < 0 or covmy > 2).
compute covmy = 0.
end if.
compute boot = abs(trunc( 5000 )).
compute mc=abs(trunc( 0 )).
compute hc=trunc( 5 ).
compute intprobe = .1.
do if (intprobe < 0 or intprobe > 1).
compute intprobe = .10.
end if.
compute plot=trunc( 0 ).
do if (plot < 0 or plot > 2).
compute plot=0.
end if.
compute total=( 0 =1).
compute dototal=0.
compute saveboot = ( 0 = 1).
compute saveest=( 0 = 2).
do if (hc >= 0 and hc < 5).
compute notecode(notes,1) = 4.
compute notes = notes + 1.
end if.
do if (hc > 5 or hc < 0).
compute hc=5.
end if.
compute mcw=trunc( 0 ).
compute mcz=trunc( 0 ).
compute mcx=trunc( 0 ).
do if (mcx > 0 and model = 74).
compute mcw=mcx.
end if.
compute nxvls=1.
compute nmvls=1.
compute nwvls=1.
compute nzvls=1.
compute paths=999.
compute pathsw=999.
compute pathsz=999.
compute pathswz=999.
compute pathsmod=999.
compute pathtype=999.
compute obscoeff=999.
compute pathsdv={' '}.
compute quantile=1.
do if ( 999 <>999).
compute notecode(notes,1) = 23.
compute notes = notes + 1.
end if.
compute moments=( 0 =1).
do if (moments=1).
compute quantile=0.
end if.
compute center=( 0 =1).
compute bmatrix={ -999 }.
compute wmatrix={ -999 }.
compute zmatrix={ -999 }.
compute wzmatrix={ -999 }.
compute cmatrix={ -999 }.
compute xcatcode={ -999 }.
compute wcatcode={ -999 }.
compute zcatcode={ -999 }.
compute needed=0.
compute conf= 95.
do if (trunc( 95 ) >= 100 or (trunc( 95 ) <= 50)).
compute conf = 95.
compute notecode(notes,1)=2.
compute notes=notes+1.
end if.
do if (model > 0 and model < 4 and modelbt=0).
compute boot=0.
compute mc=0.
end if.
do if (boot > 0 and mc > 0).
compute boot=0.
end if.
compute p0=-.322232431088.
compute p1 = -1.
compute p2 = -.342242088547.
compute p3 = -.0204231210245.
compute p4 = -.0000453642210148.
compute q0 = .0993484626060.
compute q1 = .588581570495.
compute q2 = .531103462366.
compute q3 = .103537752850.
compute q4 = .0038560700634.
compute alpha2 = (1-(conf/100))/2.
compute cilm=alpha2*2.
compute y5=sqrt(-2*ln(alpha2)).
compute xp2=(y5+((((y5*p4+p3)*y5+p2)*y5+p1)*y5+p0)/((((y5*q4+q3)*y5+q2)*y5+q1)*y5+q0)).
compute medlb={'   M1  :';'   M2  :';'   M3  :';'   M4  :';'   M5  :';'   M6  :';'   M7  :';'   M8  :';'   M9  :';'   M10 :'}.
compute medlb2={'(M1)','(M2)','(M3)','(M4)','(M5)','(M6)','(M7)','(M8)','(M9)','(M10)'}.
compute xlb={'   X1  :';'   X2  :';'   X3  :';'   X4  :';'   X5  :';'   X6  :';'   X7  :';'   X8  :';'   X9  :'}.
compute highlbw={'M1*W'; 'M2*W'; 'M3*W'; 'M4*W'; 'M5*W'; 'M6*W'; 'M7*W'; 'M8*W'; 'M9*W'; 'M10*W'}.
compute highlbz={'M1*Z'; 'M2*Z'; 'M3*Z'; 'M4*Z'; 'M5*Z'; 'M6*Z'; 'M7*Z'; 'M8*Z'; 'M9*Z';'M10*Z'}.
compute highlbwz={'M1*W*Z'; 'M2*W*Z'; 'M3*W*Z'; 'M4*W*Z'; 'M5*W*Z'; 'M6*W*Z'; 'M7*W*Z'; 'M8*W*Z'; 'M9*W*Z';'M10*W*Z'}.
compute skipwz=0.
compute validm={1,1,1,1,1,1,1,1,1,1,1,1,1,1,1,1,1,1,1,1,1,1,0,0,0,0,0,1,1,0,0,0,0, 0,0,0,0,0,0,0,0,0,0,0,0,0,0,0,0,0,0,0,0,0,0,0,0,1,1,1,1,1,1,1,1,1,1,1,1,1,1,1,1,0,1,1,0,0,0,1,1,1,1,1,1,1,1,1,1,1,1,1}.
do if (( 0 =1)=1).
compute errcode(errs,1)=42.
compute errs=errs+1.
compute criterr=1.
end if.
do if (model > 0 and model < 93).
do if (validm(1,model)=0).
do if (model <> 74).
compute errcode(errs,1)=6.
compute errs=errs+1.
compute criterr=1.
end if.
do if (model = 74).
compute errcode(errs,1)=46.
compute errs=errs+1.
compute criterr=1.
end if.
end if.
release validm.
end if.
do if ((model > 92 or model < 0) and model <> 999)).
compute errcode(errs,1)=7.
compute errs=errs+1.
compute criterr=1.
end if.
do if (model = 999 and bmatrix(1,1)=-999).
compute errcode(errs,1)=24.
compute errs=errs+1.
compute criterr=1.
end if.
do if (model <> 999 and bmatrix(1,1) <> -999).
compute errcode(errs,1)=25.
compute errs=errs+1.
compute criterr=1.
end if.
do if ((model = 74 or (model > 0 and model < 4)) and ((wmatrix(1,1) <> -999) or (zmatrix(1,1)<>-999) or (wzmatrix(1,1)<>-999)))).
compute errcode(errs,1)=41.
compute errs=errs+1.
compute criterr=1.
end if.
do if (hc3 <> 0).
compute notecode(notes,1) = 5.
compute notes = notes + 1.
do if (hc3 = 1).
compute hc=3.
end if.
end if.
do if ((v <> 'xxxxx') or (q <> 'xxxxx')).
compute errcode(errs,1)=14.
compute errs=errs+1.
compute criterr=1.
end if.
do if (cluster <> 'xxxxx').
compute errcode(errs,1)=27.
compute errs=errs+1.
compute criterr=1.
end if.
do if ((y = 'xxxxx') or (x = 'xxxxx')).
compute errcode(errs,1)=1.
compute errs=errs+1.
compute criterr=1.
end if.
do if ((m = 'xxxxx') and model > 3).
compute errcode(errs,1)=8.
compute errs=errs+1.
compute criterr=1.
end if.
do if (criterr=0).
get ytmp/variables = Z后AB /names = ynames/MISSING = 99999.
compute nys=ncol(ytmp).
compute needed=nys.
compute n=nrow(ytmp).
compute varnames={ynames}.
compute dat=ytmp.

.


.
compute modelvar={ '4' ;t(ynames)}.
do if ( 4 =999).
compute modelvar(1,1)='CUSTOM'.
end if.
get xtmp/variables = Zcartoon /names = xnames/MISSING = 99999.
compute nxs=ncol(xtmp).
compute n=nrow(xtmp).
compute needed=needed+nxs.
compute varnames={varnames,xnames}.
compute xcatlab=t(xnames).
compute dat={dat,xtmp}.

.


.
compute modelvar={modelvar;t(xnames)}.
do if (nxs = 1).
compute modelvlb={'Model  :';'    Y  :';'    X  :'}.
else.
compute modelvlb={'Model  :';'    Y  :';xlb(1:nxs,1)}.
end if.
do if (m <> 'xxxxx').
get mtmp/variables = Zmotivation /names = mnames/MISSING = 99999.
compute nms=ncol(mtmp).
compute mprod=make(1,nms,0).
compute n=nrow(mtmp).
compute needed=needed+nms.
compute varnames={varnames,mnames}.
compute dat={dat,mtmp}.
compute modelvar={modelvar;t(mnames)}.
compute x2m=make(99,nms,0).
compute m2y=make(99,nms,0).
compute onem=make(nms,1,1).

.


compute toomany=1.


.
do if (nms > 1 and nms < 11).
compute modelvlb={modelvlb;medlb(1:nms,1)}.
else.
compute modelvlb={modelvlb;'    M  :'}.
end if.
do if (nms > 0 and model < 4).
compute errcode(errs,1)=9.
compute errs=errs+1.
compute criterr=1.
end if.
end if.
compute wlocatet=0.
compute wlocate=0.
do if (w <> 'xxxxx').
get wtmp/variables = xxxxx /names = wnames/MISSING = 99999.
compute nws=ncol(wtmp).
compute n=nrow(wtmp).

.


.
compute varnames={varnames,wnames}.
compute wlocate=ncol(varnames).
do if (model=74).
compute wlocatet=1.
do if (xnames <> wnames).
compute errcode(errs,1)=45.
compute errs=errs+1.
compute criterr=1.
end if.
end if.
compute wcatlab=t(wnames).
compute dat={dat,wtmp}.
compute modelvar={modelvar;t(wnames)}.
compute modelvlb={modelvlb;'    W  :'}.
end if.
do if (z <> 'xxxxx').
get ztmp/variables = xxxxx /names = znames/MISSING = 99999.
compute nzs=ncol(ztmp).
compute n=nrow(ztmp).

.


.
compute varnames={varnames,znames}.
compute zcatlab=t(znames).
compute dat={dat,ztmp}.
compute modelvar={modelvar;t(znames)}.
compute modelvlb={modelvlb;'    Z  :'}.
end if.
do if (cov <> 'xxxxx').
get ctmp/variables = age /names = covnames/MISSING = 99999.
compute ncs=ncol(ctmp).
compute n=nrow(ctmp).

.


.
compute varnames={varnames,covnames}.
compute dat={dat,ctmp}.
end if.
do if (nws > 1 or nzs > 1 or nys > 1 or nxs > 1).
compute errcode(errs,1)=3.
compute errs=errs+1.
compute criterr=1.
end if.
do if ((model = 80 or model = 81) and (nms < 3 or nms > 6)).
compute errcode(errs,1)=32.
compute errs=errs+1.
compute criterr=1.
end if.
do if (model = 82 and nms <> 4).
compute errcode(errs,1)=33.
compute errs=errs+1.
compute criterr=1.
end if.
do if (nms > 10).
compute errcode(errs,1)=37.
compute errs=errs+1.
compute criterr=1.
end if.
do if ((model = 6 or (model > 82 and model < 999)) and (nms < 2 or nms > 6)).
compute errcode(errs,1)=34.
compute errs=errs+1.
compute criterr=1.
end if.
compute match=0.
loop i = 1 to (ncol(varnames)-1).
loop j = (i+1) to ncol(varnames).
do if (varnames(i)=varnames(j)).
compute match=1.
do if (wlocatet=1 and i=2 and j=wlocate).
compute match=0.
end if.
end if.
end loop.
end loop.
do if (match=1).
compute errcode(errs,1)=2.
compute errs=errs+1.
compute criterr=1.
end if.
compute ninit=nrow(dat).
compute rownum=make(ninit,1,0).
loop i = 1 to ninit.
compute rownum(i,1)=i.
end loop.
compute dat={rownum,dat}.
compute j=1.
compute missrow=0.
loop i = 1 to n.
do if (rsum(dat(i,2:ncol(dat))=99999)=0).
compute dat(j,:)=dat(i,:).
compute j=j+1.
else.
compute missrow={missrow;dat(i,1)}.
end if.
end loop.
compute rownum=dat(1:(j-1),1).
do if (nrow(missrow) > 1).
compute missrow=t(missrow(2:nrow(missrow),1)).
end if.
compute dat=dat(1:(j-1),2:ncol(dat)).
compute n=nrow(dat).
compute nmiss=ninit-n.
compute ytmp=dat(:,1:nys).

.
compute desctmp=make((8-(4* 0 )),ncol( ytmp ),-999).
loop jd=1 to ncol( ytmp ).
compute descdat= ytmp (:,jd).
compute desctmp(1,jd) = csum(descdat)/nrow(descdat).
compute desctmp(2,jd) = (nrow(descdat)*sscp(descdat))-(t(csum(descdat))*(csum(descdat))).
compute desctmp(2,jd) = sqrt(desctmp(2,jd)/(nrow(descdat)*(nrow(descdat)-1))).
compute desctmp(3,jd)=cmin(descdat).
compute desctmp(4,jd)=cmax(descdat).
do if ( 0 =0).
compute minwarn=0.
compute maxwarn=0.
do if ((desctmp(3,jd)=desctmp(4,jd)) and novar=0).
compute errcode(errs,1)=15.
compute errs=errs+1.
compute criterr=1.
compute novar=1.
end if.
compute tmp=((descdat(:,1)=desctmp(3,jd))+(descdat(:,1)=desctmp(4,jd))).
compute desctmp(8,jd)=(csum(tmp)=nrow(tmp)).
compute tmp = descdat.
compute tmp(GRADE(descdat),:) = descdat.
compute descdat = tmp.
release tmp.
compute decval={.16;.5;.84}.
loop kd=1 to 3.
compute low=trunc(decval(kd,1)*(nrow(descdat)+1)).
compute lowdec=decval(kd,1)*(nrow(descdat)+1)-low.
compute value=descdat(low,1)+(descdat((low+1),1)-descdat(low,1))*lowdec.
compute desctmp((4+kd),jd)=value.
end loop.
compute mnotev=1.
compute modvals=desctmp(5:7,:).
do if (quantile <> 1).
compute desctmp(5,jd)=desctmp(1,jd)-desctmp(2,jd).
compute desctmp(6,jd)=desctmp(1,jd).
compute desctmp(7,jd)=desctmp(1,jd)+desctmp(2,jd).
compute modvals=desctmp(5:7,:).
compute mnotev=2.
do if (modvals(1,1) < desctmp(3,1)).
compute modvals(1,1)=desctmp(3,1).
compute minwarn=1.
end if.
do if (modvals(3,1) > desctmp(4,1)).
compute modvals(3,1)=desctmp(4,1).
compute maxwarn=1.
end if.
end if.
do if (desctmp(8,1)=1).
compute modvals={desctmp(3,1);desctmp(4,1)}.
compute mnotev=0.
compute minwarn=0.
compute maxwarn=0.
end if.
end if.
end loop
.
compute ysd=desctmp(2,:).
do if ((desctmp(8,1)=1) and (ydichok <> 1)).
compute errcode(errs,1)=44.
compute errs=errs+1.
compute criterr=1.
end if.
compute xtmp=dat(:,(nys+1):(nys+nxs)).

.
compute desctmp=make((8-(4* 0 )),ncol( xtmp ),-999).
loop jd=1 to ncol( xtmp ).
compute descdat= xtmp (:,jd).
compute desctmp(1,jd) = csum(descdat)/nrow(descdat).
compute desctmp(2,jd) = (nrow(descdat)*sscp(descdat))-(t(csum(descdat))*(csum(descdat))).
compute desctmp(2,jd) = sqrt(desctmp(2,jd)/(nrow(descdat)*(nrow(descdat)-1))).
compute desctmp(3,jd)=cmin(descdat).
compute desctmp(4,jd)=cmax(descdat).
do if ( 0 =0).
compute minwarn=0.
compute maxwarn=0.
do if ((desctmp(3,jd)=desctmp(4,jd)) and novar=0).
compute errcode(errs,1)=15.
compute errs=errs+1.
compute criterr=1.
compute novar=1.
end if.
compute tmp=((descdat(:,1)=desctmp(3,jd))+(descdat(:,1)=desctmp(4,jd))).
compute desctmp(8,jd)=(csum(tmp)=nrow(tmp)).
compute tmp = descdat.
compute tmp(GRADE(descdat),:) = descdat.
compute descdat = tmp.
release tmp.
compute decval={.16;.5;.84}.
loop kd=1 to 3.
compute low=trunc(decval(kd,1)*(nrow(descdat)+1)).
compute lowdec=decval(kd,1)*(nrow(descdat)+1)-low.
compute value=descdat(low,1)+(descdat((low+1),1)-descdat(low,1))*lowdec.
compute desctmp((4+kd),jd)=value.
end loop.
compute mnotev=1.
compute modvals=desctmp(5:7,:).
do if (quantile <> 1).
compute desctmp(5,jd)=desctmp(1,jd)-desctmp(2,jd).
compute desctmp(6,jd)=desctmp(1,jd).
compute desctmp(7,jd)=desctmp(1,jd)+desctmp(2,jd).
compute modvals=desctmp(5:7,:).
compute mnotev=2.
do if (modvals(1,1) < desctmp(3,1)).
compute modvals(1,1)=desctmp(3,1).
compute minwarn=1.
end if.
do if (modvals(3,1) > desctmp(4,1)).
compute modvals(3,1)=desctmp(4,1).
compute maxwarn=1.
end if.
end if.
do if (desctmp(8,1)=1).
compute modvals={desctmp(3,1);desctmp(4,1)}.
compute mnotev=0.
compute minwarn=0.
compute maxwarn=0.
end if.
end if.
end loop
.
compute xsd=desctmp(2,:).
compute xmodvals=modvals.
compute nxpval=nrow(xmodvals).
compute xprobval=xmodvals.
compute xdich=desctmp(8,1).
do if (model = 74 and xdich=1).
compute counterf=1.
end if.
do if (nms > 0).
compute mtmp=dat(:,(nys+nxs+1):(nys+nxs+nms)).

.
compute desctmp=make((8-(4* 0 )),ncol( mtmp ),-999).
loop jd=1 to ncol( mtmp ).
compute descdat= mtmp (:,jd).
compute desctmp(1,jd) = csum(descdat)/nrow(descdat).
compute desctmp(2,jd) = (nrow(descdat)*sscp(descdat))-(t(csum(descdat))*(csum(descdat))).
compute desctmp(2,jd) = sqrt(desctmp(2,jd)/(nrow(descdat)*(nrow(descdat)-1))).
compute desctmp(3,jd)=cmin(descdat).
compute desctmp(4,jd)=cmax(descdat).
do if ( 0 =0).
compute minwarn=0.
compute maxwarn=0.
do if ((desctmp(3,jd)=desctmp(4,jd)) and novar=0).
compute errcode(errs,1)=15.
compute errs=errs+1.
compute criterr=1.
compute novar=1.
end if.
compute tmp=((descdat(:,1)=desctmp(3,jd))+(descdat(:,1)=desctmp(4,jd))).
compute desctmp(8,jd)=(csum(tmp)=nrow(tmp)).
compute tmp = descdat.
compute tmp(GRADE(descdat),:) = descdat.
compute descdat = tmp.
release tmp.
compute decval={.16;.5;.84}.
loop kd=1 to 3.
compute low=trunc(decval(kd,1)*(nrow(descdat)+1)).
compute lowdec=decval(kd,1)*(nrow(descdat)+1)-low.
compute value=descdat(low,1)+(descdat((low+1),1)-descdat(low,1))*lowdec.
compute desctmp((4+kd),jd)=value.
end loop.
compute mnotev=1.
compute modvals=desctmp(5:7,:).
do if (quantile <> 1).
compute desctmp(5,jd)=desctmp(1,jd)-desctmp(2,jd).
compute desctmp(6,jd)=desctmp(1,jd).
compute desctmp(7,jd)=desctmp(1,jd)+desctmp(2,jd).
compute modvals=desctmp(5:7,:).
compute mnotev=2.
do if (modvals(1,1) < desctmp(3,1)).
compute modvals(1,1)=desctmp(3,1).
compute minwarn=1.
end if.
do if (modvals(3,1) > desctmp(4,1)).
compute modvals(3,1)=desctmp(4,1).
compute maxwarn=1.
end if.
end if.
do if (desctmp(8,1)=1).
compute modvals={desctmp(3,1);desctmp(4,1)}.
compute mnotev=0.
compute minwarn=0.
compute maxwarn=0.
end if.
end if.
end loop
.
do if ((rsum(desctmp(8,:))>0) and (mdichok <> 1)).
compute errcode(errs,1)=43.
compute errs=errs+1.
compute criterr=1.
end if.
compute mmodvals=modvals.
compute mprobval=mmodvals.
end if.
do if (nws > 0).
compute wtmp=dat(:,(nys+nxs+nms+1):(nys+nxs+nms+nws)).

.
compute desctmp=make((8-(4* 0 )),ncol( wtmp ),-999).
loop jd=1 to ncol( wtmp ).
compute descdat= wtmp (:,jd).
compute desctmp(1,jd) = csum(descdat)/nrow(descdat).
compute desctmp(2,jd) = (nrow(descdat)*sscp(descdat))-(t(csum(descdat))*(csum(descdat))).
compute desctmp(2,jd) = sqrt(desctmp(2,jd)/(nrow(descdat)*(nrow(descdat)-1))).
compute desctmp(3,jd)=cmin(descdat).
compute desctmp(4,jd)=cmax(descdat).
do if ( 0 =0).
compute minwarn=0.
compute maxwarn=0.
do if ((desctmp(3,jd)=desctmp(4,jd)) and novar=0).
compute errcode(errs,1)=15.
compute errs=errs+1.
compute criterr=1.
compute novar=1.
end if.
compute tmp=((descdat(:,1)=desctmp(3,jd))+(descdat(:,1)=desctmp(4,jd))).
compute desctmp(8,jd)=(csum(tmp)=nrow(tmp)).
compute tmp = descdat.
compute tmp(GRADE(descdat),:) = descdat.
compute descdat = tmp.
release tmp.
compute decval={.16;.5;.84}.
loop kd=1 to 3.
compute low=trunc(decval(kd,1)*(nrow(descdat)+1)).
compute lowdec=decval(kd,1)*(nrow(descdat)+1)-low.
compute value=descdat(low,1)+(descdat((low+1),1)-descdat(low,1))*lowdec.
compute desctmp((4+kd),jd)=value.
end loop.
compute mnotev=1.
compute modvals=desctmp(5:7,:).
do if (quantile <> 1).
compute desctmp(5,jd)=desctmp(1,jd)-desctmp(2,jd).
compute desctmp(6,jd)=desctmp(1,jd).
compute desctmp(7,jd)=desctmp(1,jd)+desctmp(2,jd).
compute modvals=desctmp(5:7,:).
compute mnotev=2.
do if (modvals(1,1) < desctmp(3,1)).
compute modvals(1,1)=desctmp(3,1).
compute minwarn=1.
end if.
do if (modvals(3,1) > desctmp(4,1)).
compute modvals(3,1)=desctmp(4,1).
compute maxwarn=1.
end if.
end if.
do if (desctmp(8,1)=1).
compute modvals={desctmp(3,1);desctmp(4,1)}.
compute mnotev=0.
compute minwarn=0.
compute maxwarn=0.
end if.
end if.
end loop
.
compute wmodvals=modvals.
compute wdich=desctmp(8,1).
compute wmin=desctmp(3,1).
compute wmax=desctmp(4,1).
compute minwwarn=minwarn.
compute maxwwarn=maxwarn.
compute wnotev=mnotev.
compute wmodval={ 999 }.
compute nwcontr=ncol(wmodval).
do if (wmodval(1,1) <> 999).
compute wmodvals=wmodval(1,1).
compute wmodcust=1.
do if (nwcontr > 1).
compute wmodvals=t(wmodval).
end if.
compute minwwarn=0.
compute maxwwarn=0.
compute wnotev=0.
end if.
compute wprobval=wmodvals.
compute nwpval=nrow(wmodvals).
end if.
do if (nzs > 0).
compute ztmp=dat(:,(nys+nxs+nms+nws+1):(nys+nxs+nms+nws+nzs)).

.
compute desctmp=make((8-(4* 0 )),ncol( ztmp ),-999).
loop jd=1 to ncol( ztmp ).
compute descdat= ztmp (:,jd).
compute desctmp(1,jd) = csum(descdat)/nrow(descdat).
compute desctmp(2,jd) = (nrow(descdat)*sscp(descdat))-(t(csum(descdat))*(csum(descdat))).
compute desctmp(2,jd) = sqrt(desctmp(2,jd)/(nrow(descdat)*(nrow(descdat)-1))).
compute desctmp(3,jd)=cmin(descdat).
compute desctmp(4,jd)=cmax(descdat).
do if ( 0 =0).
compute minwarn=0.
compute maxwarn=0.
do if ((desctmp(3,jd)=desctmp(4,jd)) and novar=0).
compute errcode(errs,1)=15.
compute errs=errs+1.
compute criterr=1.
compute novar=1.
end if.
compute tmp=((descdat(:,1)=desctmp(3,jd))+(descdat(:,1)=desctmp(4,jd))).
compute desctmp(8,jd)=(csum(tmp)=nrow(tmp)).
compute tmp = descdat.
compute tmp(GRADE(descdat),:) = descdat.
compute descdat = tmp.
release tmp.
compute decval={.16;.5;.84}.
loop kd=1 to 3.
compute low=trunc(decval(kd,1)*(nrow(descdat)+1)).
compute lowdec=decval(kd,1)*(nrow(descdat)+1)-low.
compute value=descdat(low,1)+(descdat((low+1),1)-descdat(low,1))*lowdec.
compute desctmp((4+kd),jd)=value.
end loop.
compute mnotev=1.
compute modvals=desctmp(5:7,:).
do if (quantile <> 1).
compute desctmp(5,jd)=desctmp(1,jd)-desctmp(2,jd).
compute desctmp(6,jd)=desctmp(1,jd).
compute desctmp(7,jd)=desctmp(1,jd)+desctmp(2,jd).
compute modvals=desctmp(5:7,:).
compute mnotev=2.
do if (modvals(1,1) < desctmp(3,1)).
compute modvals(1,1)=desctmp(3,1).
compute minwarn=1.
end if.
do if (modvals(3,1) > desctmp(4,1)).
compute modvals(3,1)=desctmp(4,1).
compute maxwarn=1.
end if.
end if.
do if (desctmp(8,1)=1).
compute modvals={desctmp(3,1);desctmp(4,1)}.
compute mnotev=0.
compute minwarn=0.
compute maxwarn=0.
end if.
end if.
end loop
.
compute zmodvals=modvals.
compute zdich=desctmp(8,1).
compute zmin=desctmp(3,1).
compute zmax=desctmp(4,1).
compute minzwarn=minwarn.
compute maxzwarn=maxwarn.
compute znotev=mnotev.
compute zmodval={ 999 }.
compute nzcontr=ncol(zmodval).
do if (zmodval(1,1) <> 999).
compute zmodvals=zmodval(1,1).
compute zmodcust=1.
do if (nzcontr > 1).
compute zmodvals=t(zmodval).
end if.
compute minzwarn=0.
compute maxzwarn=0.
compute znotev=0.
end if.
compute zprobval=zmodvals.
compute nzpval=nrow(zmodvals).
end if.
do if (ncs > 0).
compute ctmp=dat(:,(nys+nxs+nms+nws+nzs+1):(nys+nxs+nms+nws+nzs+ncs)).

.
compute desctmp=make((8-(4* 0 )),ncol( ctmp ),-999).
loop jd=1 to ncol( ctmp ).
compute descdat= ctmp (:,jd).
compute desctmp(1,jd) = csum(descdat)/nrow(descdat).
compute desctmp(2,jd) = (nrow(descdat)*sscp(descdat))-(t(csum(descdat))*(csum(descdat))).
compute desctmp(2,jd) = sqrt(desctmp(2,jd)/(nrow(descdat)*(nrow(descdat)-1))).
compute desctmp(3,jd)=cmin(descdat).
compute desctmp(4,jd)=cmax(descdat).
do if ( 0 =0).
compute minwarn=0.
compute maxwarn=0.
do if ((desctmp(3,jd)=desctmp(4,jd)) and novar=0).
compute errcode(errs,1)=15.
compute errs=errs+1.
compute criterr=1.
compute novar=1.
end if.
compute tmp=((descdat(:,1)=desctmp(3,jd))+(descdat(:,1)=desctmp(4,jd))).
compute desctmp(8,jd)=(csum(tmp)=nrow(tmp)).
compute tmp = descdat.
compute tmp(GRADE(descdat),:) = descdat.
compute descdat = tmp.
release tmp.
compute decval={.16;.5;.84}.
loop kd=1 to 3.
compute low=trunc(decval(kd,1)*(nrow(descdat)+1)).
compute lowdec=decval(kd,1)*(nrow(descdat)+1)-low.
compute value=descdat(low,1)+(descdat((low+1),1)-descdat(low,1))*lowdec.
compute desctmp((4+kd),jd)=value.
end loop.
compute mnotev=1.
compute modvals=desctmp(5:7,:).
do if (quantile <> 1).
compute desctmp(5,jd)=desctmp(1,jd)-desctmp(2,jd).
compute desctmp(6,jd)=desctmp(1,jd).
compute desctmp(7,jd)=desctmp(1,jd)+desctmp(2,jd).
compute modvals=desctmp(5:7,:).
compute mnotev=2.
do if (modvals(1,1) < desctmp(3,1)).
compute modvals(1,1)=desctmp(3,1).
compute minwarn=1.
end if.
do if (modvals(3,1) > desctmp(4,1)).
compute modvals(3,1)=desctmp(4,1).
compute maxwarn=1.
end if.
end if.
do if (desctmp(8,1)=1).
compute modvals={desctmp(3,1);desctmp(4,1)}.
compute mnotev=0.
compute minwarn=0.
compute maxwarn=0.
end if.
end if.
end loop
.
end if.
compute n=nrow(ytmp).
compute ones=make(n,1,1).
release dat.
do if (nws > 0 and mcw > 0).
compute tmp={rownum,wtmp(:,1)}.

.
compute dd= tmp.
compute temp = dd.
compute temp(GRADE(dd(:,2)),:) = dd.
compute dd = temp.
compute dummy = design(dd(:,2)).
compute nvls = ncol(dummy).
compute nnvls = csum(dummy).
compute mnvls = cmin(t(nnvls)).
compute conmat1=1.
do if (mnvls < 2).
compute errcode(errs,1) = 5.
compute errs = errs + 1.
compute criterr = 1.
end if.
do if (nvls > 9).
compute errcode(errs,1) = 4.
compute errs = errs+1.
compute criterr = 1.
end if.
do if (criterr = 0).
compute dumok = 1.
compute nnvls=make(nvls,1,0).
compute nnvls(1,1)=dd(1,2).
compute temp = 2.
loop i = 2 to n.
do if (dd(i,2) <> nnvls((temp-1),1)).
compute nnvls(temp,1)=dd(i,2).
compute temp = temp+1.
end if.
end loop.
do if ( mcw > 0).
compute x = dummy(:,2:ncol(dummy)).
compute nx = ncol(x).
compute minus1 = make(1,ncol(x),-1).
compute xdes=make((nx+1),3,0).
compute xdes(1,1)=dd(1,2).
compute xdes(1,2)=1.
compute temp = 2.
loop k = 2 to n.
do if (dd(k,2) <> dd((k-1),2)).
compute xdes(temp,2) = k.
compute xdes(temp,1) = dd(k,2).
compute xdes((temp-1),3) = k-1.
compute temp=temp+1.
end if.
end loop.
compute xdes((temp-1),3)=n.
compute xdes = {xdes, (xdes(:,3)-xdes(:,2)+1)}.
do if ( mcw = 4).
loop k = 1 to n.
do if (rsum(x(k,:)) = 0).
compute x(k,:) = minus1.
end if.
end loop.
end if.
do if ( mcw = 2 or mcw = 3 or mcw =5).
loop k = 1 to n.
do if (rsum(x(k,:)) > 0).
loop i = 1 to ncol(x).
do if (x(k,i) = 0).
compute x(k,i) = 1.
else.
break.
end if.
end loop.
end if.
end loop.
do if ( mcw = 3).
compute conmat1={-8,1,1,1,1,1,1,1,1; 0,-7,1,1,1,1,1,1,1; 0,0,-6,1,1,1,1,1,1; 0,0,0,-5,1,1,1,1,1; 0,0,0,0,-4,1,1,1,1; 0,0,0,0,0,-3,1,1,1; 0,0,0,0,0,0,-2,1,1; 0,0,0,0,0,0,0,-1,1}.
loop i = 1 to 8.
compute conmat1(i,:)=conmat1(i,:)/(10-i).
end loop.
compute conmat1=t(conmat1((10-nvls):8,(10-nvls):9)).
loop k=1 to n.
compute x(k,:)=conmat1((rsum(x(k,:))+1),:).
end loop.
end if.
end if.
do if ( mcw = 5).
compute custcode={ -999 }.
do if (ncol(custcode) <> (nvls*(nvls-1))).
compute errcode(errs,1) = (37+ 2 ).
compute errs = errs + 1.
compute criterr = 1.
end if.
do if (ncol(custcode) = (nvls*(nvls-1))).
compute conmat1=make(nvls,(nvls-1),0).
compute cnt=1.
loop i = 1 to nvls.
loop k = 1 to (nvls-1).
compute conmat1(i,k)=custcode(1,cnt).
compute cnt=cnt+1.
end loop.
end loop.
loop k=1 to n.
compute x(k,:)=conmat1((rsum(x(k,:))+1),:).
end loop.
end if.
end if.
compute xskip = 1.
compute dummat = make((nx+1),nx,0).
compute dummat((2:nrow(dummat)),:)=ident(nx).
do if ( mcw = 4).
compute dummat(1,:) = minus1.
end if.
do if ( mcw = 2).
loop i = 2 to nrow(dummat).
loop j = 1 to (i-1).
compute dummat(i,j) = 1.
end loop.
end loop.
end if.
do if ( mcw = 3).
compute dummat=conmat1.
end if.
do if ( mcw = 5 and criterr=0).
compute dummat=conmat1.
end if.
compute dummat={nnvls, dummat}.
compute x={dd(:,1),x}.
compute temp = x.
compute temp(GRADE(x(:,1)),:) = x.
compute x = temp.
release conmat1,temp,dd,xskip,xdes,dummy.
end if.
end if
.
compute wmodvals=nnvls.
compute nwpval=nrow(wmodvals).
do if (criterr=0).
compute minwwarn=0.
compute maxwwarn=0.
compute wnotev=0.
compute wtmp=x(:,2:ncol(x)).
compute wcatlab={'W1';'W2';'W3';'W4';'W5';'W6';'W7';'W8';'W9'}.
compute nwvls=nvls-1.
compute mcwok=1.
compute dummatw=dummat.
compute wprobval=dummatw(:,2:ncol(dummatw)).
do if (modcok=1).
compute wcontval=make(2,ncol(wprobval),-999).
compute temp=0.
loop i = 1 to 2.
loop j = 1 to nrow(dummatw).
do if (contvec(i,1)=dummatw(j,1)).
compute wcontval(i,:)=wprobval(j,:).
compute temp=temp+1.
end if.
end loop.
end loop.
do if (temp < 2).
compute notecode(notes,1) = 20.
compute notes = notes + 1.
compute modcok=0.
end if.
end if.
do if (wmodval(1,1) <> 999).
compute notecode(notes,1) = 9.
compute notes = notes + 1.
end if.
release tmp, dummat.
end if.
end if.
do if (nzs > 0 and mcz > 0).
compute tmp={rownum,ztmp(:,1)}.

.
compute dd= tmp.
compute temp = dd.
compute temp(GRADE(dd(:,2)),:) = dd.
compute dd = temp.
compute dummy = design(dd(:,2)).
compute nvls = ncol(dummy).
compute nnvls = csum(dummy).
compute mnvls = cmin(t(nnvls)).
compute conmat1=1.
do if (mnvls < 2).
compute errcode(errs,1) = 5.
compute errs = errs + 1.
compute criterr = 1.
end if.
do if (nvls > 9).
compute errcode(errs,1) = 4.
compute errs = errs+1.
compute criterr = 1.
end if.
do if (criterr = 0).
compute dumok = 1.
compute nnvls=make(nvls,1,0).
compute nnvls(1,1)=dd(1,2).
compute temp = 2.
loop i = 2 to n.
do if (dd(i,2) <> nnvls((temp-1),1)).
compute nnvls(temp,1)=dd(i,2).
compute temp = temp+1.
end if.
end loop.
do if ( mcz > 0).
compute x = dummy(:,2:ncol(dummy)).
compute nx = ncol(x).
compute minus1 = make(1,ncol(x),-1).
compute xdes=make((nx+1),3,0).
compute xdes(1,1)=dd(1,2).
compute xdes(1,2)=1.
compute temp = 2.
loop k = 2 to n.
do if (dd(k,2) <> dd((k-1),2)).
compute xdes(temp,2) = k.
compute xdes(temp,1) = dd(k,2).
compute xdes((temp-1),3) = k-1.
compute temp=temp+1.
end if.
end loop.
compute xdes((temp-1),3)=n.
compute xdes = {xdes, (xdes(:,3)-xdes(:,2)+1)}.
do if ( mcz = 4).
loop k = 1 to n.
do if (rsum(x(k,:)) = 0).
compute x(k,:) = minus1.
end if.
end loop.
end if.
do if ( mcz = 2 or mcz = 3 or mcz =5).
loop k = 1 to n.
do if (rsum(x(k,:)) > 0).
loop i = 1 to ncol(x).
do if (x(k,i) = 0).
compute x(k,i) = 1.
else.
break.
end if.
end loop.
end if.
end loop.
do if ( mcz = 3).
compute conmat1={-8,1,1,1,1,1,1,1,1; 0,-7,1,1,1,1,1,1,1; 0,0,-6,1,1,1,1,1,1; 0,0,0,-5,1,1,1,1,1; 0,0,0,0,-4,1,1,1,1; 0,0,0,0,0,-3,1,1,1; 0,0,0,0,0,0,-2,1,1; 0,0,0,0,0,0,0,-1,1}.
loop i = 1 to 8.
compute conmat1(i,:)=conmat1(i,:)/(10-i).
end loop.
compute conmat1=t(conmat1((10-nvls):8,(10-nvls):9)).
loop k=1 to n.
compute x(k,:)=conmat1((rsum(x(k,:))+1),:).
end loop.
end if.
end if.
do if ( mcz = 5).
compute custcode={ -999 }.
do if (ncol(custcode) <> (nvls*(nvls-1))).
compute errcode(errs,1) = (37+ 3 ).
compute errs = errs + 1.
compute criterr = 1.
end if.
do if (ncol(custcode) = (nvls*(nvls-1))).
compute conmat1=make(nvls,(nvls-1),0).
compute cnt=1.
loop i = 1 to nvls.
loop k = 1 to (nvls-1).
compute conmat1(i,k)=custcode(1,cnt).
compute cnt=cnt+1.
end loop.
end loop.
loop k=1 to n.
compute x(k,:)=conmat1((rsum(x(k,:))+1),:).
end loop.
end if.
end if.
compute xskip = 1.
compute dummat = make((nx+1),nx,0).
compute dummat((2:nrow(dummat)),:)=ident(nx).
do if ( mcz = 4).
compute dummat(1,:) = minus1.
end if.
do if ( mcz = 2).
loop i = 2 to nrow(dummat).
loop j = 1 to (i-1).
compute dummat(i,j) = 1.
end loop.
end loop.
end if.
do if ( mcz = 3).
compute dummat=conmat1.
end if.
do if ( mcz = 5 and criterr=0).
compute dummat=conmat1.
end if.
compute dummat={nnvls, dummat}.
compute x={dd(:,1),x}.
compute temp = x.
compute temp(GRADE(x(:,1)),:) = x.
compute x = temp.
release conmat1,temp,dd,xskip,xdes,dummy.
end if.
end if
.
compute zmodvals=nnvls.
compute nzpval=nrow(zmodvals).
do if (criterr=0).
compute minzwarn=0.
compute maxzwarn=0.
compute znotev=0.
compute ztmp=x(:,2:ncol(x)).
compute zcatlab={'Z1';'Z2';'Z3';'Z4';'Z5';'Z6';'Z7';'Z8';'Z9'}.
compute nzvls=nvls-1.
compute mczok=1.
compute dummatz=dummat.
compute zprobval=dummatz(:,2:ncol(dummatz)).
do if (modcok=1).
compute zcontval=make(2,ncol(zprobval),-999).
compute temp=0.
loop i = 1 to 2.
loop j = 1 to nrow(dummatz).
do if (contvec(i,2)=dummatz(j,1)).
compute zcontval(i,:)=zprobval(j,:).
compute temp=temp+1.
end if.
end loop.
end loop.
do if (temp < 2).
compute notecode(notes,1) = 20.
compute notes = notes + 1.
compute modcok=0.
end if.
end if.
do if (zmodval(1,1) <> 999).
compute notecode(notes,1) = 10.
compute notes = notes + 1.
end if.
release tmp, dummat.
end if.
end if.
do if (nxs > 0 and mcx > 0).
compute tmp={rownum,xtmp(:,1)}.

.
compute dd= tmp.
compute temp = dd.
compute temp(GRADE(dd(:,2)),:) = dd.
compute dd = temp.
compute dummy = design(dd(:,2)).
compute nvls = ncol(dummy).
compute nnvls = csum(dummy).
compute mnvls = cmin(t(nnvls)).
compute conmat1=1.
do if (mnvls < 2).
compute errcode(errs,1) = 5.
compute errs = errs + 1.
compute criterr = 1.
end if.
do if (nvls > 9).
compute errcode(errs,1) = 4.
compute errs = errs+1.
compute criterr = 1.
end if.
do if (criterr = 0).
compute dumok = 1.
compute nnvls=make(nvls,1,0).
compute nnvls(1,1)=dd(1,2).
compute temp = 2.
loop i = 2 to n.
do if (dd(i,2) <> nnvls((temp-1),1)).
compute nnvls(temp,1)=dd(i,2).
compute temp = temp+1.
end if.
end loop.
do if ( mcx > 0).
compute x = dummy(:,2:ncol(dummy)).
compute nx = ncol(x).
compute minus1 = make(1,ncol(x),-1).
compute xdes=make((nx+1),3,0).
compute xdes(1,1)=dd(1,2).
compute xdes(1,2)=1.
compute temp = 2.
loop k = 2 to n.
do if (dd(k,2) <> dd((k-1),2)).
compute xdes(temp,2) = k.
compute xdes(temp,1) = dd(k,2).
compute xdes((temp-1),3) = k-1.
compute temp=temp+1.
end if.
end loop.
compute xdes((temp-1),3)=n.
compute xdes = {xdes, (xdes(:,3)-xdes(:,2)+1)}.
do if ( mcx = 4).
loop k = 1 to n.
do if (rsum(x(k,:)) = 0).
compute x(k,:) = minus1.
end if.
end loop.
end if.
do if ( mcx = 2 or mcx = 3 or mcx =5).
loop k = 1 to n.
do if (rsum(x(k,:)) > 0).
loop i = 1 to ncol(x).
do if (x(k,i) = 0).
compute x(k,i) = 1.
else.
break.
end if.
end loop.
end if.
end loop.
do if ( mcx = 3).
compute conmat1={-8,1,1,1,1,1,1,1,1; 0,-7,1,1,1,1,1,1,1; 0,0,-6,1,1,1,1,1,1; 0,0,0,-5,1,1,1,1,1; 0,0,0,0,-4,1,1,1,1; 0,0,0,0,0,-3,1,1,1; 0,0,0,0,0,0,-2,1,1; 0,0,0,0,0,0,0,-1,1}.
loop i = 1 to 8.
compute conmat1(i,:)=conmat1(i,:)/(10-i).
end loop.
compute conmat1=t(conmat1((10-nvls):8,(10-nvls):9)).
loop k=1 to n.
compute x(k,:)=conmat1((rsum(x(k,:))+1),:).
end loop.
end if.
end if.
do if ( mcx = 5).
compute custcode={ -999 }.
do if (ncol(custcode) <> (nvls*(nvls-1))).
compute errcode(errs,1) = (37+ 1 ).
compute errs = errs + 1.
compute criterr = 1.
end if.
do if (ncol(custcode) = (nvls*(nvls-1))).
compute conmat1=make(nvls,(nvls-1),0).
compute cnt=1.
loop i = 1 to nvls.
loop k = 1 to (nvls-1).
compute conmat1(i,k)=custcode(1,cnt).
compute cnt=cnt+1.
end loop.
end loop.
loop k=1 to n.
compute x(k,:)=conmat1((rsum(x(k,:))+1),:).
end loop.
end if.
end if.
compute xskip = 1.
compute dummat = make((nx+1),nx,0).
compute dummat((2:nrow(dummat)),:)=ident(nx).
do if ( mcx = 4).
compute dummat(1,:) = minus1.
end if.
do if ( mcx = 2).
loop i = 2 to nrow(dummat).
loop j = 1 to (i-1).
compute dummat(i,j) = 1.
end loop.
end loop.
end if.
do if ( mcx = 3).
compute dummat=conmat1.
end if.
do if ( mcx = 5 and criterr=0).
compute dummat=conmat1.
end if.
compute dummat={nnvls, dummat}.
compute x={dd(:,1),x}.
compute temp = x.
compute temp(GRADE(x(:,1)),:) = x.
compute x = temp.
release conmat1,temp,dd,xskip,xdes,dummy.
end if.
end if
.
do if (criterr=0).
compute xtmp=x(:,2:ncol(x)).
compute xcatlab={'X1';'X2';'X3';'X4';'X5';'X6';'X7';'X8';'X9'}.
compute nxvls=nvls-1.
compute xdich=(nvls=2).
compute mcxok=1.
compute dummatx=dummat.
compute xmodvals=dummatx(:,1).
compute nxpval=nrow(xmodvals).
release tmp, dummat.
end if.
end if.
compute intlab=make(100,1,' ').


compute intlab( 1 ,1)= 'Int_1'.


compute intlab( 2 ,1)= 'Int_2'.


compute intlab( 3 ,1)= 'Int_3'.


compute intlab( 4 ,1)= 'Int_4'.


compute intlab( 5 ,1)= 'Int_5'.


compute intlab( 6 ,1)= 'Int_6'.


compute intlab( 7 ,1)= 'Int_7'.


compute intlab( 8 ,1)= 'Int_8'.


compute intlab( 9 ,1)= 'Int_9'.


compute intlab( 10 ,1)= 'Int_10'.


compute intlab( 11 ,1)= 'Int_11'.


compute intlab( 12 ,1)= 'Int_12'.


compute intlab( 13 ,1)= 'Int_13'.


compute intlab( 14 ,1)= 'Int_14'.


compute intlab( 15 ,1)= 'Int_15'.


compute intlab( 16 ,1)= 'Int_16'.


compute intlab( 17 ,1)= 'Int_17'.


compute intlab( 18 ,1)= 'Int_18'.


compute intlab( 19 ,1)= 'Int_19'.


compute intlab( 20 ,1)= 'Int_20'.


compute intlab( 21 ,1)= 'Int_21'.


compute intlab( 22 ,1)= 'Int_22'.


compute intlab( 23 ,1)= 'Int_23'.


compute intlab( 24 ,1)= 'Int_24'.


compute intlab( 25 ,1)= 'Int_25'.


compute intlab( 26 ,1)= 'Int_26'.


compute intlab( 27 ,1)= 'Int_27'.


compute intlab( 28 ,1)= 'Int_28'.


compute intlab( 29 ,1)= 'Int_29'.


compute intlab( 30 ,1)= 'Int_30'.


compute intlab( 31 ,1)= 'Int_31'.


compute intlab( 32 ,1)= 'Int_32'.


compute intlab( 33 ,1)= 'Int_33'.


compute intlab( 34 ,1)= 'Int_34'.


compute intlab( 35 ,1)= 'Int_35'.


compute intlab( 36 ,1)= 'Int_36'.


compute intlab( 37 ,1)= 'Int_37'.


compute intlab( 38 ,1)= 'Int_38'.


compute intlab( 39 ,1)= 'Int_39'.


compute intlab( 40 ,1)= 'Int_40'.


compute intlab( 41 ,1)= 'Int_41'.


compute intlab( 42 ,1)= 'Int_42'.


compute intlab( 43 ,1)= 'Int_43'.


compute intlab( 44 ,1)= 'Int_44'.


compute intlab( 45 ,1)= 'Int_45'.


compute intlab( 46 ,1)= 'Int_46'.


compute intlab( 47 ,1)= 'Int_47'.


compute intlab( 48 ,1)= 'Int_48'.


compute intlab( 49 ,1)= 'Int_49'.


compute intlab( 50 ,1)= 'Int_50'.


compute intlab( 51 ,1)= 'Int_51'.


compute intlab( 52 ,1)= 'Int_52'.


compute intlab( 53 ,1)= 'Int_53'.


compute intlab( 54 ,1)= 'Int_54'.


compute intlab( 55 ,1)= 'Int_55'.


compute intlab( 56 ,1)= 'Int_56'.


compute intlab( 57 ,1)= 'Int_57'.


compute intlab( 58 ,1)= 'Int_58'.


compute intlab( 59 ,1)= 'Int_59'.


compute intlab( 60 ,1)= 'Int_60'.


compute intlab( 61 ,1)= 'Int_61'.


compute intlab( 62 ,1)= 'Int_62'.


compute intlab( 63 ,1)= 'Int_63'.


compute intlab( 64 ,1)= 'Int_64'.


compute intlab( 65 ,1)= 'Int_65'.


compute intlab( 66 ,1)= 'Int_66'.


compute intlab( 67 ,1)= 'Int_67'.


compute intlab( 68 ,1)= 'Int_68'.


compute intlab( 69 ,1)= 'Int_69'.


compute intlab( 70 ,1)= 'Int_70'.


compute intlab( 71 ,1)= 'Int_71'.


compute intlab( 72 ,1)= 'Int_72'.


compute intlab( 73 ,1)= 'Int_73'.


compute intlab( 74 ,1)= 'Int_74'.


compute intlab( 75 ,1)= 'Int_75'.


compute intlab( 76 ,1)= 'Int_76'.


compute intlab( 77 ,1)= 'Int_77'.


compute intlab( 78 ,1)= 'Int_78'.


compute intlab( 79 ,1)= 'Int_79'.


compute intlab( 80 ,1)= 'Int_80'.


compute intlab( 81 ,1)= 'Int_81'.


compute intlab( 82 ,1)= 'Int_82'.


compute intlab( 83 ,1)= 'Int_83'.


compute intlab( 84 ,1)= 'Int_84'.


compute intlab( 85 ,1)= 'Int_85'.


compute intlab( 86 ,1)= 'Int_86'.


compute intlab( 87 ,1)= 'Int_87'.


compute intlab( 88 ,1)= 'Int_88'.


compute intlab( 89 ,1)= 'Int_89'.


compute intlab( 90 ,1)= 'Int_90'.


compute intlab( 91 ,1)= 'Int_91'.


compute intlab( 92 ,1)= 'Int_92'.


compute intlab( 93 ,1)= 'Int_93'.


compute intlab( 94 ,1)= 'Int_94'.


compute intlab( 95 ,1)= 'Int_95'.


compute intlab( 96 ,1)= 'Int_96'.


compute intlab( 97 ,1)= 'Int_97'.


compute intlab( 98 ,1)= 'Int_98'.


compute intlab( 99 ,1)= 'Int_99'.


compute intlab( 100 ,1)= 'Int_100'.

compute bcmat=make(needed,needed,0).
compute wcmat=make(needed,needed,0).
compute zcmat=make(needed,needed,0).
compute wzcmat=make(needed,needed,0).
compute wsum=0.
compute zsum=0.
compute wzsum=0.
end if.
do if (criterr = 0 and model <> 999).
compute modelmat= {1,0,0,0,0,0,0,1,0,0;2,0,0,0,0,0,0,1,1,0;3,0,0,0,0,0,0,1,1,1;4,0,0,0,0,0,0,0,0,0; 5,0,0,0,0,0,0,1,0,0;6,0,0,0,0,0,0,0,0,0;7,1,0,0,0,0,0,0,0,0;8,1,0,0,0,0,0,1,0,0; 9,1,1,0,0,0,0,0,0,0;10,1,1,0,0,0,0,1,1,0;11,1,1,1,0,0,0,0,0,0;12,1,1,1,0,0,0,1,1,1; 13,1,1,1,0,0,0,1,0,0;14,0,0,0,1,0,0,0,0,0;15,0,0,0,1,0,0,1,0,0;16,0,0,0,1,1,0,0,0,0; 17,0,0,0,1,1,0,1,1,0;18,0,0,0,1,1,1,0,0,0;19,0,0,0,1,1,1,1,1,1;20,0,0,0,1,1,1,1,0,0; 21,1,0,0,0,1,0,0,0,0;22,1,0,0,0,1,0,1,0,0;23,0,0,0,0,0,0,0,0,0;24,0,0,0,0,0,0,0,0,0; 25,0,0,0,0,0,0,0,0,0;26,0,0,0,0,0,0,0,0,0;27,0,0,0,0,0,0,0,0,0;28,1,0,0,0,1,0,0,1,0; 29,1,0,0,0,1,0,1,1,0;30,0,0,0,0,0,0,0,0,0;31,0,0,0,0,0,0,0,0,0;32,0,0,0,0,0,0,0,0,0; 33,0,0,0,0,0,0,0,0,0;34,0,0,0,0,0,0,0,0,0;35,0,0,0,0,0,0,0,0,0;36,0,0,0,0,0,0,0,0,0; 37,0,0,0,0,0,0,0,0,0;38,0,0,0,0,0,0,0,0,0;39,0,0,0,0,0,0,0,0,0;40,0,0,0,0,0,0,0,0,0; 41,0,0,0,0,0,0,0,0,0;42,0,0,0,0,0,0,0,0,0;43,0,0,0,0,0,0,0,0,0;44,0,0,0,0,0,0,0,0,0; 45,0,0,0,0,0,0,0,0,0;46,0,0,0,0,0,0,0,0,0;47,0,0,0,0,0,0,0,0,0;48,0,0,0,0,0,0,0

 ,0,0; 49,0,0,0,0,0,0,0,0,0;50,0,0,0,0,0,0,0,0,0;51,0,0,0,0,0,0,0,0,0;52,0,0,0,0,0,0,0,0,0; 53,0,0,0,0,0,0,0,0,0;54,0,0,0,0,0,0,0,0,0;55,0,0,0,0,0,0,0,0,0;56,0,0,0,0,0,0,0,0,0; 57,0,0,0,0,0,0,0,0,0;58,1,0,0,1,0,0,0,0,0;59,1,0,0,1,0,0,1,0,0;60,1,1,0,1,0,0,0,0,0; 61,1,1,0,1,0,0,1,0,0;62,1,1,0,1,0,0,0,1,0;63,1,1,0,1,0,0,1,1,0;64,1,0,0,1,1,0,0,0,0; 65,1,0,0,1,1,0,1,0,0;66,1,0,0,1,1,0,0,1,0;67,1,0,0,1,1,0,1,1,0;68,1,1,1,1,0,0,0,0,0; 69,1,1,1,1,0,0,1,1,1;70,1,0,0,1,1,1,0,0,0;71,1,0,0,1,1,1,1,1,1;72,1,1,1,1,1,1,0,0,0; 73,1,1,1,1,1,1,1,1,1;74,0,0,0,1,0,0,0,0,0;75,1,1,0,1,1,0,0,0,0;76,1,1,0,1,1,0,1,1,0; 77,0,0,0,0,0,0,0,0,0;78,0,0,0,0,0,0,0,0,0;79,0,0,0,0,0,0,0,0,0;80,0,0,0,0,0,0,0,0,0; 81,0,0,0,0,0,0,0,0,0;82,0,0,0,0,0,0,0,0,0;83,1,0,0,0,0,0,0,0,0;84,1,0,0,0,0,0,0,0,0; 85,1,0,0,0,0,0,1,0,0;86,1,0,0,0,0,0,1,0,0;87,0,0,0,1,0,0,0,0,0;88,0,0,0,1,0,0,0,0,0; 89,0,0,0,1,0,0,1,0,0;90,0,0,0,1,0,0,1,0,0;91,0,0,0,0,0,0,0,0,0;92,1,0,0,1,0,0,1,0,0}.
compute tmp=modelmat(model,2:ncol(modelmat)).
do if (model < 4).
compute bcmat((nxs+1),1)=1.
end if.
do if ((model > 3) and (model <> 6)).
compute bcmat((nxs+1):(nxs+nms),1)=onem.
compute bcmat(nrow(bcmat),(nxs+1):(nxs+nms))=t(onem).
compute bcmat(nrow(bcmat),1)=1.
end if.
do if ((model = 6) or (model > 82 and model < 93)).
loop j = 2 to nrow(bcmat).
loop i = 1 to (j-1).
compute bcmat(j,i)=1.
end loop.
end loop.
end if.
do if (model = 80).
loop i = 1 to nms.
compute bcmat((nrow(bcmat)-1),i)=1.
end loop.
end if.
do if (model = 81).
loop j = 3 to nrow(bcmat).
compute bcmat(j,2)=1.
end loop.
end if.
do if (model = 82).
compute bcmat(3,2)=1.
compute bcmat(5,4)=1.
end if.
do if (tmp(1,1)=1).
compute wcmat((nxs+1):(nxs+nms),1)=onem.
compute wprod=1.
compute xprod=1.
do if (model = 83 or model = 86).
compute onemsx=onem.
loop i = 1 to (nms-1).
compute onemsx(i+1,1)=0.
end loop.
compute wcmat((nxs+1):(nxs+nms),1)=onemsx.
end if.
end if.
do if (tmp(1,4)=1).
compute wcmat(nrow(wcmat),(nxs+1):(nxs+nms))=t(onem).
compute wprod=1.
do if (model = 87 or model = 90).
compute onemsx=onem.
loop i = 1 to (nms-1).
compute onemsx(i,1)=0.
end loop.
compute wcmat(nrow(wcmat),(nxs+1):(nxs+nms))=t(onemsx).
end if.
end if.
do if (tmp(1,7)=1).
compute wcmat(nrow(wcmat),1)=1.
compute wprod=1.
compute xprod=1.
end if.
do if (tmp(1,2)=1).
compute zcmat((nxs+1):(nxs+nms),1)=onem.
compute zprod=1.
compute xprod=1.
end if.
do if (tmp(1,5)=1).
compute zcmat(nrow(zcmat),(nxs+1):(nxs+nms))=t(onem).
compute zprod=1.
end if.
do if (tmp(1,8)=1).
compute zcmat(nrow(zcmat),1)=1.
compute zprod=1.
compute xprod=1.
end if.
do if (tmp(1,3)=1).
compute wzcmat((nxs+1):(nxs+nms),1)=onem.
compute xprod=1.
compute wprod=1.
compute zprod=1.
end if.
do if (tmp(1,6)=1).
compute wzcmat(nrow(wzcmat),(nxs+1):(nxs+nms))=t(onem).
compute zprod=1.
compute wprod=1.
end if.
do if (tmp(1,9)=1).
compute wzcmat(nrow(wzcmat),1)=1.
compute xprod=1.
compute wprod=1.
compute zprod=1.
end if.
do if (model = 91 or model = 92).
loop j = 1 to (nms-1).
loop i = 1 to j.
compute wcmat((nxs+1+j),(nxs+i))=1.
end loop.
end loop.
end if.
do if (nms < 0).
loop i = 1 to nms.
compute tmp=csum(wcmat(:,(1+i)))+csum(zcmat(:,(1+i)))+csum(wzcmat(:,(1+i))).
compute mprod(1,i)=(tmp>0).
end loop.
end if.
end if.
do if (ncs > 0).
compute ccmat=make((nms+nys),ncs,1).
do if (covmy=1).
compute ccmat(nrow(ccmat),:)=make(1,ncs,0).
end if.
do if (covmy=2).
compute ccmat(1:nms,:)=make(nms,ncs,0).
end if.
do if (cmatrix(1,1) <> -999).
do if (ncol(cmatrix) <> ((nms+nys)*ncs)).
compute errcode(errs,1)=29.
compute errs=errs+1.
compute criterr=1.
end if.
do if (criterr = 0).
compute tmp=1.
loop i = 1 to (nms+nys).
loop j = 1 to ncs.
compute ccmat(i,j)=1-(cmatrix(1,tmp) = 0).
compute tmp=tmp+1.
end loop.
end loop.
do if (rsum((csum(ccmat)=0)) <> 0).
compute errcode(errs,1)=30.
compute errs=errs+1.
compute criterr=1.
end if.
end if.
do if (covmy <> 0).
compute notecode(notes,1)=1.
compute notes=notes+1.
end if.
end if.
end if.
do if (criterr=0).
compute needed=needed*(needed-1)/2.
compute nopath=0.
do if (bmatrix(1,1) <> -999).
compute tmp=1.
do if ((ncol(bmatrix) <> needed) or (csum(rsum(bmatrix))=0)).
compute errcode(errs,1)=16.
compute errs=errs+1.
compute criterr=1.
else.
loop i = 2 to nrow(bcmat).
loop j = 1 to (i-1).
compute bcmat(i,j)=1-(bmatrix(1,tmp) = 0).
compute tmp=tmp+1.
end loop.
end loop.
end if.
do if ((csum(bcmat(:,1))=0) and criterr=0).
compute errcode(errs,1)=22.
compute errs=errs+1.
compute criterr=1.
end if.
do if ((rsum(bcmat(nrow(bcmat),:))=0) and criterr=0).
compute errcode(errs,1)=23.
compute errs=errs+1.
compute criterr=1.
end if.
compute dm=0.
do if (nms > 0).
loop i = 1 to nms.
do if (((rsum(bcmat((nxs+i),:)) = 0) or (csum(bcmat(:,(nxs+i))) = 0)) and (dm=0) and (criterr=0)).
compute errcode(errs,1)=26.
compute errs=errs+1.
compute criterr=1.
compute dm=1.
end if.
end loop.
end if.
release dm.
end if.
end if.
do if (criterr=0).
do if (wmatrix(1,1) <> -999).
compute tmp=1.
do if (ncol(wmatrix) <> needed).
compute errcode(errs,1)=17.
compute errs=errs+1.
compute criterr=1.
else.
compute modelvar(1,1)='CUSTOM'.
loop i = 2 to nrow(wcmat).
loop j = 1 to (i-1).
compute wcmat(i,j)=1-(wmatrix(1,tmp) = 0).
do if ((wcmat(i,j)=1) and (bcmat(i,j)=0) and (nopath=0)).
compute errcode(errs,1)=20.
compute errs=errs+1.
compute criterr=1.
compute nopath=1.
end if.
compute tmp=tmp+1.
end loop.
end loop.
end if.
end if.
do if (zmatrix(1,1) <> -999).
compute tmp=1.
do if (ncol(zmatrix) <> needed).
compute errcode(errs,1)=18.
compute errs=errs+1.
compute criterr=1.
else.
compute modelvar(1,1)='CUSTOM'.
do if (csum(rsum(wcmat))=0 and model=999).
compute errcode(errs,1)=21.
compute errs=errs+1.
compute criterr=1.
end if.
loop i = 2 to nrow(zcmat).
loop j = 1 to (i-1).
compute zcmat(i,j)=1-(zmatrix(1,tmp) = 0).
do if ((zcmat(i,j)=1) and (bcmat(i,j)=0) and (nopath=0)).
compute errcode(errs,1)=20.
compute errs=errs+1.
compute criterr=1.
compute nopath=1.
end if.
compute tmp=tmp+1.
end loop.
end loop.
end if.
end if.
compute tmp=1.
do if (wzmatrix(1,1) <> -999).
do if (ncol(wzmatrix) <> needed).
compute errcode(errs,1)=19.
compute errs=errs+1.
compute criterr=1.
end if.
compute modelvar(1,1)='CUSTOM'.
end if.
do if (criterr=0).
loop i = 2 to nrow(wzcmat).
loop j = 1 to (i-1).
do if (wzmatrix(1,1) <> -999).
compute wzcmat(i,j)=1-(wzmatrix(1,tmp) = 0).
end if.
do if (wzcmat(i,j)=1).
compute wcmat(i,j)=1.
compute zcmat(i,j)=1.
end if.
do if ((wzcmat(i,j)=1) and (bcmat(i,j)=0) and (nopath=0)).
compute errcode(errs,1)=20.
compute errs=errs+1.
compute criterr=1.
compute nopath=1.
end if.
compute tmp=tmp+1.
end loop.
end loop.
end if.
end if.
do if (criterr=0).
compute xprod=csum(wcmat(:,1))+csum(zcmat(:,1))+csum(wzcmat(:,1)).
compute xprod=(xprod > 0).
compute wsum=csum(rsum(wcmat)).
compute wprod=(wsum > 0).
do if (nms > 0).
loop i = 1 to nms.
compute tmp=csum(wcmat(:,(1+i)))+csum(zcmat(:,(1+i)))+csum(wzcmat(:,(1+i))).
compute mprod(1,i)=(tmp>0).
end loop.
end if.
do if ((wsum > 0) and (w = 'xxxxx')).
compute errcode(errs,1)=11.
compute errs=errs+1.
compute criterr=1.
end if.
do if ((wsum = 0) and (w <> 'xxxxx')).
compute errcode(errs,1)=10.
compute errs=errs+1.
compute criterr=1.
end if.
compute zsum=csum(rsum(zcmat)).
compute zprod=(zsum > 0).
do if ((zsum > 0) and (z = 'xxxxx')).
compute errcode(errs,1)=13.
compute errs=errs+1.
compute criterr=1.
end if.
do if ((zsum = 0) and (z <> 'xxxxx')).
compute errcode(errs,1)=12.
compute errs=errs+1.
compute criterr=1.
end if.
do if ((zsum > 0) and (wsum = 0)).
compute errcode(errs,1)=35.
compute errs=errs+1.
compute criterr=1.
end if.
end if.
do if (criterr=0 and nms > 1).
compute serchk=bcmat(2:(nrow(bcmat)-1),2:ncol(bcmat)).
do if (csum(rsum(serchk))) > 0.
compute serial=1.
do if (nms > 6).
compute errcode(errs,1)=36.
compute errs=errs+1.
compute criterr=1.
end if.
end if.
end if.
do if (center = 1 and criterr=0).
compute centvar={' '}.
do if (criterr=0).
do if (wprod=1 and mcwok=0 and nwpval > 0).
loop i = 1 to nws.
compute wtmp(:,i)=wtmp(:,i)-(csum(wtmp(:,i))/n).
compute centvar={centvar,wnames(1,i)}.
end loop.

.
compute desctmp=make((8-(4* wmodcust )),ncol( wtmp ),-999).
loop jd=1 to ncol( wtmp ).
compute descdat= wtmp (:,jd).
compute desctmp(1,jd) = csum(descdat)/nrow(descdat).
compute desctmp(2,jd) = (nrow(descdat)*sscp(descdat))-(t(csum(descdat))*(csum(descdat))).
compute desctmp(2,jd) = sqrt(desctmp(2,jd)/(nrow(descdat)*(nrow(descdat)-1))).
compute desctmp(3,jd)=cmin(descdat).
compute desctmp(4,jd)=cmax(descdat).
do if ( wmodcust =0).
compute minwarn=0.
compute maxwarn=0.
do if ((desctmp(3,jd)=desctmp(4,jd)) and novar=0).
compute errcode(errs,1)=15.
compute errs=errs+1.
compute criterr=1.
compute novar=1.
end if.
compute tmp=((descdat(:,1)=desctmp(3,jd))+(descdat(:,1)=desctmp(4,jd))).
compute desctmp(8,jd)=(csum(tmp)=nrow(tmp)).
compute tmp = descdat.
compute tmp(GRADE(descdat),:) = descdat.
compute descdat = tmp.
release tmp.
compute decval={.16;.5;.84}.
loop kd=1 to 3.
compute low=trunc(decval(kd,1)*(nrow(descdat)+1)).
compute lowdec=decval(kd,1)*(nrow(descdat)+1)-low.
compute value=descdat(low,1)+(descdat((low+1),1)-descdat(low,1))*lowdec.
compute desctmp((4+kd),jd)=value.
end loop.
compute mnotev=1.
compute modvals=desctmp(5:7,:).
do if (quantile <> 1).
compute desctmp(5,jd)=desctmp(1,jd)-desctmp(2,jd).
compute desctmp(6,jd)=desctmp(1,jd).
compute desctmp(7,jd)=desctmp(1,jd)+desctmp(2,jd).
compute modvals=desctmp(5:7,:).
compute mnotev=2.
do if (modvals(1,1) < desctmp(3,1)).
compute modvals(1,1)=desctmp(3,1).
compute minwarn=1.
end if.
do if (modvals(3,1) > desctmp(4,1)).
compute modvals(3,1)=desctmp(4,1).
compute maxwarn=1.
end if.
end if.
do if (desctmp(8,1)=1).
compute modvals={desctmp(3,1);desctmp(4,1)}.
compute mnotev=0.
compute minwarn=0.
compute maxwarn=0.
end if.
end if.
end loop
.
compute wmin=desctmp(3,1).
compute wmax=desctmp(4,1).
do if (wmodcust=0).
compute wmodvals=modvals.
compute wprobval=wmodvals.
end if.
end if.
do if (zprod=1 and mczok=0 and nzpval > 0).
loop i = 1 to nzs.
compute ztmp(:,i)=ztmp(:,i)-(csum(ztmp(:,i))/n).
compute centvar={centvar,znames(1,i)}.
end loop.

.
compute desctmp=make((8-(4* zmodcust )),ncol( ztmp ),-999).
loop jd=1 to ncol( ztmp ).
compute descdat= ztmp (:,jd).
compute desctmp(1,jd) = csum(descdat)/nrow(descdat).
compute desctmp(2,jd) = (nrow(descdat)*sscp(descdat))-(t(csum(descdat))*(csum(descdat))).
compute desctmp(2,jd) = sqrt(desctmp(2,jd)/(nrow(descdat)*(nrow(descdat)-1))).
compute desctmp(3,jd)=cmin(descdat).
compute desctmp(4,jd)=cmax(descdat).
do if ( zmodcust =0).
compute minwarn=0.
compute maxwarn=0.
do if ((desctmp(3,jd)=desctmp(4,jd)) and novar=0).
compute errcode(errs,1)=15.
compute errs=errs+1.
compute criterr=1.
compute novar=1.
end if.
compute tmp=((descdat(:,1)=desctmp(3,jd))+(descdat(:,1)=desctmp(4,jd))).
compute desctmp(8,jd)=(csum(tmp)=nrow(tmp)).
compute tmp = descdat.
compute tmp(GRADE(descdat),:) = descdat.
compute descdat = tmp.
release tmp.
compute decval={.16;.5;.84}.
loop kd=1 to 3.
compute low=trunc(decval(kd,1)*(nrow(descdat)+1)).
compute lowdec=decval(kd,1)*(nrow(descdat)+1)-low.
compute value=descdat(low,1)+(descdat((low+1),1)-descdat(low,1))*lowdec.
compute desctmp((4+kd),jd)=value.
end loop.
compute mnotev=1.
compute modvals=desctmp(5:7,:).
do if (quantile <> 1).
compute desctmp(5,jd)=desctmp(1,jd)-desctmp(2,jd).
compute desctmp(6,jd)=desctmp(1,jd).
compute desctmp(7,jd)=desctmp(1,jd)+desctmp(2,jd).
compute modvals=desctmp(5:7,:).
compute mnotev=2.
do if (modvals(1,1) < desctmp(3,1)).
compute modvals(1,1)=desctmp(3,1).
compute minwarn=1.
end if.
do if (modvals(3,1) > desctmp(4,1)).
compute modvals(3,1)=desctmp(4,1).
compute maxwarn=1.
end if.
end if.
do if (desctmp(8,1)=1).
compute modvals={desctmp(3,1);desctmp(4,1)}.
compute mnotev=0.
compute minwarn=0.
compute maxwarn=0.
end if.
end if.
end loop
.
compute zmin=desctmp(3,1).
compute zmax=desctmp(4,1).
do if (zmodcust=0).
compute zmodvals=modvals.
compute zprobval=zmodvals.
end if.
end if.
do if (xprod=1 and mcxok=0).
loop i = 1 to nxs.
compute xtmp(:,i)=xtmp(:,i)-(csum(xtmp(:,i))/n).
compute centvar={centvar,xnames(1,i)}.
end loop.

.
compute desctmp=make((8-(4* 0 )),ncol( xtmp ),-999).
loop jd=1 to ncol( xtmp ).
compute descdat= xtmp (:,jd).
compute desctmp(1,jd) = csum(descdat)/nrow(descdat).
compute desctmp(2,jd) = (nrow(descdat)*sscp(descdat))-(t(csum(descdat))*(csum(descdat))).
compute desctmp(2,jd) = sqrt(desctmp(2,jd)/(nrow(descdat)*(nrow(descdat)-1))).
compute desctmp(3,jd)=cmin(descdat).
compute desctmp(4,jd)=cmax(descdat).
do if ( 0 =0).
compute minwarn=0.
compute maxwarn=0.
do if ((desctmp(3,jd)=desctmp(4,jd)) and novar=0).
compute errcode(errs,1)=15.
compute errs=errs+1.
compute criterr=1.
compute novar=1.
end if.
compute tmp=((descdat(:,1)=desctmp(3,jd))+(descdat(:,1)=desctmp(4,jd))).
compute desctmp(8,jd)=(csum(tmp)=nrow(tmp)).
compute tmp = descdat.
compute tmp(GRADE(descdat),:) = descdat.
compute descdat = tmp.
release tmp.
compute decval={.16;.5;.84}.
loop kd=1 to 3.
compute low=trunc(decval(kd,1)*(nrow(descdat)+1)).
compute lowdec=decval(kd,1)*(nrow(descdat)+1)-low.
compute value=descdat(low,1)+(descdat((low+1),1)-descdat(low,1))*lowdec.
compute desctmp((4+kd),jd)=value.
end loop.
compute mnotev=1.
compute modvals=desctmp(5:7,:).
do if (quantile <> 1).
compute desctmp(5,jd)=desctmp(1,jd)-desctmp(2,jd).
compute desctmp(6,jd)=desctmp(1,jd).
compute desctmp(7,jd)=desctmp(1,jd)+desctmp(2,jd).
compute modvals=desctmp(5:7,:).
compute mnotev=2.
do if (modvals(1,1) < desctmp(3,1)).
compute modvals(1,1)=desctmp(3,1).
compute minwarn=1.
end if.
do if (modvals(3,1) > desctmp(4,1)).
compute modvals(3,1)=desctmp(4,1).
compute maxwarn=1.
end if.
end if.
do if (desctmp(8,1)=1).
compute modvals={desctmp(3,1);desctmp(4,1)}.
compute mnotev=0.
compute minwarn=0.
compute maxwarn=0.
end if.
end if.
end loop
.
compute xmodvals=modvals.
compute xprobval=xmodvals.
end if.
do if (nms > 0).
loop i = 1 to nms.
do if (mprod(1,i)=1).
compute mtmp(:,i)=mtmp(:,i)-(csum(mtmp(:,i))/n).
compute centvar={centvar,mnames(1,i)}.
end if.
end loop.

.
compute desctmp=make((8-(4* 0 )),ncol( mtmp ),-999).
loop jd=1 to ncol( mtmp ).
compute descdat= mtmp (:,jd).
compute desctmp(1,jd) = csum(descdat)/nrow(descdat).
compute desctmp(2,jd) = (nrow(descdat)*sscp(descdat))-(t(csum(descdat))*(csum(descdat))).
compute desctmp(2,jd) = sqrt(desctmp(2,jd)/(nrow(descdat)*(nrow(descdat)-1))).
compute desctmp(3,jd)=cmin(descdat).
compute desctmp(4,jd)=cmax(descdat).
do if ( 0 =0).
compute minwarn=0.
compute maxwarn=0.
do if ((desctmp(3,jd)=desctmp(4,jd)) and novar=0).
compute errcode(errs,1)=15.
compute errs=errs+1.
compute criterr=1.
compute novar=1.
end if.
compute tmp=((descdat(:,1)=desctmp(3,jd))+(descdat(:,1)=desctmp(4,jd))).
compute desctmp(8,jd)=(csum(tmp)=nrow(tmp)).
compute tmp = descdat.
compute tmp(GRADE(descdat),:) = descdat.
compute descdat = tmp.
release tmp.
compute decval={.16;.5;.84}.
loop kd=1 to 3.
compute low=trunc(decval(kd,1)*(nrow(descdat)+1)).
compute lowdec=decval(kd,1)*(nrow(descdat)+1)-low.
compute value=descdat(low,1)+(descdat((low+1),1)-descdat(low,1))*lowdec.
compute desctmp((4+kd),jd)=value.
end loop.
compute mnotev=1.
compute modvals=desctmp(5:7,:).
do if (quantile <> 1).
compute desctmp(5,jd)=desctmp(1,jd)-desctmp(2,jd).
compute desctmp(6,jd)=desctmp(1,jd).
compute desctmp(7,jd)=desctmp(1,jd)+desctmp(2,jd).
compute modvals=desctmp(5:7,:).
compute mnotev=2.
do if (modvals(1,1) < desctmp(3,1)).
compute modvals(1,1)=desctmp(3,1).
compute minwarn=1.
end if.
do if (modvals(3,1) > desctmp(4,1)).
compute modvals(3,1)=desctmp(4,1).
compute maxwarn=1.
end if.
end if.
do if (desctmp(8,1)=1).
compute modvals={desctmp(3,1);desctmp(4,1)}.
compute mnotev=0.
compute minwarn=0.
compute maxwarn=0.
end if.
end if.
end loop
.
compute mmodvals=modvals.
compute mprobval=mmodvals.
end if.
end if.
do if (ncol(centvar) > 1).
compute notecode(notes,1)=3.
compute notes=notes+1.
end if.
end if.
do if (criterr=0).
compute wsum=rsum(csum(wcmat)).
compute zsum=rsum(csum(zcmat)).
compute wzsum=rsum(csum(wzcmat)).
compute nump=make(1,(nys+nms),-999).
compue numint=make(1,(nys+nms),0).
compute datcount=1.
compute xtmpuse=0.
compute wtmpuse=0.
compute ztmpuse=0.
compute xwtmpus=0.
compute xztmpus=0.
compute wztmpus=0.
compute xwztmpu=0.
compute xtmploc=-999.
compute wtmploc=-999.
compute xwtmplo=-999.
compute ztmploc=-999.
compute xztmplo=-999.
compute wztmplo=-999.
compute xwztmplo=-999.
compute vlabs={' '}.
do if (ncs > 0).
compute ctmpuse=make(1,ncs,0).
end if.
do if (nms > 0).
compute mtmpuse=make(1,nms,0).
compute mwtmpus=make(1,nms,0).
compute mztmpus=make(1,nms,0).
compute mwztmpu=make(1,nms,0).
compute mtmploc=make(1,nms,0).
compute mwtmplo=make(nwvls,nms,-999).
compute mztmplo=make(nzvls,nms,-999).
compute mwztmplo=make((nwvls*nzvls),nms,-999).
end if.
do if (ncs > 0).
compute ctmploc=make(1,ncs,0).
end if.
compute fulldat=make(n,1,1).
compute datindx=make(1000,(nms+nys),-999).
compute wherew=make(2,(nms+nys),-999).
compute wherex=make(2,(nms+nys),-999).
compute wherez=make(2,(nms+nys),-999).
compute wherexw=make(2,(nms+nys),-999).
compute wherexz=make(2,(nms+nys),-999).
compute wherewz=make(2,(nms+nys),-999).
compute wherexwz=make(2,(nms+nys),-999).
do if (nms > 0).
compute wherem=make(nms,(nms+nys),-999).
compute wheremw = make(nms*2,(nms+nys),-999).
compute wheremz = make(nms*2,(nms+nys),-999).
compute wheremwz = make(nms*2,(nms+nys),-999).
end if.
compute wzhigh=make(1000,(((nms+1)*(nms+2))/2),0).
compute whigh=make(1000,(((nms+1)*(nms+2))/2),0).
compute zhigh=make(1000,(((nms+1)*(nms+2))/2),0).
compute fochigh=make(1000,(((nms+1)*(nms+2))/2),0).
compute xcoefloc={1;2;3;4;5;6;7;8;9}.
compute intkey = {' ', ' ', ' ', ' ', ' ', ' ', ' '}.
compute wzhighct=0.
compute whighct=0.
compute zhighct=0.
compute foccnt=0.
loop i = 2 to nrow(bcmat).
compute wdid=0.
compute zdid=0.
compute wzdid=0.
compute cntmp=1.
compute start=1.
do if (i < nrow(bcmat)).
compute outv=mtmp(:,(i-1)).
compute modlabel={mnames(1,(i-1));'constant'}.
end if.
do if (i = nrow(bcmat)).
compute outv=ytmp.
compute modlabel={ynames;'constant'}.
end if.
loop j = 1 to (i-1).
compute foccnt=foccnt+1.
do if (j = 1 and bcmat(i,j)=1).
compute outv={outv,xtmp}.
compute modlabel={modlabel;xcatlab(1:nxvls,1)}.
do if (xtmpuse=0).
compute fulldat={fulldat,xtmp}.
compute xtmpuse=1.
loop k4=datcount to (datcount+(nxvls-1)).
compute xtmploc={xtmploc;k4}.
end loop.
compute xtmploc=xtmploc(2:nrow(xtmploc),1).
compute datcount=datcount+nxvls.
end if.
compute datindx(start:(start+nrow(xtmploc)-1),(i-1))=xtmploc.
compute wherex(1,(i-1))=start+1.
compute wherex(2,(i-1))=start+nrow(xtmploc)-1+1.
compute onebl=make(nrow(xtmploc),1,1).
compute fochigh((start+1):(start+nrow(xtmploc)),foccnt)=onebl.
compute start=start+nrow(xtmploc).
end if.
do if (j > 1 and bcmat(i,j)=1).
compute outv={outv,mtmp(:,(j-1))}.
compute modlabel={modlabel;mnames(1,(j-1))}.
do if (mtmpuse(1,(j-1))=0).
compute fulldat={fulldat,mtmp(:,(j-1))}.
compute mtmpuse(1,(j-1))=1.
compute mtmploc(1,(j-1))=datcount.
compute datcount=datcount+1.
end if.
compute datindx(start:(start+nrow(mtmploc)-1),(i-1))=mtmploc(1,(j-1)).
compute wherem((j-1),(i-1))=start+1.
compute onebl=make(nrow(mtmploc(1,j-1)),1,1).
compute ttt=nrow(mtmploc(1,(j-1)))+start-1.
compute fochigh((start+1):(start+nrow(mtmploc(1,(j-1)))),foccnt)=onebl.
compute start=start+nrow(mtmploc(1,(j-1))).
end if.
end loop.
do if (wsum > 0).
loop j = 1 to (i-1).
compute whighct=whighct+1.
do if (j = 1 and wcmat(i,j)=1).
do if (wdid=0).
compute outv={outv,wtmp}.
compute modlabel={modlabel;wcatlab(1:nwvls,1)}.
compute wdid=1.
do if (wtmpuse=0).
compute fulldat={fulldat,wtmp}.
compute wtmpuse=1.
loop k4=datcount to (datcount+(nwvls-1)).
compute wtmploc={wtmploc;k4}.
end loop.
compute wtmploc=wtmploc(2:nrow(wtmploc),1).
compute datcount=datcount+nwvls.
end if.
end if.
compute datindx(start:(start+nrow(wtmploc)-1),(i-1))=wtmploc.
compute wherew(1,(i-1))=start+1.
compute wherew(2,(i-1))=start+nrow(wtmploc)-1+1.
compute start=start+nrow(wtmploc).
loop k1=1 to nxvls.
loop k2 = 1 to nwvls.
compute outv={outv,(xtmp(:,k1)&*wtmp(:,k2))}.
compute modlabel={modlabel;intlab(cntmp,1)}.
compute intkey={intkey;intlab(cntmp,1),':',xcatlab(k1,1),'x',wcatlab(k2,1),' ',' '}.
compute cntmp=cntmp+1.
end loop.
end loop.
do if (xwtmpus=0).
compute fulldat={fulldat,outv(:,(ncol(outv)-(nxvls*nwvls)+1):ncol(outv))}.
compute xwtmpus=1.
loop k4=datcount to (datcount+((nwvls*nxvls)-1)).
compute xwtmplo={xwtmplo;k4}.
end loop.
compute xwtmplo=xwtmplo(2:nrow(xwtmplo),1).
compute datcount=datcount+(nxvls*nwvls).
end if.
compute datindx(start:(start+nrow(xwtmplo)-1),(i-1))=xwtmplo.
compute wherexw(1,(i-1))=start+1.
compute wherexw(2,(i-1))=start+nrow(xwtmplo)-1+1.
compute onebl=make(nrow(xwtmplo),1,1).
compute whigh((start+1):(start+nrow(xwtmplo)),whighct)=onebl.
compute start=start+nrow(xwtmplo).
end if.
do if (j > 1 and wcmat(i,j)=1).
do if (wdid=0 and model <> 74).
compute outv={outv,wtmp}.
compute modlabel={modlabel;wcatlab(1:nwvls,1)}.
compute wdid=1.
do if (wtmpuse=0).
compute fulldat={fulldat,wtmp}.
compute wtmpuse=1.
loop k4=datcount to (datcount+(nwvls-1)).
compute wtmploc={wtmploc;k4}.
end loop.
compute wtmploc=wtmploc(2:nrow(wtmploc),1).
compute datcount=datcount+nwvls.
end if.
compute datindx(start:(start+nrow(wtmploc)-1),(i-1))=wtmploc.
compute wherew(1,(i-1))=start+1.
compute wherew(2,(i-1))=start+nrow(wtmploc)-1+1.
compute start=start+nrow(wtmploc).
end if.
loop k2 = 1 to nwvls.
compute outv={outv,(mtmp(:,(j-1))&*wtmp(:,k2))}.
compute modlabel={modlabel;intlab(cntmp,1)}.
compute intkey={intkey;intlab(cntmp,1),':', mnames(1,(j-1)),'x',wcatlab(k2,1),' ',' '}.
compute cntmp=cntmp+1.
end loop.
do if (mwtmpus(1,(j-1))=0).
compute fulldat={fulldat,outv(:,(ncol(outv)-nwvls+1):ncol(outv))}.
compute mwtmpus(1,(j-1))=1.
compute mw22=-999.
loop k4=datcount to (datcount+(nwvls-1)).
compute mw22={mw22;k4}.
end loop.
compute mwtmplo(:,(j-1))=mw22(2:nrow(mw22),1).
compute datcount=datcount+nwvls.
end if.
compute datindx(start:(start+nrow(mwtmplo)-1),(i-1))=mwtmplo(:,(j-1)).
compute wheremw(((2*j)-3),(i-1))=start+1.
compute wheremw(((2*j)-2),(i-1))=start+nrow(mwtmplo)-1+1.
compute onebl=make(nrow(mwtmplo),1,1).
compute whigh((start+1):(start+nrow(mwtmplo)),whighct)=onebl.
compute start=start+nrow(mwtmplo).
end if.
end loop.
end if.
do if (zsum > 0).
loop j = 1 to (i-1).
compute zhighct=zhighct+1.
do if (j = 1 and zcmat(i,j)=1).
do if (zdid=0).
compute outv={outv,ztmp}.
compute modlabel={modlabel;zcatlab(1:nzvls,1)}.
compute zdid=1.
do if (ztmpuse=0).
compute fulldat={fulldat,ztmp}.
compute ztmpuse=1.
loop k4=datcount to (datcount+(nzvls-1)).
compute ztmploc={ztmploc;k4}.
end loop.
compute ztmploc=ztmploc(2:nrow(ztmploc),1).
compute datcount=datcount+nzvls.
end if.
end if.
compute datindx(start:(start+nrow(ztmploc)-1),(i-1))=ztmploc.
compute wherez(1,(i-1))=start+1.
compute wherez(2,(i-1))=start+nrow(ztmploc)-1+1.
compute start=start+nrow(ztmploc).
loop k1=1 to nxvls.
loop k2 = 1 to nzvls.
compute outv={outv,(xtmp(:,k1)&*ztmp(:,k2))}.
compute modlabel={modlabel;intlab(cntmp,1)}.
compute intkey={intkey;intlab(cntmp,1),':',xcatlab(k1,1),'x',zcatlab(k2,1),' ',' '}.
compute cntmp=cntmp+1.
end loop.
end loop.
do if (xztmpus=0).
compute fulldat={fulldat,outv(:,(ncol(outv)-(nxvls*nzvls)+1):ncol(outv))}.
compute xztmpus=1.
loop k4=datcount to (datcount+((nzvls*nxvls)-1)).
compute xztmplo={xztmplo;k4}.
end loop.
compute xztmplo=xztmplo(2:nrow(xztmplo),1).
compute datcount=datcount+(nxvls*nzvls).
end if.
compute datindx(start:(start+nrow(xztmplo)-1),(i-1))=xztmplo.
compute wherexz(1,(i-1))=start+1.
compute wherexz(2,(i-1))=start+nrow(xztmplo)-1+1.
compute onebl=make(nrow(xztmplo),1,1).
compute zhigh((start+1):(start+nrow(xztmplo)),zhighct)=onebl.
compute start=start+nrow(xztmplo).
end if.
do if (j > 1 and zcmat(i,j)=1).
do if (zdid=0).
compute outv={outv,ztmp}.
compute modlabel={modlabel;zcatlab(1:nzvls,1)}.
compute zdid=1.
do if (ztmpuse=0).
compute fulldat={fulldat,ztmp}.
compute ztmpuse=1.
loop k4=datcount to (datcount+(nzvls-1)).
compute ztmploc={ztmploc;k4}.
end loop.
compute ztmploc=ztmploc(2:nrow(ztmploc),1).
compute datcount=datcount+nzvls.
end if.
compute datindx(start:(start+nrow(ztmploc)-1),(i-1))=ztmploc.
compute wherez(1,(i-1))=start+1.
compute wherez(2,(i-1))=start+nrow(ztmploc)-1+1.
compute start=start+nrow(ztmploc).
end if.
loop k2 = 1 to nzvls.
compute outv={outv,(mtmp(:,(j-1))&*ztmp(:,k2))}.
compute modlabel={modlabel;intlab(cntmp,1)}.
compute intkey={intkey;intlab(cntmp,1),':', mnames(1,(j-1)),'x',zcatlab(k2,1),' ',' '}.
compute cntmp=cntmp+1.
end loop.
do if (mztmpus(1,(j-1))=0).
compute fulldat={fulldat,outv(:,(ncol(outv)-nzvls+1):ncol(outv))}.
compute mztmpus(1,(j-1))=1.
compute mz22=-999.
loop k4=datcount to (datcount+(nzvls-1)).
compute mz22={mz22;k4}.
end loop.
compute mztmplo(:,(j-1))=mz22(2:nrow(mz22),1).
compute datcount=datcount+nzvls.
end if.
compute datindx(start:(start+nrow(mztmplo)-1),(i-1))=mztmplo(:,(j-1)).
compute wheremz(((2*j)-3),(i-1))=start+1.
compute wheremz(((2*j)-2),(i-1))=start+nrow(mztmplo)-1+1.
compute onebl=make(nrow(mztmplo),1,1).
compute zhigh((start+1):(start+nrow(mztmplo)),zhighct)=onebl.
compute start=start+nrow(mztmplo).
end if.
end loop.
end if.
do if (wzsum > 0).
loop j = 1 to (i-1).
compute wzhighct=wzhighct+1.
do if (j = 1 and wzcmat(i,j)=1).
do if (wzdid=0).
loop k1=1 to nwvls.
loop k2 = 1 to nzvls.
compute outv={outv,(wtmp(:,k1)&*ztmp(:,k2))}.
compute modlabel={modlabel;intlab(cntmp,1)}.
compute intkey={intkey;intlab(cntmp,1),':',wcatlab(k1,1),'x',zcatlab(k2,1),' ',' '}.
compute cntmp=cntmp+1.
end loop.
end loop.
do if (wztmpus=0).
compute fulldat={fulldat,outv(:,(ncol(outv)-(nwvls*nzvls)+1):ncol(outv))}.
compute wztmpus=1.
loop k4=datcount to (datcount+((nwvls*nzvls)-1)).
compute wztmplo={wztmplo;k4}.
end loop.
compute wztmplo=wztmplo(2:nrow(wztmplo),1).
compute datcount=datcount+(nzvls*nwvls).
end if.
compute wzdid=1.
end if.
compute datindx(start:(start+nrow(wztmplo)-1),(i-1))=wztmplo.
compute wherewz(1,(i-1))=start+1.
compute wherewz(2,(i-1))=start+nrow(wztmplo)-1+1.
compute start=start+nrow(wztmplo).
loop k1=1 to nxvls.
loop k2=1 to nwvls.
loop k3=1 to nzvls.
compute outv={outv,(xtmp(:,k1)&*wtmp(:,k2)&*ztmp(:,k3))}.
compute modlabel={modlabel;intlab(cntmp,1)}.
compute intkey={intkey;intlab(cntmp,1),':',xcatlab(k1,1),'x',wcatlab(k2,1),'x',zcatlab(k3,1)}.
compute cntmp=cntmp+1.
end loop.
end loop.
end loop.
do if (xwztmpu=0).
compute fulldat={fulldat,outv(:,(ncol(outv)-(nxvls*nwvls*nzvls)+1):ncol(outv))}.
compute xwztmpu=1.
loop k4=datcount to (datcount+((nzvls*nxvls*nwvls)-1)).
compute xwztmplo={xwztmplo;k4}.
end loop.
compute xwztmplo=xwztmplo(2:nrow(xwztmplo),1).
compute datcount=datcount+(nxvls*nzvls*nwvls).
end if.
compute datindx(start:(start+nrow(xwztmplo)-1),(i-1))=xwztmplo.
compute wherexwz(1,(i-1))=start+1.
compute wherexwz(2,(i-1))=start+nrow(xwztmplo)-1+1.
compute onebl=make(nrow(xwztmplo),1,1).
compute wzhigh((start+1):(start+nrow(xwztmplo)),wzhighct)=onebl.
compute start=start+nrow(xwztmplo).
end if.
do if (j > 1 and wzcmat(i,j)=1).
do if (wzdid=0).
loop k1=1 to nwvls.
loop k2 = 1 to nzvls.
compute outv={outv,(wtmp(:,k1)&*ztmp(:,k2))}.
compute modlabel={modlabel;intlab(cntmp,1)}.
compute intkey={intkey;intlab(cntmp,1),':',wcatlab(k1,1),'x',zcatlab(k2,1),' ',' '}.
compute cntmp=cntmp+1.
end loop.
end loop.
do if (wztmpus=0).
compute fulldat={fulldat,outv(:,(ncol(outv)-(nwvls*nzvls)+1):ncol(outv))}.
compute wztmpus=1.
loop k4=datcount to (datcount+((nwvls*nzvls)-1)).
compute wztmplo={wztmplo;k4}.
end loop.
compute wztmplo=wztmplo(2:nrow(wztmplo),1).
compute datcount=datcount+(nzvls*nwvls).
end if.
compute wzdid=1.
compute datindx(start:(start+nrow(wztmplo)-1),(i-1))=wztmplo.
compute wherewz(1,(i-1))=start+1.
compute wherewz(2,(i-1))=start+nrow(wztmplo)-1+1.
compute start=start+nrow(wztmplo).
end if.
loop k1 = 1 to nwvls.
loop k2 = 1 to nzvls.
compute outv={outv,(mtmp(:,(j-1))&*wtmp(:,k1)&*ztmp(:,k2))}.
compute modlabel={modlabel;intlab(cntmp,1)}.
compute intkey={intkey;intlab(cntmp,1),':',mnames(1,(j-1)),'x',wcatlab(k1,1),'x',zcatlab(k2,1)}.
compute cntmp=cntmp+1.
end loop.
end loop.
do if (mwztmpu(1,(j-1))=0).
compute fulldat={fulldat,outv(:,(ncol(outv)-(nwvls*nzvls)+1):ncol(outv))}.
compute mwztmpu(1,(j-1))=1.
compute mz22=-999.
loop k4=datcount to (datcount+(nwvls*nzvls)-1).
compute mz22={mz22;k4}.
end loop.
compute mwztmplo(:,(j-1))=mz22(2:nrow(mz22),1).
compute datcount=datcount+(nwvls*nzvls).
end if.
compute datindx(start:(start+nrow(mwztmplo)-1),(i-1))=mwztmplo(:,(j-1)).
compute wheremwz(((2*j)-3),(i-1))=start+1.
compute wheremwz(((2*j)-2),(i-1))=start+nrow(mwztmplo)-1+1.
compute onebl=make(nrow(mwztmplo),1,1).
compute wzhigh((start+1):(start+nrow(mwztmplo)),wzhighct)=onebl.
compute start=start+nrow(mwztmplo).
end if.
end loop.
end if.
do if (ncs > 0).
loop j = 1 to ncs.
do if (ccmat((i-1),j))=1.
compute outv={outv,ctmp(:,j)}.
compute modlabel={modlabel;covnames(1,j)}.
do if (ctmpuse(1,j)=0).
compute fulldat={fulldat,ctmp(:,j)}.
compute ctmpuse(1,j)=1.
compute ctmploc(1,j)=datcount.
compute datcount=datcount+1.
end if.
compute datindx(start:(start+nrow(ctmploc)-1),(i-1))=ctmploc(1,j).
compute start=start+nrow(ctmploc(1,j)).
end if.
end loop.
end if.
compute wdid=0.
compute zdid=0.
compute wzdid=0.
compute vlabs={vlabs;modlabel(2:nrow(modlabel),1)}.
compute numint(1,(i-1))=cntmp-1.
compute nump(1,(i-1))=nrow(modlabel)-1.
end loop.
release datcount, xtmpuse, wtmpuse, ztmpuse, xwtmpus, xztmpus, wztmpus, xwztmpu.
release xtmploc, wtmploc, xwtmplo, ztmploc, xztmplo, wztmplo, xwztmplo, foccnt.
do if (modcok=1 and ((nms > 0) or (zcmat(2,1) <> 1) or (mcx <> 0))).
compute notecode(notes,1) = 19.
compute notes = notes + 1.
compute modcok=0.
end if.
do if ((serial = 1 or (rsum(numint)>0) or nms=0) and mc > 0).
compute notecode(notes,1) = 15.
compute notes = notes + 1.
compute boot=mc.
compute mc=0.
end if.
do if (boot <> 0 or mc <> 0).
compute bootsz=boot.
do if (mc > 0).
compute bootsz=mc.
compute saveboot=0.
end if.
loop.
compute cilow = rnd(bootsz*(1-(conf/100))/2).
compute cihigh = trunc((bootsz*(conf/100)+(bootsz*(1-(conf/100))/2)))+1.
do if (cilow < 1 or cihigh > bootsz).
compute bootsz=trunc((bootsz+1000)/1000)*1000.
compute adjust = 1.
end if.
end loop if (cilow gt 0 and cihigh le bootsz).
do if (boot > 0).
compute boot=bootsz.
end if.
do if (mc > 0).
compute mc=bootsz.
end if.
do if (adjust = 1 and boot > 0).
compute notecode(notes,1) = 8.
compute notes = notes + 1.
end if.
do if (adjust = 1 and mc > 0).
compute notecode(notes,1) = 16.
compute notes = notes + 1.
end if.
end if.
compute maxboot = trunc(2*boot).
do if ( 0 > maxboot).
compute maxboot=trunc( 0 ).
end if.
do if (nms > 0).
release mtmpuse, mwtmpus, mwztmpu, mtmploc, mwtmplo, mztmplo, mwztmplo.
end if.
release wdid, zdid, wzdid, start,modlabel.
compute vlabs=vlabs(2:nrow(vlabs),1).
do if (rsum(numint) > 0).
compute intkey=intkey(2:nrow(intkey),:).
end if.
compute fulldat=fulldat(:,2:ncol(fulldat)).
compute fochigh=fochigh(1:rmax(nump),:).
compute whigh=whigh(1:rmax(nump),:).
compute zhigh=zhigh(1:rmax(nump),:).
compute wzhigh=wzhigh(1:rmax(nump),:).
compute coeffs=fochigh+whigh+zhigh+wzhigh.
compute bootloc=make(rmax(nump),ncol(nump),0).
do if (nms > 0).
compute cntmp=1.
loop i = 1 to ncol(nump).
loop j = 1 to nump(1,i).
compute bootloc(j,i)=cntmp.
compute cntmp=cntmp+1.
end loop.
end loop.
compute fochighb=make(nrow(fochigh),ncol(fochigh),0).
compute whighb=fochighb.
compute zhighb=fochighb.
compute wzhighb=fochighb.
compute thetaxmb=make(nrow(fochighb),nms,0).
compute thetaxyb=make(nrow(fochighb),1,0).
compute pathsfoc=make(nxvls,1,0).
compute cntmp=1.
loop i = 1 to (nms+nys).
loop j = 1 to i.
compute fochighb(:,cntmp)=fochigh(:,cntmp)&*bootloc(:,i).
compute whighb(:,cntmp)=whigh(:,cntmp)&*bootloc(:,i).
compute zhighb(:,cntmp)=zhigh(:,cntmp)&*bootloc(:,i).
compute wzhighb(:,cntmp)=wzhigh(:,cntmp)&*bootloc(:,i).
compute coeffsb=fochighb+whighb+zhighb+wzhighb.
do if ((i < (nms+nys)) and (j = 1)).
compute thetaxmb(:,i)=coeffsb(:,cntmp).
end if.
do if ((i = (nms+nys)) and (j = 1)).
compute thetaxyb(:,1)=coeffsb(:,cntmp).
end if.
compute cntmp=cntmp+1.
end loop.
end loop.
compute thetamyb=coeffsb(:,(ncol(coeffsb)-nms+1):ncol(coeffsb)).
do if (serial = 1).
compute thetammb=make(nrow(coeffsb),((nms*(nms-1))/2),0).
end if.
compute cntmp=1.
do if (nms > 1 and serial = 1).
loop i = 1 to (nms-1).
compute start=((i+2)*(i+1))/2.
loop j = 2 to (nms-i+1).
compute thetammb(:,cntmp)=coeffsb(:,start).
compute start=start+j+i-1.
compute cntmp=cntmp+1.
end loop.
end loop.
end if.
end if.
do if ((total = 1) and rsum(numint)=0).
compute dototal=1.
do if ((csum(bcmat(:,1)) <> (nms+nys)) or (rsum(bcmat(nrow(bcmat),:)) <> (nms+nys))).
compute dototal=0.
compute notecode(notes,1) = 12.
compute notes = notes + 1.
end if.
do if (ncs > 0).
do if ((csum(rsum(ccmat))) < (nrow(ccmat)*ncol(ccmat))).
compute dototal=0.
compute notecode(notes,1) = 11.
compute notes = notes + 1.
end if.
end if.
end if.
end if.
print/title = '**************** PROCESS Procedure for SPSS Version 3.00 *****************'.
print/title = '          Written by Andrew F. Hayes, Ph.D.       www.afhayes.com'.
print/title = '    Documentation available in Hayes (2018). www.guilford.com/p/hayes3'/space=0.
do if (criterr=0).
compute funny=1.
print modelvar/title = '**************************************************************************'/format = A8/rnames=modelvlb.
do if (ncs > 0).
print covnames/title='Covariates:'/format=A8.
end if.
print n/title='Sample'/rlabel='Size:'.
do if (( 'random' <> 'random')).
compute seedt= 'random'.
print seedt/title='Custom'/format=A12/rlabel = 'Seed:'.
end if.
do if (mcxok=1).
compute labtmp={xnames,t(xcatlab(1:nxvls,1))}.
print dummatx/title = 'Coding of categorical X variable for analysis:'/cnames = labtmp/format = F6.3.
end if.
do if (mcwok=1).
compute labtmp={wnames,t(wcatlab(1:nwvls,1))}.
print dummatw/title = 'Coding of categorical W variable for analysis:'/cnames = labtmp/format = F6.3.
end if.
do if (mczok=1).
compute labtmp={znames,t(zcatlab(1:nzvls,1))}.
print dummatz/title = 'Coding of categorical Z variable for analysis:'/cnames = labtmp/format = F6.3.
end if.
end if.
do if (criterr = 0).
compute outnames=ynames.
compute outvars=ytmp.
do if (nms > 0).
compute outnames={mnames,ynames}.
compute outvars={mtmp,ytmp}.
compute indcov=make(((nms*2)+(nms*(nxvls-1))),((nms*2)+(nms*(nxvls-1))),0).
compute mcsopath=make(((nms*2)+(nms*(nxvls-1))),1,0).
end if.
compute labstart=1.
compute intstart=1.
compute start=1.
compute coeffmat=make(1,6,0).
compute conseq={'        '}.
compute dfmat=0.
compute coeffcol=0.
compute pathscnt=1.
compute pathscn2=1.
loop i = 1 to (nms+nys).
print/title = '**************************************************************************'.
compute highf=make(1,5,0).
compute flabel={' '}.
compute y=outvars(:,i).
compute xindx=datindx(1:(nump(1,i)-1),i).
compute x = fulldat(:,xindx).
compute x={ones,x}.
compute xsq=t(x)*x.
compute exsq=eval(xsq).
release xsq.
compute zeroeig=csum(exsq <= 0.000000000002).
print outnames(1,i)/title = 'OUTCOME VARIABLE:'/format = A8/space=0.
do if (zeroeig > 0).
print / title = 'SINGULAR OR NEAR SINGULAR DATA MATRIX.'.
compute criterr=1.
compute errcode(errs,1)=31.
compute errs=errs+1.
end if.
compute means=csum(x)/n.
compute vlabsm=vlabs(labstart:(labstart+(nump(1,i)-1)),1).
do if (criterr=0).

.
do if ( 1 =1).
compute b = inv(t( x )* x )*t( x )* y.
compute modres=b.
do if ( 1 =1).
compute n1=nrow( x ).
compute dfres=n1-(ncol( x )).
compute sstotal = t( y -(csum( y )/n1))*( y -(csum( y )/n1)).
compute resid= y - x *b.
compute ssresid = csum((resid)&**2).
compute r2 = (sstotal-ssresid)/sstotal.
compute adjr2 = 1-((1-r2)*(n1-1)/(dfres)).
compute mse=ssresid/(n1-ncol( x )).

.
compute n1=nrow( x ).
compute invXtX = inv(t( x )* x ).
compute varb = mse *invXtX.
compute k3 = ncol( x ).
compute xhc=0.
do if ( hc <> 5).
compute xhc= x.
compute hat = xhc(:,1).
loop i3=1 to nrow(xhc).
compute hat(i3,1)= xhc(i3,:)*invXtX*t(xhc(i3,:)).
end loop.
do if ( hc = 0 or hc =1).
loop i3 = 1 to k3.
compute xhc(:,i3)=xhc(:,i3)&* resid.
end loop.
end if.
do if ( hc =3 or hc =2).
loop i3=1 to k3.
compute xhc(:,i3) = ( resid &/(1-hat)&**(1/(4- hc )))&*xhc(:,i3).
end loop.
end if.
do if ( hc = 4).
compute hcmn=make(n,2,4).
compute hcmn(:,2)=(n1*hat)/k3.
loop i3= 1 to k3.
compute xhc(:,i3) = ( resid &/(1-hat)&**(rmin(hcmn)/2))&*xhc(:,i3).
end loop.
end if.
compute varb=(invXtX*t(xhc)*xhc*invXtX).
do if ( hc =1).
compute varb=(n1/(n1-ncol( x )))&*varb.
end if.
end if.
compute hclab={'se(HC0)','se(HC1)','se(HC2)','se(HC3)','se(HC4)','se'}.
compute hclab=hclab(1,( hc +1)).
compute hcflab={'F(HC0)','F(HC1)','F(HC2)','F(HC3)','F(HC4)','F'}.
compute hcflab=hcflab(1,( hc +1)).
release xhc
.
compute seb=sqrt(diag(varb)).
compute trat = b&/seb.
compute p = 2*(1-tcdf(abs(trat), (dfres))).
compute tval = sqrt(dfres* (exp((dfres-(5/6))*((xp2/(dfres-(2/3)+(.11/dfres)))*(xp2/(dfres-(2/3)+(.11/dfres)))))-1)).
compute modres={modres,seb,trat,p}.
compute modres={modres,(b-tval&*seb),(b+tval&*seb)}.
compute modresl={'coeff',hclab,'t','p','LLCI','ULCI'}.
compute lmat = ident(ncol( x )).
compute lmat = lmat(:,2:ncol(lmat)).
compute fratio = (t(t(lmat)*b)*inv(t(lmat)*varb*lmat)*((t(lmat)*b)))/(ncol( x )-1).
compute pfr = 1-fcdf(fratio,(ncol( x )-1),dfres).
compute modsum={sqrt(r2),r2,mse,fratio,(ncol( x )-1),dfres,pfr}.
compute modsuml={'R','R-sq','MSE',hcflab,'df1','df2', 'p'}.
end if.
end if
.
compute obscoeff={obscoeff,t(b)}.
print modsum/title = 'Model Summary'/cnames = modsuml/format= F10.4.
print modres/title='Model'/rnames=vlabsm/cnames=modresl/format= F10.4.
compute coeffmat={coeffmat;modres}.
compute conseqt=make(nrow(modres),1,outnames(1,i)).
compute conseq={conseq;conseqt}.
compute dfmatt=make(nrow(modres),1,modsum(1,6)).
compute dfmat={dfmat;dfmatt}.
compute labstart=labstart+nump(1,i).
do if (nms > 0 and serial = 0 and (rsum(numint) = 0) and (normal=1 or mc > 0)).
do if (i < (nms+nys)).
compute indcov((((i-1)*nxvls)+1):(i*nxvls),(((i-1)*nxvls)+1):(i*nxvls))=varb(2:(1+nxvls),2:(1+nxvls)).
compute mcsopath((((i-1)*nxvls)+1):(i*nxvls) ,1)=modres(2:(1+nxvls),1).
end if.
do if (i = (nms+nys)).
compute atm=ncol(wherem).
compute indcov(((nms*nxvls)+1):nrow(mcsopath),((nms*nxvls)+1):nrow(mcsopath))=varb(wherem(1,atm):(wherem(1,atm)+nms-1),wherem(1,atm):(wherem(1,atm)+nms-1)).
compute mcsopath(((nms*nxvls)+1):nrow(mcsopath),1)=modres(wherem(1,atm):(wherem(1,atm)+nms-1),1).
compute sobelok=1.
end if.
end if.
do if ((i = (nms+nys)) and (bcmat(nrow(bcmat),1)=1)).
compute direff=modres(2:(1+nxvls),:).
compute direfflb=modresl.
compute direffl2=vlabsm(2:(1+nxvls),:).
compute lmat=make(nrow(b),1,0).
compute lmat2=make(nxvls,1,1).
compute lmat(2:(1+nxvls),1)=lmat2.

.
compute lmat2= lmat.
do if ( 0 =0).
compute lmat2 = mdiag( lmat ).
compute lmat3=make(nrow(lmat2),1,0).
loop flp=1 to ncol(lmat2).
do if (csum(lmat2(:,flp))=1).
compute lmat3={lmat3,lmat2(:,flp)}.
end if.
end loop.
compute lmat2=lmat3(:,2:ncol(lmat3)).
end if.
compute fratio = (t(t(lmat2)* b )*inv(t(lmat2)* varb *lmat2)*((t(lmat2)* b )))/ncol(lmat2).
compute pfr = 1-fcdf(fratio,ncol(lmat2),(n-nrow( b ))).
compute fresult={fratio,ncol(lmat2),(n-nrow( b )),pfr}.
do if ( 1 =1).
compute lmat3=1-rsum(lmat2).
compute xfm=make(n,csum(lmat3),0).
compute flpc=1.
loop flp=1 to nrow(lmat3).
do if (lmat3(flp,1)=1).
compute xfm(:,flpc)=x(:,flp).
compute flpc=flpc+1.
end if.
end loop.
compute bfm=inv(t(xfm)*xfm)*t(xfm)*y.
compute resid=y-(xfm*bfm).
compute sstotal=(y-(csum(y)/n)).
compute sstotal=csum(sstotal&*sstotal).
compute ssresid=csum(resid&*resid).
compute rsqch= r2 -((sstotal-ssresid)/sstotal).
compute fresult={rsqch,fresult}.
release xfm,flpc, resid, ssresid, bfm.
end if
.
compute diromni=fresult.
end if.
do if (numint(1,i) > 0).
compute intkeym=intkey(intstart:(intstart+numint(1,i)-1),:).
print intkeym/title='Product terms key:'/format = A8.
end if.
do if (covcoeff=1).
print varb/title='Covariance matrix of regression parameter estimates:'/rnames=vlabsm /cnames=vlabsm/format= F10.4.
end if.
do if (criterr = 0).
compute jj=0.
loop j = start to ((start+i)-1).
compute lmat=whigh(1:nump(1,i),j).
compute lmat2=wzhigh(1:nump(1,i),j).
do if ((csum(lmat) > 0) and (csum(lmat2) = 0)).

.
compute lmat2= lmat.
do if ( 0 =0).
compute lmat2 = mdiag( lmat ).
compute lmat3=make(nrow(lmat2),1,0).
loop flp=1 to ncol(lmat2).
do if (csum(lmat2(:,flp))=1).
compute lmat3={lmat3,lmat2(:,flp)}.
end if.
end loop.
compute lmat2=lmat3(:,2:ncol(lmat3)).
end if.
compute fratio = (t(t(lmat2)* b )*inv(t(lmat2)* varb *lmat2)*((t(lmat2)* b )))/ncol(lmat2).
compute pfr = 1-fcdf(fratio,ncol(lmat2),(n-nrow( b ))).
compute fresult={fratio,ncol(lmat2),(n-nrow( b )),pfr}.
do if ( 1 =1).
compute lmat3=1-rsum(lmat2).
compute xfm=make(n,csum(lmat3),0).
compute flpc=1.
loop flp=1 to nrow(lmat3).
do if (lmat3(flp,1)=1).
compute xfm(:,flpc)=x(:,flp).
compute flpc=flpc+1.
end if.
end loop.
compute bfm=inv(t(xfm)*xfm)*t(xfm)*y.
compute resid=y-(xfm*bfm).
compute sstotal=(y-(csum(y)/n)).
compute sstotal=csum(sstotal&*sstotal).
compute ssresid=csum(resid&*resid).
compute rsqch= r2 -((sstotal-ssresid)/sstotal).
compute fresult={rsqch,fresult}.
release xfm,flpc, resid, ssresid, bfm.
end if
.
compute highf={highf;fresult}.
do if (j = start).
compute flabel={flabel;'X*W'}.
end if.
do if (j > start).
do if (nms > 1).
compute flabel={flabel;highlbw(jj,1)}.
else if (nms = 1).
compute flabel={flabel;'M*W'}.
end if.
end if.
end if.
compute lmat=zhigh(1:nump(1,i),j).
compute lmat2=wzhigh(1:nump(1,i),j).
do if ((csum(lmat) > 0) and (csum(lmat2) = 0)).

.
compute lmat2= lmat.
do if ( 0 =0).
compute lmat2 = mdiag( lmat ).
compute lmat3=make(nrow(lmat2),1,0).
loop flp=1 to ncol(lmat2).
do if (csum(lmat2(:,flp))=1).
compute lmat3={lmat3,lmat2(:,flp)}.
end if.
end loop.
compute lmat2=lmat3(:,2:ncol(lmat3)).
end if.
compute fratio = (t(t(lmat2)* b )*inv(t(lmat2)* varb *lmat2)*((t(lmat2)* b )))/ncol(lmat2).
compute pfr = 1-fcdf(fratio,ncol(lmat2),(n-nrow( b ))).
compute fresult={fratio,ncol(lmat2),(n-nrow( b )),pfr}.
do if ( 1 =1).
compute lmat3=1-rsum(lmat2).
compute xfm=make(n,csum(lmat3),0).
compute flpc=1.
loop flp=1 to nrow(lmat3).
do if (lmat3(flp,1)=1).
compute xfm(:,flpc)=x(:,flp).
compute flpc=flpc+1.
end if.
end loop.
compute bfm=inv(t(xfm)*xfm)*t(xfm)*y.
compute resid=y-(xfm*bfm).
compute sstotal=(y-(csum(y)/n)).
compute sstotal=csum(sstotal&*sstotal).
compute ssresid=csum(resid&*resid).
compute rsqch= r2 -((sstotal-ssresid)/sstotal).
compute fresult={rsqch,fresult}.
release xfm,flpc, resid, ssresid, bfm.
end if
.
compute highf={highf;fresult}.
do if (j = start).
compute flabel={flabel;'X*Z'}.
end if.
do if (j > start).
do if (nms > 1).
compute flabel={flabel;highlbz(jj,1)}.
else if (nms = 1).
compute flabel={flabel;'M*Z'}.
end if.
end if.
end if.
compute lmat2=wzhigh(1:nump(1,i),j).
do if (csum(lmat2) > 0).

.
compute lmat2= lmat2.
do if ( 0 =0).
compute lmat2 = mdiag( lmat2 ).
compute lmat3=make(nrow(lmat2),1,0).
loop flp=1 to ncol(lmat2).
do if (csum(lmat2(:,flp))=1).
compute lmat3={lmat3,lmat2(:,flp)}.
end if.
end loop.
compute lmat2=lmat3(:,2:ncol(lmat3)).
end if.
compute fratio = (t(t(lmat2)* b )*inv(t(lmat2)* varb *lmat2)*((t(lmat2)* b )))/ncol(lmat2).
compute pfr = 1-fcdf(fratio,ncol(lmat2),(n-nrow( b ))).
compute fresult={fratio,ncol(lmat2),(n-nrow( b )),pfr}.
do if ( 1 =1).
compute lmat3=1-rsum(lmat2).
compute xfm=make(n,csum(lmat3),0).
compute flpc=1.
loop flp=1 to nrow(lmat3).
do if (lmat3(flp,1)=1).
compute xfm(:,flpc)=x(:,flp).
compute flpc=flpc+1.
end if.
end loop.
compute bfm=inv(t(xfm)*xfm)*t(xfm)*y.
compute resid=y-(xfm*bfm).
compute sstotal=(y-(csum(y)/n)).
compute sstotal=csum(sstotal&*sstotal).
compute ssresid=csum(resid&*resid).
compute rsqch= r2 -((sstotal-ssresid)/sstotal).
compute fresult={rsqch,fresult}.
release xfm,flpc, resid, ssresid, bfm.
end if
.
compute highf={highf;fresult}.
do if (j = start).
compute flabel={flabel;'X*W*Z'}.
end if.
do if (j > start).
do if (nms > 1).
compute flabel={flabel;highlbwz(jj,1)}.
else if (nms = 1).
compute flabel={flabel;'M*W*Z'}.
end if.
end if.
end if.
compute jj=jj+1.
end loop.
release jj.
compute start=start+i.
end if.
do if (nrow(highf) > 1).
compute highf=highf(2:nrow(highf),:).
compute flabel=flabel(2:nrow(flabel),1).
compute clabtmp={'R2-chng', hcflab,'df1','df2','p'}.
print highf/format = F10.4 /rnames=flabel/cnames=clabtmp/ title = 'Test(s) of highest order unconditional interaction(s):'.
compute intpb=highf(:,5).
end if.
compute intstart=intstart+numint(1,i).
end if.

.
do if (criterr=0).
compute threeway=0.
compute didprint=0.
compute didsome=0.
compute sigintct=0.
loop jmed =1 to (nms+1).
compute hasw=0.
compute hasz=0.
compute jnok=0.
compute nm1vls=0.
compute nm2vls=0.
compute panelgrp=0.
compute graphixs={'WITH', outnames(1,i), 'BY'}.
compute focpred4={' '}.
compute intprint=0.
compute modcat=0.
do if (jmed <= i).
do if ((jmed = 1) and ((i+1) = nrow(bcmat))).
compute pathscnt=pathscnt+1.
else.
compute paths={paths,bcmat((i+1),jmed)}.
compute pathsw={pathsw,wcmat((i+1),jmed)}.
compute pathsz={pathsz,zcmat((i+1),jmed)}.
compute pathswz={pathswz,wzcmat((i+1),jmed)}.
compute temp=fochigh(:,pathscnt)&*bootloc(:,i).
compute pathsfoc={pathsfoc,pathsfoc(:,1)}.
do if (jmed=1).
compute pathtype={pathtype,1}.
end if.
do if ((i+1)=nrow(bcmat)).
compute pathtype={pathtype,3}.
end if.
do if (jmed > 1) and ((i+1) < nrow(bcmat)).
compute pathtype={pathtype,2}.
end if.
do if (jmed=1 and nxvls > 1 and (bcmat((i+1),jmed)=1)).
compute pathsfoc(:,(pathscn2+1))=temp(2:(nxvls+1),1).
end if.
do if ((jmed > 1) or (jmed=1 and nxvls=1)).
compute temp=cmax(temp).
compute pathsfoc(1,(pathscn2+1))=temp.
end if.
compute pathscnt=pathscnt+1.
compute pathscn2=pathscn2+1.
do if (i <= nms).
compute pathsdv={pathsdv,mnames(1,i)}.
end if.
do if (i > nms).
compute pathsdv={pathsdv,ynames}.
end if.
end if.
compute coeffcol=coeffcol+1.
compute probettt=coeffs(1:nrow(b),coeffcol).
do if (jmed=1 and (bcmat((i+1),jmed)=1)).
compute omni=make(nrow(probettt),nxvls,0).
compute omnitmp=ident(nxvls).
compute omni(2:(1+nxvls),:)=omnitmp.
end if.
do if (csum(probettt)>0).
compute probvarb=make(csum(probettt),csum(probettt),999).
compute probcoef=make(csum(probettt),1,999).
compute coefflp2=1.
loop coefflp=1 to nrow(probettt).
do if (probettt(coefflp,1)=1).
compute probcoef(coefflp2,1)=b(coefflp,1).
compute coefflp2=coefflp2+1.
end if.
end loop.
compute coefflp=0.
compute coefflp2=0.
loop iclp=1 to nrow(probettt).
do if probettt(iclp,1)=1.
compute coefflp=coefflp+1.
compute coefflp2=coefflp.
compute probvarb(coefflp,coefflp) = varb(iclp,iclp).
do if (iclp < nrow(probettt)).
loop jclp=(iclp+1) to nrow(probettt).
do if (probettt(jclp,1)=1).
compute coefflp2=coefflp2+1.
compute probvarb(coefflp,coefflp2)=varb(iclp, jclp).
compute probvarb(coefflp2,coefflp)=varb(iclp, jclp).
end if.
end loop.
end if.
end if.
end loop.
end if.
end if.
compute xprobval=xmodvals.
do if (nxvls > 1).
compute xprobval=dummatx(:,2:ncol(dummatx)).
end if.
do if ((wcmat((i+1),jmed)=1) and (zcmat((i+1),jmed)=0)).
compute numplps=1.
compute modvals=wmodvals.
compute probeval=wmodvals.
compute wheremv1=wherexw.
compute nm1vls=nwvls.
compute lpstsp={1,1}.
compute modcat=0.
compute jnmod=wtmp.
compute jnmodlab=wnames.
compute jnok=1.
compute jnmin=wmin.
compute jnmax=wmax.
compute wherejn1=2.
do if (jmed=1).
compute wherejn3=wherexw(1,i).
do if (nxvls > 1).
compute jnok=0.
end if.
end if.
do if (jmed > 1).
compute wherejn1=wherem((jmed-1),i).
compute wherejn3=wheremw(((2*jmed)-3),i).
end if.
do if (nwvls > 1).
compute probeval=wprobval.
compute lpstsp(1,2)=ncol(probeval).
compute modcat=1.
compute jnok=0.
end if.
do if (wdich = 1).
compute modcat=1.
compute jnok=0.
end if.
compute problabs=wnames.
compute focpred3={wnames,'(W)'}.
compute hasw=1.
compute modgrph=wnames.
compute intprint=1.
compute sigintct=sigintct+1.
compute printpbe=intpb(sigintct,1).
end if.
do if ((wcmat((i+1),jmed)=0) and (zcmat((i+1),jmed)=1)).
compute numplps=1.
compute modvals=zmodvals.
compute probeval=zmodvals.
compute wheremv1=wherexz.
compute nm1vls=nzvls.
compute lpstsp={1,1}.
compute jnok=1.
compute jnmod=ztmp.
compute jnmin=zmin.
compute jnmax=zmax.
compute jnmodlab=znames.
compute wherejn1=2.
do if (jmed=1).
compute wherejn3=wherexz(1,i).
do if (nxvls > 1).
compute jnok=0.
end if.
end if.
do if (jmed > 1).
compute wherejn1=wherem((jmed-1),i).
compute wherejn3=wheremz(((2*jmed)-3),i).
end if.
do if (nzvls > 1).
compute probeval=zprobval.
compute lpstsp(1,2)=ncol(probeval).
compute modcat=1.
compute jnok=0.
end if.
do if (zdich = 1).
compute modcat=1.
compute jnok=0.
end if.
compute problabs=znames.
compute focpred3={znames,'(Z)'}.
compute modgrph=znames.
compute hasz=1.
compute intprint=1.
compute sigintct=sigintct+1.
compute printpbe=intpb(sigintct,1).
end if.
do if ((wzcmat((i+1),jmed)=1) or ((wcmat((i+1),jmed)=1) and (zcmat((i+1),jmed)=1))).
compute numplps=2.
compute probecnt=1.
compute intprint=1.
do if (wzcmat((i+1),jmed)=1).
compute sigintct=sigintct+1.
compute printpbe=intpb(sigintct,1).
else.
compute sigintct=sigintct+2.
compute printpbe=cmin(intpb((sigintct-1):sigintct,1)).
end if.
compute panelgrp=1.
compute hasw=1.
compute hasz=1.
compute panelcde={'/PANEL','ROWVAR=',znames,'.'}.
compute modgrph=wnames.
compute lpstsp={1,1;1,1}.
compute wheremv1=wherexw.
compute nm1vls=nwvls.
compute wheremv2=wherexz.
compute nm2vls=nzvls.
compute jnok=0.
do if (wzcmat((i+1),jmed)=1).
compute jnok=1.
end if.
do if (jmed > 1).
compute mprobval=mmodvals.
end if.
do if (jmed=1).
do if (nxvls > 1).
compute jnok=0.
end if.
end if.
do if (nwvls > 1).
compute lpstsp(1,2)=ncol(wprobval).
compute modcat=1.
compute jnok=0.
end if.
do if (zdich=1).
compute modcat=1.
compute jnok=0.
end if.
compute lpstsp(2,1)=lpstsp(1,2)+1.
compute lpstsp(2,2)=lpstsp(1,2)+1.
do if (nzvls > 1).
compute lpstsp(2,1)=lpstsp(1,2)+1.
compute lpstsp(2,2)=lpstsp(1,2)+ncol(zprobval).
compute jnok=0.
end if.
do if (zdich=1).
compute jnok=0.
end if.
compute omni3=make(nrow(b),(nxvls*nwvls),0).
do if (jmed > 1).
compute omni3=make(nrow(b),nwvls,0).
end if.
compute focpred3={wnames,'(W)'}.
compute focpred4={'    ', 'Mod var:', znames, '(Z)'}.
compute modvals=make((nrow(wmodvals)*nrow(zmodvals)),2,0).
compute probeval=make((nrow(wmodvals)*nrow(zmodvals)),(ncol(wprobval)+ncol(zprobval)),0).
loop probei= 1 to nrow(wmodvals).
loop probej =1 to nrow(zmodvals).
compute modvals(probecnt,1)=wmodvals(probei,1).
compute probeval(probecnt,1:nwvls)=wprobval(probei,:).
compute modvals(probecnt,2)=zmodvals(probej,1).
compute probeval(probecnt,(nwvls+1):(nwvls+nzvls))=zprobval(probej,:).
compute probecnt=probecnt+1.
end loop.
end loop.
do if (wzcmat((i+1),jmed)=1).
compute numplps=numplps+1.
compute probprod=make(1,(ncol(wprobval)*ncol(zprobval)),0).
compute lpstsp2={1,1}.
compute lpstsp={lpstsp;lpstsp2}.
compute lpstsp(3,1)=lpstsp(2,2)+1.
compute lpstsp(3,2)=lpstsp(2,2)+ncol(probprod).
compute jnmod=ztmp.
compute jnmin=zmin.
compute jnmax=zmax.
compute jnmodlab=znames.
do if (jmed = 1).
compute wherejn1=wherexw(1,i).
compute wherejn3=wherexwz(1,i).
end if.
do if (jmed > 1).
compute wherejn1=wheremw(((2*jmed)-3),i).
compute wherejn3=wheremwz(((2*jmed)-3),i).
end if.
loop probei = 1 to nrow(wmodvals).
loop probej = 1 to nrow(zmodvals).
compute probtemp=1.
loop probek = 1 to ncol(wprobval).
compute probtemp={probtemp,(wprobval(probei,probek)&*zprobval(probej,:))}.
end loop.
compute probprod={probprod;probtemp(1,2:ncol(probtemp))}.
end loop.
end loop.
compute probprod=probprod(2:nrow(probprod),:).
compute probeval={probeval,probprod}.
end if.
compute problabs={wnames,znames}.
release probecnt, probei, probej.
end if.
do if (intprint=1).
compute focpred={'   Focal', 'predict:'}.
do if (jmed=1).
compute focpred={focpred,xnames,'(X)'}.
compute focplotv=xmodvals.
end if.
do if (jmed >1).
do if (nms > 1).
compute focpred={focpred,mnames(1,(jmed-1)), medlb2(1,(jmed-1))}.
end if.
do if (nms = 1).
compute focpred={focpred,mnames(1,(jmed-1)), '(M)'}.
end if.
compute focplotv=mmodvals(:,(jmed-1)).
end if.
compute focpred2={'    ', 'Mod var:',focpred3}.
compute focpred={focpred;focpred2}.
do if (ncol(focpred4) > 1).
compute focpred={focpred;focpred4}.
compute focpred4={' '}.
end if.
release focpred2,focpred3.
do if ((plot = 1 or plot = 2) or (printpbe <= intprobe)).
print focpred/title='----------'/format=A8/space=0.
end if.
compute foctmp=make(nrow(modvals),1,1).
compute probexpl=1.
compute probeva2={foctmp,probeval}.
do if (jmed=1 and nxs > 0 and mcx > 0).
compute probexpl=nxvls.
end if.
compute foctmp=make(nrow(modvals),1,1).
compute modvals3=make(1,(6+ncol(problabs)),0).
compute probrown=make(nrow(probeval),1,0).
compute jtmp=1.
loop probei = 1 to nrow(probeval).
compute probrown(probei,1)=jtmp.
compute jtmp=jtmp+nxvls.
end loop.
release jtmp.
compute probrow=999.
compute modvarl=problabs.
do if (plot = 1 or plot = 2 or nxvls > 1).
compute plotvals=make((nrow(modvals)*nrow(focplotv)),(ncol(modvals)+1),999).
loop ploti=1 to nrow(modvals).
loop plotj=1 to nrow(focplotv).
compute plotvals((((ploti-1)*nrow(focplotv))+plotj),2:ncol(plotvals))=modvals(ploti,:).
compute plotvals((((ploti-1)*nrow(focplotv))+plotj),1)=focplotv(plotj,1).
end loop.
end loop.
compute focpredn=3.
do if (jmed=1).
do if (nxvls > 1).
compute focpredn=(nxvls+1).
end if.
do if (nxvls=1 and xdich=1).
compute focpredn=2.
end if.
end if.
compute meanmat=mdiag(means).
compute onesmat=make(nrow(meanmat),(nrow(probeval)*focpredn),1).
compute probeplt=t(mdiag(means)*onesmat).
do if (jmed=1).
do if ((wcmat((i+1),1)=1) or (zcmat((i+1),1)=1)).
compute plotcnt=1.
compute iloops=nwpval*nzpval.
compute plotmx=nxpval*nzpval.
do if ((wcmat((i+1),1)=1) and (zcmat((i+1),1)=0)).
compute iloops=nwpval.
compute plotmx=nxpval.
end if.
do if ((wcmat((i+1),1)=0) and (zcmat((i+1),1)=1)).
compute iloops=nzpval.
compute plotmx=nxpval.
end if.
compute xestvals=make((nxpval*iloops),ncol(xprobval),-999).
do if (wcmat((i+1),1)=1).
compute westvals=make(nrow(xestvals),ncol(wprobval),-999).
end if.
do if (zcmat((i+1),1)=1).
compute zestvals=make(nrow(xestvals),ncol(zprobval),-999).
end if.
loop ploti=1 to iloops.
loop plotj=1 to nxpval.
compute xestvals(plotcnt,:)=xprobval(plotj,:).
compute plotcnt=plotcnt+1.
end loop.
end loop.
compute plotcnt=1.
compute plotcnt1=1.
compute plotcnt2=1.
compute plotcntz=1.
loop ploti = 1 to (iloops*nxpval).
do if (wcmat((i+1),1)=1).
compute westvals(ploti,:)=wprobval(plotcnt1,:).
end if.
do if ((wcmat((i+1),1)=0) and (zcmat((i+1),1)=1)).
compute zestvals(ploti,:)=zprobval(plotcnt1,:).
end if.
do if ((wcmat((i+1),1)=1) and (zcmat((i+1),1)=1)).
compute zestvals(ploti,:)=zprobval(plotcnt2,:).
compute plotcntz=plotcntz+1.
end if.
compute plotcnt=plotcnt+1.
do if (plotcnt > plotmx).
compute plotcnt=1.
compute plotcnt1=plotcnt1+1.
end if.
do if (plotcntz > nxpval).
compute plotcnt2=plotcnt2+1.
compute plotcntz=1.
do if (plotcnt2 > nzpval).
compute plotcnt2=1.
end if.
end if.
end loop.
compute probeplt(:,2:(1+(ncol(xestvals))))=xestvals.
do if (wcmat((i+1),1)=1).
compute probeplt(:,(wherew(1,i)):(wherew(2,i)))=westvals.
end if.
do if (zcmat((i+1),1)=1).
compute probeplt(:,(wherez(1,i)):(wherez(2,i)))=zestvals.
end if.
end if.
end if.
do if (jmed > 1).
do if ((wcmat((i+1),jmed)=1) or (zcmat((i+1),jmed)=1)).
compute plotcnt=1.
compute iloops=nwpval*nzpval.
compute plotmx=3*nzpval.
do if ((wcmat((i+1),jmed)=1) and (zcmat((i+1),jmed)=0)).
compute iloops=nwpval.
compute plotmx=3.
end if.
do if ((wcmat((i+1),jmed)=0) and (zcmat((i+1),jmed)=1)).
compute iloops=nzpval.
compute plotmx=3.
end if.
compute mestvals=make((3*iloops),1,-999).
do if (wcmat((i+1),jmed)=1).
compute westvals=make(nrow(mestvals),ncol(wprobval),-999).
end if.
do if (zcmat((i+1),jmed)=1).
compute zestvals=make(nrow(mestvals),ncol(zprobval),-999).
end if.
loop ploti=1 to iloops.
loop plotj=1 to 3.
compute mestvals(plotcnt,:)=mprobval(plotj,(jmed-1)).
compute plotcnt=plotcnt+1.
end loop.
end loop.
compute plotcnt=1.
compute plotcnt1=1.
compute plotcnt2=1.
compute plotcntz=1.
loop ploti = 1 to (iloops*3).
do if (wcmat((i+1),jmed)=1).
compute westvals(ploti,:)=wprobval(plotcnt1,:).
end if.
do if ((wcmat((i+1),jmed)=0) and (zcmat((i+1),jmed)=1)).
compute zestvals(ploti,:)=zprobval(plotcnt1,:).
end if.
do if ((wcmat((i+1),jmed)=1) and (zcmat((i+1),jmed)=1)).
compute zestvals(ploti,:)=zprobval(plotcnt2,:).
compute plotcntz=plotcntz+1.
end if.
compute plotcnt=plotcnt+1.
do if (plotcnt > plotmx).
compute plotcnt=1.
compute plotcnt1=plotcnt1+1.
end if.
do if (plotcntz > 3).
compute plotcnt2=plotcnt2+1.
compute plotcntz=1.
do if (plotcnt2 > nzpval).
compute plotcnt2=1.
end if.
end if.
end loop.
compute probeplt(:,wherem((jmed-1),i))=mestvals.
do if (wcmat((i+1),jmed)=1).
compute probeplt(:,(wherew(1,i)):(wherew(2,i)))=westvals.
end if.
do if (zcmat((i+1),jmed)=1).
compute probeplt(:,(wherez(1,i)):(wherez(2,i)))=zestvals.
end if.
end if.
end if.
compute prodloop = 1.
do if (jmed=1).
compute prodloop=ncol(xestvals).
end if.
do if (wcmat((i+1),jmed))=1.
compute plotcnt=0.
loop ploti = 1 to prodloop.
loop plotj = 1 to ncol(westvals).
do if (jmed=1).
compute probeplt(:,(wherexw(1,i)+plotcnt))=xestvals(:,ploti)&*westvals(:,plotj).
end if.
do if (jmed > 1).
compute probeplt(:,(wheremw(((jmed*2)-3) ,i)+plotcnt))=mestvals(:,ploti)&*westvals(:,plotj).
end if.
compute plotcnt=plotcnt+1.
end loop.
end loop.
end if.
do if (zcmat((i+1),jmed))=1.
compute plotcnt=0.
loop ploti = 1 to prodloop.
loop plotj = 1 to ncol(zestvals).
do if (jmed = 1).
compute probeplt(:,(wherexz(1,i)+plotcnt))=xestvals(:,ploti)&*zestvals(:,plotj).
end if.
do if (jmed > 1).
compute probeplt(:,(wheremz(((jmed*2)-3),i)+plotcnt))=mestvals(:,ploti)&*zestvals(:,plotj).
end if.
compute plotcnt=plotcnt+1.
end loop.
end loop.
end if.
do if (wzcmat((i+1),jmed))=1.
compute plotcnt=0.
compute threeway=1.
loop ploti = 1 to ncol(westvals).
loop plotj = 1 to ncol(zestvals).
compute probeplt(:,(wherewz(1,i)+plotcnt))=westvals(:,ploti)&*zestvals(:,plotj).
compute plotcnt=plotcnt+1.
end loop.
end loop.
compute plotcnt=0.
loop plotk = 1 to prodloop.
loop ploti = 1 to ncol(westvals).
loop plotj = 1 to ncol(zestvals).
do if (jmed = 1).
compute probeplt(:,(wherexwz(1,i)+plotcnt))=xestvals(:,plotk)&*westvals(:,ploti)&*zestvals(:,plotj).
end if.
do if (jmed > 1).
compute probeplt(:,(wheremwz(((jmed*2)-3),i)+plotcnt))=mestvals(:,plotk)&*westvals(:,ploti)&*zestvals(:,plotj).
end if.
compute plotcnt=plotcnt+1.
end loop.
end loop.
end loop.
end if.
loop newplp=1 to i.
do if (newplp <> jmed).
do if (wcmat((i+1),newplp))=1.
compute prodloop=1.
do if (newplp=1).
compute prodloop=nxvls.
end if.
compute plotcnt=0.
loop ploti = 1 to prodloop.
loop plotj = 1 to nwvls.
do if (newplp = 1).
compute probeplt(:,(wherexw(1,i)+plotcnt))=probeplt(:,(1+ploti))&*probeplt(:,(wherew(1,i)+plotj-1)).
end if.
do if (newplp > 1).
compute probeplt(:,(wheremw(((newplp*2)-3) ,i)+plotcnt))=probeplt(:,wherem((newplp-1),i))&*probeplt(:,(wherew(1,i)+plotj-1)).
end if.
compute plotcnt=plotcnt+1.
end loop.
end loop.
end if.
do if (zcmat((i+1),newplp))=1.
compute prodloop=1.
do if (newplp=1).
compute prodloop=nxvls.
end if.
compute plotcnt=0.
loop ploti = 1 to prodloop.
loop plotj = 1 to nzvls.
do if (newplp = 1).
compute probeplt(:,(wherexz(1,i)+plotcnt))=probeplt(:,(1+ploti))&*probeplt(:,(wherez(1,i)+plotj-1)).
end if.
do if (newplp > 1).
compute probeplt(:,(wheremz(((newplp*2)-3),i)+plotcnt))=probeplt(:,wherem((newplp-1),i))&*probeplt(:,(wherez(1,i)+plotj-1)).
end if.
compute plotcnt=plotcnt+1.
end loop.
end loop.
end if.
do if (wzcmat((i+1),newplp))=1.
compute plotcnt=0.
do if (threeway=0).
loop ploti = 1 to nwvls.
loop plotj = 1 to nzvls.
compute probeplt(:,(wherewz(1,i)+plotcnt))=probeplt(:,(wherew(1,i)+ploti-1))&*probeplt(:,(wherez(1,i)+plotj-1)).
compute plotcnt=plotcnt+1.
end loop.
end loop.
end if.
compute prodloop=1.
do if (newplp=1).
compute prodloop=nxvls.
end if.
compute plotcnt=0.
loop plotk = 1 to prodloop.
loop ploti = 1 to nwvls.
loop plotj = 1 to nzvls.
do if (newplp = 1).
compute probeplt(:,(wherexwz(1,i)+plotcnt))=probeplt(:,(1+plotk))&*probeplt(:,(wherew(1,i)+ploti-1))&*probeplt(:,(wherez(1,i)+plotj-1)).
end if.
do if (newplp > 1).
compute probeplt(:,(wheremwz(((newplp*2)-3),i)+plotcnt))=probeplt(:,wherem((newplp-1),i))&*probeplt(:,(wherew(1,i)+ploti-1))&*probeplt(:,(wherez(1,i)+plotj-1)).
end if.
compute plotcnt=plotcnt+1.
end loop.
end loop.
end loop.
end if.
end if.
end loop.
compute predvals=probeplt*b.
compute sepred=make(nrow(plotvals),3,999).
loop sei=1 to nrow(plotvals).
compute ask=probeplt(sei,:).
compute sepred(sei,1)=sqrt(ask*varb*t(ask)).
compute sepred(sei,2)=predvals(sei,1)-tval*sepred(sei,1).
compute sepred(sei,3)=predvals(sei,1)+tval*sepred(sei,1).
end loop.
compute prevloc=ncol(plotvals)+1.
compute probeplt={plotvals,predvals}.
do if (plot = 2).
compute probeplt={probeplt,sepred}.
end if.
compute didsome=0.
end if.
do if ((wzcmat((i+1),jmed)=1) and (printpbe <= intprobe)).
do if (jmed=1).
compute omnilp2=nxvls*nwvls.
compute omnitmp=ident(omnilp2).
compute omni3(wherexw(1,i):wherexw(2,i),:)=omnitmp.
end if.
do if (jmed>1).
compute omnilp2=nwvls.
compute omnitmp=ident(omnilp2).
compute omni3(wheremw(((jmed*2)-3),i):wheremw(((jmed*2)-2),i),:)=omnitmp.
end if.
compute omnif=make(1,4,0).
compute condeff3=0.
loop omnilp1=1 to nrow(zprobval).
loop omnilp=1 to (omnilp2).
do if (jmed=1).
compute omni3((wherexwz(1,i)+((omnilp-1)*nzvls)):(wherexwz(1,i)+((omnilp-1)*nzvls)+(nzvls-1)),omnilp)=t(zprobval(omnilp1,:)).
end if.
do if (jmed > 1).
compute omni3((wheremwz(((jmed*2)-3),i)+((omnilp-1)*nzvls)):(wheremwz(((jmed*2)-3),i)+((omnilp-1)*nzvls)+(nzvls-1)),omnilp)=t(zprobval(omnilp1,:)).
end if.
end loop.
compute condeff=t(omni3)*b.
compute condeff3={condeff3;condeff}.

.
compute lmat2= omni3.
do if ( 1 =0).
compute lmat2 = mdiag( omni3 ).
compute lmat3=make(nrow(lmat2),1,0).
loop flp=1 to ncol(lmat2).
do if (csum(lmat2(:,flp))=1).
compute lmat3={lmat3,lmat2(:,flp)}.
end if.
end loop.
compute lmat2=lmat3(:,2:ncol(lmat3)).
end if.
compute fratio = (t(t(lmat2)* b )*inv(t(lmat2)* varb *lmat2)*((t(lmat2)* b )))/ncol(lmat2).
compute pfr = 1-fcdf(fratio,ncol(lmat2),(n-nrow( b ))).
compute fresult={fratio,ncol(lmat2),(n-nrow( b )),pfr}.
do if ( 0 =1).
compute lmat3=1-rsum(lmat2).
compute xfm=make(n,csum(lmat3),0).
compute flpc=1.
loop flp=1 to nrow(lmat3).
do if (lmat3(flp,1)=1).
compute xfm(:,flpc)=x(:,flp).
compute flpc=flpc+1.
end if.
end loop.
compute bfm=inv(t(xfm)*xfm)*t(xfm)*y.
compute resid=y-(xfm*bfm).
compute sstotal=(y-(csum(y)/n)).
compute sstotal=csum(sstotal&*sstotal).
compute ssresid=csum(resid&*resid).
compute rsqch= 0 -((sstotal-ssresid)/sstotal).
compute fresult={rsqch,fresult}.
release xfm,flpc, resid, ssresid, bfm.
end if
.
compute omnif={omnif;fresult}.
end loop.
compute omnif=omnif(2:nrow(omnif),:).
compute clabtmp=znames.
compute condeff3=condeff3(2:nrow(condeff3),:).
do if ((nxvls*nwvls)=1).
compute omnif={condeff3,omnif}.
compute clabtmp={clabtmp,'Effect'}.
end if.
compute omnif={zmodvals,omnif}.
compute clabtmp={clabtmp,hcflab,'df1','df2','p'}.
do if (jmed=1).
print omnif/title='Test of conditional X*W interaction at value(s) of Z:'/cnames=clabtmp/format= F10.4.
end if.
do if (jmed>1).
print omnif/title='Test of conditional M*W interaction at value(s) of Z:'/cnames=clabtmp/format= F10.4.
end if.
release omni3.
end if.
loop probei = 1 to probexpl.
do if (probexpl > 1).
compute foctmp=make(nrow(modvals),probexpl,0).
compute foctmp(:,probei)=foctmp(:,probei)+1.
compute probtemp=make(nrow(modvals),1,0).
loop probem = 1 to numplps.
loop probek = 1 to nxvls.
loop probej=lpstsp(probem,1) to lpstsp(probem,2).
compute probtemp={probtemp,foctmp(:,probek)&*probeval(:,probej)}.
end loop.
end loop.
end loop.
compute probeva2=probtemp(:,2:ncol(probtemp)).
compute probeva2={foctmp,probeva2}.
end if.
compute probres=probeva2*probcoef.
compute probrese=sqrt(diag(probeva2*probvarb*t(probeva2))).
compute tratio = probres&/probrese.
compute p = 2*(1-tcdf(abs(tratio), dfres)).
compute modvals2={modvals,probres,probrese,tratio, p}.
compute modvals2={modvals2,(probres-tval&*probrese),(probres+tval&*probrese)}.
compute problabs={problabs,'Effect',hclab,'t', 'p', 'LLCI', 'ULCI'}.
do if (probexpl > 1 and (printpbe <= intprobe)).
do if (hasz = 1).
compute printz=1.
end if.
do if (hasw=1).
compute printw=1.
end if.
compute probrlab=make(nrow(modvals),1,xcatlab(probei,1)).
compute modvals3={modvals3; modvals2}.
compute probrow={probrow;probrown}.
compute probrown=probrown+1.
do if (probei=probexpl).
compute xproblab=xcatlab(1:nxvls,1).
compute probrow=probrow(2:nrow(probrow),1).
compute modvals3=modvals3(2:nrow(modvals3),:).
compute temp=modvals3.
compute temp(GRADE(probrow(:,1)),:)=modvals3.
compute modvals3=temp.
compute start2=1.
compute problabs=problabs(1,(1+(ncol(modvarl))):ncol(problabs)).
compute pstart=1.
loop probek= 1 to nrow(probeval).
compute endstart=start2+(nxvls-1).
compute temp=modvals3(start2:endstart,(1+ncol(modvarl)):ncol(modvals3)).
compute temp2=t(modvals3(start2:start2,1:ncol(modvarl))).
compute trnames=t(modvarl).
do if (probek > 1).
print/title='----------'/space=0.
else.
print/title = 'Conditional effects of the focal predictor at values of the moderator(s):'.
do if ((jmed=1) and (i = (nms+nys)) and (nms > 0)).
do if (nxvls = 1).
print/title = '(These are also the conditional direct effects of X on Y)'/space=0.
else.
print/title = '(These are also the relative conditional direct effects of X on Y)'/space=0.
end if.
end if.
print.
end if.
print temp2/title = 'Moderator value(s):'/rnames=trnames/format= F10.4 /space=0.
print temp/title = ' '/cnames=problabs/rnames=xproblab/format= F10.4 /space=0.
compute start2=start2+nxvls.
compute didsome=1.
do if (jmed=1).
compute mod1val=probeval(probek,1:nm1vls).
loop omnilp=1 to nxvls.
compute omni((wheremv1(1,i)+((omnilp-1)*nm1vls)):(wheremv1(1,i)+((omnilp-1)*nm1vls)+(nm1vls-1)),omnilp)=t(mod1val).
do if (nm1vls < ncol(probeval)).
compute mod2val=probeval(probek,(nm1vls+1):(nm1vls+nm2vls)).
compute omni((wheremv2(1,i)+((omnilp-1)*nm2vls)):(wheremv2(1,i)+((omnilp-1)*nm2vls)+(nm2vls-1)),omnilp)=t(mod2val).
do if ((nm1vls+nm2vls) < ncol(probeval)).
compute intlen=nm1vls*nm2vls.
compute modintvl=probeval(probek,(nm1vls+nm2vls+1):ncol(probeval)).
compute omni((wherexwz(1,i)+((omnilp-1)*intlen)):(wherexwz(1,i)+((omnilp-1)*intlen)+(intlen-1)),omnilp)=t(modintvl).
end if.
end if.
end loop.

.
compute lmat2= omni.
do if ( 1 =0).
compute lmat2 = mdiag( omni ).
compute lmat3=make(nrow(lmat2),1,0).
loop flp=1 to ncol(lmat2).
do if (csum(lmat2(:,flp))=1).
compute lmat3={lmat3,lmat2(:,flp)}.
end if.
end loop.
compute lmat2=lmat3(:,2:ncol(lmat3)).
end if.
compute fratio = (t(t(lmat2)* b )*inv(t(lmat2)* varb *lmat2)*((t(lmat2)* b )))/ncol(lmat2).
compute pfr = 1-fcdf(fratio,ncol(lmat2),(n-nrow( b ))).
compute fresult={fratio,ncol(lmat2),(n-nrow( b )),pfr}.
do if ( 0 =1).
compute lmat3=1-rsum(lmat2).
compute xfm=make(n,csum(lmat3),0).
compute flpc=1.
loop flp=1 to nrow(lmat3).
do if (lmat3(flp,1)=1).
compute xfm(:,flpc)=x(:,flp).
compute flpc=flpc+1.
end if.
end loop.
compute bfm=inv(t(xfm)*xfm)*t(xfm)*y.
compute resid=y-(xfm*bfm).
compute sstotal=(y-(csum(y)/n)).
compute sstotal=csum(sstotal&*sstotal).
compute ssresid=csum(resid&*resid).
compute rsqch= 0 -((sstotal-ssresid)/sstotal).
compute fresult={rsqch,fresult}.
release xfm,flpc, resid, ssresid, bfm.
end if
.
compute clabtmp={hcflab,'df1','df2','p'}.
print fresult/title='Test of equality of conditional means'/cnames=clabtmp/format= F10.4.
compute probetmp=probeplt(pstart:(pstart+nxvls),1).
compute probetmp={probetmp,probeplt(pstart:(pstart+nxvls),prevloc:ncol(probeplt))}.
compute pstart=pstart+(nxvls+1).
compute clabtmp={xnames, outnames(1,i), hclab, 'LLCI', 'ULCI'}.
print probetmp/title = 'Estimated conditional means being compared:'/cnames=clabtmp/format= F10.4.
end if.
end loop.
release probrow, start2, endstart, temp, temp2.
end if.
end if.
do if (probexpl = 1 and (printpbe <= intprobe)).
print/title = 'Conditional effects of the focal predictor at values of the moderator(s):'.
print modvals2/cnames=problabs/title = ' '/space=0/format= F10.4.
compute didsome=1.
do if (hasz = 1).
compute printz=1.
end if.
do if (hasw=1).
compute printw=1.
end if.
do if (jn = 1 and jnok=1).
do if (criterr = 0).
compute dfres=n-nrow(b).
compute jncrit =(dfres* (exp((dfres-(5/6))*((xp2/(dfres-(2/3)+(.11/dfres)))*(xp2/(dfres-(2/3)+(.11/dfres)))))-1)).
compute jnb1=b(wherejn1,1).
compute jnb3=b(wherejn3,1).
compute jnsb1=varb(wherejn1,wherejn1).
compute jnsb3=varb(wherejn3,wherejn3).
compute jnsb1b3=varb(wherejn1,wherejn3).
compute ajn =(jncrit*jnsb3)-(jnb3*jnb3).
compute bjn = 2*((jncrit*jnsb1b3)-(jnb1*jnb3)).
compute cjn = (jncrit*jnsb1)-(jnb1*jnb1).
compute radarg = (bjn*bjn)-(4*ajn*cjn).
compute den = 2*ajn.
compute nrts = 0.
do if (radarg >= 0 and den <> 0).
compute x21 = (-bjn+sqrt(radarg))/den.
compute x22 = (-bjn-sqrt(radarg))/den.
compute roots = 0.
do if (x21 >= jnmin and x21 <= jnmax).
compute nrts = 1.
compute roots = {roots; x21}.
end if.
do if (x22 >= jnmin and x22 <= jnmax).
compute nrts = nrts + 1.
compute roots = {roots; x22}.
end if.
compute roots={roots,make(nrow(roots),2,0)}.
end if.
do if (nrts > 0).
compute roots = roots(2:nrow(roots),1:3).
compute roots(1,2)=(csum(jnmod < roots(1,1))/n)*100.
compute roots(1,3)=(csum(jnmod > roots(1,1))/n)*100.
do if (nrow(roots)=2).
compute roots(2,2)=(csum(jnmod < roots(2,1))/n)*100.
compute roots(2,3)=(csum(jnmod > roots(2,1))/n)*100.
end if.
print roots/title = 'Moderator value(s) defining Johnson-Neyman significance region(s):'/clabels = 'Value', '% below', '% above'/format F10.4.
end if.
do if (nrts = 0).
print/title = 'There are no statistical significance transition points within the observed'.
print/title = 'range of the moderator found using the Johnson-Neyman method.'/space=0.
end if.
compute jnvals=make((21+nrts),7,0).
loop jni= 0 to 20.
compute jnvals((jni+1),1)=jnmin+(jni*((jnmax-jnmin)/20)).
end loop.
do if (nrts > 0).
loop jni = 1 to nrts.
loop jnj = 2 to nrow(jnvals).
do if ((roots(jni,1) > jnvals((jnj-1),1)) and (roots(jni,1) < jnvals(jnj,1))).
compute jnvals((jnj+1):(21+jni),1)=jnvals(jnj:(20+jni),1).
compute jnvals(jnj,1)=roots(jni,1).
end if.
end loop.
end loop.
end if.
loop jni = 1 to nrow(jnvals).
compute jnvals(jni,2)=jnb1+jnb3*jnvals(jni,1).
compute jnvals(jni,3)=sqrt(jnsb1+2*jnvals(jni,1)*jnsb1b3+(jnvals(jni,1)*jnvals(jni,1))*jnsb3).
compute jnvals(jni,4)=jnvals(jni,2)/jnvals(jni,3).
compute jnvals(jni,5)=2*(1-tcdf(abs(jnvals(jni,4)), dfres)).
compute jnvals(jni,6)=jnvals(jni,2)-sqrt(jncrit)*jnvals(jni,3).
compute jnvals(jni,7)=jnvals(jni,2)+sqrt(jncrit)*jnvals(jni,3).
end loop.
compute jnclbs={jnmodlab,'Effect',hclab,'t', 'p', 'LLCI', 'ULCI'}.
do if (((wcmat((i+1),jmed)=1) or (zcmat((i+1),jmed)=1)) and (wzcmat((i+1),jmed)=0)).
print jnvals/title = 'Conditional effect of focal predictor at values of the moderator:'/cnames =jnclbs/format = F10.4.
end if.
do if ((jmed = 1) and (wzcmat((i+1),jmed)=1)).
print jnvals/title = 'Conditional X*W interaction at values of the moderator Z:'/cnames =jnclbs/format = F10.4.
end if.
do if ((jmed > 1) and (wzcmat((i+1),jmed)=1)).
print jnvals/title = 'Conditional M*W interaction at values of the moderator Z:'/cnames =jnclbs/format = F10.4.
end if.
end if.
end if.
end if.
do if ((i = (nms+nys)) and (jmed=1) and (bcmat(nrow(bcmat),1)=1)).
do if (probei=1).
compute direfflb=problabs.
compute direff=modvals2.
end if.
do if (probei>1).
compute direff={direff;modvals2}.
end if.
end if.
compute intprint=0.
do if ((jmed=1) and (i=1) and nms=0) and modcok=1).
compute contvec2=make(2,1,1).
compute contvec2={contvec2,wcontval,zcontval}.
do if (wzcmat((i+1),jmed)=1).
loop conti= 1 to ncol(wcontval).
loop contj = 1 to ncol(zcontval).
compute contvec2={contvec2,wcontval(:,conti)&*zcontval(:,contj)}.
end loop.
end loop.
end if.
compute conteff=contvec2*probcoef.
compute contdiff=contvec2(1,:)-contvec2(2,:).
compute contse=sqrt(contdiff*probvarb*t(contdiff)).
compute conteffd=conteff(1,1)-conteff(2,1).
compute p=2*(1-tcdf(abs(conteffd/contse), dfres)).
compute contvec={contvec,conteff}.
print/title='Contrast between conditional effects of X:'.
print contvec/title=' '/rlabels='Effect1:','Effect2:'/cnames=problabs/format = F10.4 /space=0.
compute contvec={conteffd,contse,conteffd/contse, p}.
compute contvec={contvec,(conteffd-(tval*contse))}.
compute contvec={contvec,(conteffd+(tval*contse))}.
compute contlabs={'Contrast', hclab, 't', 'p', 'LLCI', 'ULCI'}.
print contvec/title='Test of Effect1 minus Effect2'/format= F10.4 /cnames=contlabs.
end if.
end loop.
do if (plot = 1 or plot = 2).
compute datalabs={t(focpred(:,3)),outnames(1,i)}.
do if (plot = 1).
compute datalabs={datalabs,'.'}.
end if.
do if (plot = 2).
compute datalabs={datalabs,'se', 'LLCI', 'ULCI.'}.
end if.
print/title = 'Data for visualizing the conditional effect of the focal predictor:'.
print/title = 'Paste text below into a SPSS syntax window and execute to produce plot.'/space=0.
compute dumb = {' ', ' ', ' ', ' ', ' ', ' ', ' '}.
print datalabs/title = 'DATA LIST FREE/'/format=A10.
print probeplt/title = 'BEGIN DATA.'/format.= F10.4 /space=0.
print/title = 'END DATA.'/space=0.
compute focgrph=datalabs(1,1).
compute graphix={focgrph,graphixs,modgrph}.
do if (((xdich=1) or (nxvls > 1)) and ((modcat=0) and (focgrph = xnames))).
compute graphix={modgrph,graphixs,focgrph}.
end if.
do if (panelgrp = 0).
compute graphix={graphix,'.'}.
else.
compute graphix={graphix,panelcde}.
end if.
print graphix/title = 'GRAPH/SCATTERPLOT='/format=A8/space=0.
end if.
end if.
end loop.
release jmed, intprint,didprint.
end if
.
end loop if criterr=1.
do if (criterr=0 and dototal = 1).
print/title = '************************** TOTAL EFFECT MODEL ****************************'.
print outnames(1,ncol(outnames))/title = 'OUTCOME VARIABLE:'/format = A8/space=0.
compute x=xtmp.
compute vlabsm={'constant';xcatlab(1:nxvls,1)}.
do if (ncs > 0).
compute x = {x,ctmp}.
compute vlabsm={vlabsm;t(covnames)}.
end if.
compute x = {ones,x}.

.
do if ( 1 =1).
compute b = inv(t( x )* x )*t( x )* y.
compute modres=b.
do if ( 1 =1).
compute n1=nrow( x ).
compute dfres=n1-(ncol( x )).
compute sstotal = t( y -(csum( y )/n1))*( y -(csum( y )/n1)).
compute resid= y - x *b.
compute ssresid = csum((resid)&**2).
compute r2 = (sstotal-ssresid)/sstotal.
compute adjr2 = 1-((1-r2)*(n1-1)/(dfres)).
compute mse=ssresid/(n1-ncol( x )).

.
compute n1=nrow( x ).
compute invXtX = inv(t( x )* x ).
compute varb = mse *invXtX.
compute k3 = ncol( x ).
compute xhc=0.
do if ( hc <> 5).
compute xhc= x.
compute hat = xhc(:,1).
loop i3=1 to nrow(xhc).
compute hat(i3,1)= xhc(i3,:)*invXtX*t(xhc(i3,:)).
end loop.
do if ( hc = 0 or hc =1).
loop i3 = 1 to k3.
compute xhc(:,i3)=xhc(:,i3)&* resid.
end loop.
end if.
do if ( hc =3 or hc =2).
loop i3=1 to k3.
compute xhc(:,i3) = ( resid &/(1-hat)&**(1/(4- hc )))&*xhc(:,i3).
end loop.
end if.
do if ( hc = 4).
compute hcmn=make(n,2,4).
compute hcmn(:,2)=(n1*hat)/k3.
loop i3= 1 to k3.
compute xhc(:,i3) = ( resid &/(1-hat)&**(rmin(hcmn)/2))&*xhc(:,i3).
end loop.
end if.
compute varb=(invXtX*t(xhc)*xhc*invXtX).
do if ( hc =1).
compute varb=(n1/(n1-ncol( x )))&*varb.
end if.
end if.
compute hclab={'se(HC0)','se(HC1)','se(HC2)','se(HC3)','se(HC4)','se'}.
compute hclab=hclab(1,( hc +1)).
compute hcflab={'F(HC0)','F(HC1)','F(HC2)','F(HC3)','F(HC4)','F'}.
compute hcflab=hcflab(1,( hc +1)).
release xhc
.
compute seb=sqrt(diag(varb)).
compute trat = b&/seb.
compute p = 2*(1-tcdf(abs(trat), (dfres))).
compute tval = sqrt(dfres* (exp((dfres-(5/6))*((xp2/(dfres-(2/3)+(.11/dfres)))*(xp2/(dfres-(2/3)+(.11/dfres)))))-1)).
compute modres={modres,seb,trat,p}.
compute modres={modres,(b-tval&*seb),(b+tval&*seb)}.
compute modresl={'coeff',hclab,'t','p','LLCI','ULCI'}.
compute lmat = ident(ncol( x )).
compute lmat = lmat(:,2:ncol(lmat)).
compute fratio = (t(t(lmat)*b)*inv(t(lmat)*varb*lmat)*((t(lmat)*b)))/(ncol( x )-1).
compute pfr = 1-fcdf(fratio,(ncol( x )-1),dfres).
compute modsum={sqrt(r2),r2,mse,fratio,(ncol( x )-1),dfres,pfr}.
compute modsuml={'R','R-sq','MSE',hcflab,'df1','df2', 'p'}.
end if.
end if
.
print modsum/title = 'Model Summary'/cnames = modsuml/format= F10.4.
print modres/title='Model'/rnames=vlabsm/cnames=modresl/format= F10.4.
compute toteff=modres(2:(1+nxvls),:).
compute totefflb=modresl.
compute toteffl2=vlabsm(2:(1+nxvls),:).
compute lmat=make(nrow(b),1,0).
compute lmat2=make(nxvls,1,1).
compute lmat(2:(1+nxvls),1)=lmat2.

.
compute lmat2= lmat.
do if ( 0 =0).
compute lmat2 = mdiag( lmat ).
compute lmat3=make(nrow(lmat2),1,0).
loop flp=1 to ncol(lmat2).
do if (csum(lmat2(:,flp))=1).
compute lmat3={lmat3,lmat2(:,flp)}.
end if.
end loop.
compute lmat2=lmat3(:,2:ncol(lmat3)).
end if.
compute fratio = (t(t(lmat2)* b )*inv(t(lmat2)* varb *lmat2)*((t(lmat2)* b )))/ncol(lmat2).
compute pfr = 1-fcdf(fratio,ncol(lmat2),(n-nrow( b ))).
compute fresult={fratio,ncol(lmat2),(n-nrow( b )),pfr}.
do if ( 1 =1).
compute lmat3=1-rsum(lmat2).
compute xfm=make(n,csum(lmat3),0).
compute flpc=1.
loop flp=1 to nrow(lmat3).
do if (lmat3(flp,1)=1).
compute xfm(:,flpc)=x(:,flp).
compute flpc=flpc+1.
end if.
end loop.
compute bfm=inv(t(xfm)*xfm)*t(xfm)*y.
compute resid=y-(xfm*bfm).
compute sstotal=(y-(csum(y)/n)).
compute sstotal=csum(sstotal&*sstotal).
compute ssresid=csum(resid&*resid).
compute rsqch= r2 -((sstotal-ssresid)/sstotal).
compute fresult={rsqch,fresult}.
release xfm,flpc, resid, ssresid, bfm.
end if
.
compute totomni=fresult.
do if (covcoeff=1).
print varb/title='Covariance matrix of regression parameter estimates:'/rnames=vlabsm /cnames=vlabsm/format= F10.4.
end if.
end if.
end if.
do if (criterr=0 and boot > 0).
compute bootres=make(1,rsum(nump),-999).
do if (effsize=1).
compute bootysd=make(1,1,-999).
compute bootxsd=make(1,1,-999).
end if.
compute badboot=0.
compute goodboot=0.
compute smallest=1.
loop j = 1 to maxboot.
compute nobootx=1.
compute modres2=999.
compute v=trunc(uniform(n,1)*n)+1.
compute bad=0.
loop i = 1 to (nms+nys).
compute y=outvars(v,i).
compute xindx=datindx(1:(nump(1,i)-1),i).
compute hello=0.
compute x = fulldat(v,xindx).
compute x={ones,x}.
compute xsq=t(x)*x.
compute exsq=eval(xsq).
release xsq.
compute holymoly=cmin(exsq).
compute zeroeig=csum(exsq <= 0.000000000002).
compute bad=bad + (zeroeig > 0).

.
compute desctmp=make((8-(4* 1 )),ncol( y ),-999).
loop jd=1 to ncol( y ).
compute descdat= y (:,jd).
compute desctmp(1,jd) = csum(descdat)/nrow(descdat).
compute desctmp(2,jd) = (nrow(descdat)*sscp(descdat))-(t(csum(descdat))*(csum(descdat))).
compute desctmp(2,jd) = sqrt(desctmp(2,jd)/(nrow(descdat)*(nrow(descdat)-1))).
compute desctmp(3,jd)=cmin(descdat).
compute desctmp(4,jd)=cmax(descdat).
do if ( 1 =0).
compute minwarn=0.
compute maxwarn=0.
do if ((desctmp(3,jd)=desctmp(4,jd)) and novar=0).
compute errcode(errs,1)=15.
compute errs=errs+1.
compute criterr=1.
compute novar=1.
end if.
compute tmp=((descdat(:,1)=desctmp(3,jd))+(descdat(:,1)=desctmp(4,jd))).
compute desctmp(8,jd)=(csum(tmp)=nrow(tmp)).
compute tmp = descdat.
compute tmp(GRADE(descdat),:) = descdat.
compute descdat = tmp.
release tmp.
compute decval={.16;.5;.84}.
loop kd=1 to 3.
compute low=trunc(decval(kd,1)*(nrow(descdat)+1)).
compute lowdec=decval(kd,1)*(nrow(descdat)+1)-low.
compute value=descdat(low,1)+(descdat((low+1),1)-descdat(low,1))*lowdec.
compute desctmp((4+kd),jd)=value.
end loop.
compute mnotev=1.
compute modvals=desctmp(5:7,:).
do if (quantile <> 1).
compute desctmp(5,jd)=desctmp(1,jd)-desctmp(2,jd).
compute desctmp(6,jd)=desctmp(1,jd).
compute desctmp(7,jd)=desctmp(1,jd)+desctmp(2,jd).
compute modvals=desctmp(5:7,:).
compute mnotev=2.
do if (modvals(1,1) < desctmp(3,1)).
compute modvals(1,1)=desctmp(3,1).
compute minwarn=1.
end if.
do if (modvals(3,1) > desctmp(4,1)).
compute modvals(3,1)=desctmp(4,1).
compute maxwarn=1.
end if.
end if.
do if (desctmp(8,1)=1).
compute modvals={desctmp(3,1);desctmp(4,1)}.
compute mnotev=0.
compute minwarn=0.
compute maxwarn=0.
end if.
end if.
end loop
.
compute bad=bad+ (desctmp(2,1) <= 0.00000000001).
do if (bad = 0).
do if (holymoly < smallest).
compute smallest=holymoly.
end if.

.
do if ( 1 =1).
compute b = inv(t( x )* x )*t( x )* y.
compute modres=b.
do if ( 0 =1).
compute n1=nrow( x ).
compute dfres=n1-(ncol( x )).
compute sstotal = t( y -(csum( y )/n1))*( y -(csum( y )/n1)).
compute resid= y - x *b.
compute ssresid = csum((resid)&**2).
compute r2 = (sstotal-ssresid)/sstotal.
compute adjr2 = 1-((1-r2)*(n1-1)/(dfres)).
compute mse=ssresid/(n1-ncol( x )).

.
compute n1=nrow( x ).
compute invXtX = inv(t( x )* x ).
compute varb = mse *invXtX.
compute k3 = ncol( x ).
compute xhc=0.
do if ( hc <> 5).
compute xhc= x.
compute hat = xhc(:,1).
loop i3=1 to nrow(xhc).
compute hat(i3,1)= xhc(i3,:)*invXtX*t(xhc(i3,:)).
end loop.
do if ( hc = 0 or hc =1).
loop i3 = 1 to k3.
compute xhc(:,i3)=xhc(:,i3)&* resid.
end loop.
end if.
do if ( hc =3 or hc =2).
loop i3=1 to k3.
compute xhc(:,i3) = ( resid &/(1-hat)&**(1/(4- hc )))&*xhc(:,i3).
end loop.
end if.
do if ( hc = 4).
compute hcmn=make(n,2,4).
compute hcmn(:,2)=(n1*hat)/k3.
loop i3= 1 to k3.
compute xhc(:,i3) = ( resid &/(1-hat)&**(rmin(hcmn)/2))&*xhc(:,i3).
end loop.
end if.
compute varb=(invXtX*t(xhc)*xhc*invXtX).
do if ( hc =1).
compute varb=(n1/(n1-ncol( x )))&*varb.
end if.
end if.
compute hclab={'se(HC0)','se(HC1)','se(HC2)','se(HC3)','se(HC4)','se'}.
compute hclab=hclab(1,( hc +1)).
compute hcflab={'F(HC0)','F(HC1)','F(HC2)','F(HC3)','F(HC4)','F'}.
compute hcflab=hcflab(1,( hc +1)).
release xhc
.
compute seb=sqrt(diag(varb)).
compute trat = b&/seb.
compute p = 2*(1-tcdf(abs(trat), (dfres))).
compute tval = sqrt(dfres* (exp((dfres-(5/6))*((xp2/(dfres-(2/3)+(.11/dfres)))*(xp2/(dfres-(2/3)+(.11/dfres)))))-1)).
compute modres={modres,seb,trat,p}.
compute modres={modres,(b-tval&*seb),(b+tval&*seb)}.
compute modresl={'coeff',hclab,'t','p','LLCI','ULCI'}.
compute lmat = ident(ncol( x )).
compute lmat = lmat(:,2:ncol(lmat)).
compute fratio = (t(t(lmat)*b)*inv(t(lmat)*varb*lmat)*((t(lmat)*b)))/(ncol( x )-1).
compute pfr = 1-fcdf(fratio,(ncol( x )-1),dfres).
compute modsum={sqrt(r2),r2,mse,fratio,(ncol( x )-1),dfres,pfr}.
compute modsuml={'R','R-sq','MSE',hcflab,'df1','df2', 'p'}.
end if.
end if
.
compute modres2={modres2,t(modres)}.
do if ( (bcmat((i+1),1) = 1) and (nobootx=1) and (effsize=1)).
compute nobootx=0.
compute xsdtemp= (nrow(x)*sscp(x(:,2)))-(t(csum(x(:,2)))*(csum(x(:,2)))).
compute xsdtemp= sqrt(xsdtemp/(nrow(x)*(nrow(x)-1))).
compute bootxsd={bootxsd;xsdtemp}.
end if.
end if.
end loop.
do if (bad = 0).
compute bootres={bootres;modres2(:,2:ncol(modres2))}.
do if (effsize=1).
compute ysdtemp= (nrow(y)*sscp(y))-(t(csum(y))*(csum(y))).
compute ysdtemp= sqrt(ysdtemp/(nrow(y)*(nrow(y)-1))).
compute bootysd={bootysd;ysdtemp}.
end if.
compute goodboot=goodboot+1.
end if.
do if (bad <> 0).
compute badboot=badboot+1.
end if.
end loop if (goodboot = boot).
compute bootres=bootres(2:nrow(bootres),:).
do if (effsize=1).
compute bootysd=bootysd(2:nrow(bootysd),:).
do if (nrow(bootxsd) > 1).
compute bootxsd=bootxsd(2:nrow(bootxsd),:).
end if.
end if.
do if (goodboot < (boot)).
compute boot=0.
compute modelbt=0.
compute notecode(notes,1) = 7.
compute notes = notes + 1.
end if.
do if (boot > 0).
do if (effsize=1).
compute bootysd={ysd;bootysd}.
compute bootxsd={xsd;bootxsd}.
end if.
do if (saveboot = 1).
save bootres/outfile = *.
end if.
compute bootcim=make(ncol(bootres),5,-99999).
compute bootcim(:,2) = t(csum(bootres)/nrow(bootres)).
compute bootcim(:,1) = coeffmat(2:nrow(coeffmat),1).
loop i = 1 to ncol(bootres).

.
compute temp = bootres(:,i).
compute temp(GRADE( bootres(:,i) )) = bootres(:,i).
compute badlo = 0.
compute badhi = 0.
do if ( 9999 <> 9999).
compute pv=csum(temp < 9999 )/nrow(temp).
compute ppv = pv.
do if (pv > .5).
compute ppv = 1-pv.
end if.
compute y5=sqrt(-2*ln(ppv)).
compute xp=y5+((((y5*p4+p3)*y5+p2)*y5+p1)*y5+p0)/((((y5*q4+q3)*y5+q2)*y5+q1)*y5+q0).
do if (pv <= .5).
compute xp = -xp.
end if.
compute cilow=rnd(nrow(temp)*(cdfnorm(2*xp+xp2))).
compute cihigh=trunc(nrow(temp)*(cdfnorm(2*xp+(-xp2))))+1.
do if (cilow < 1).
compute cilow = 1.
compute booterr=1.
compute badlo = 1.
end if.
do if (cihigh > nrow(temp)).
compute cihigh = boot.
compute booterr=1.
compute badhi = 1.
end if.
compute llcit=temp(cilow,1).
compute ulcit=temp(cihigh,1).
end if.
do if ( 9999 = 9999).
compute llcit=temp(cilow,1).
compute ulcit=temp(cihigh,1).
end if.
compute bootse=t(sqrt((cssq(temp)-((csum(temp)&**2)/nrow(temp)))/(nrow(temp)-1)))
.
compute bootcim(i,4:5)={llcit,ulcit}.
compute bootcim(i,3)=bootse.
end loop.
end if.
do if (badboot > 0).
compute notecode(notes,1) = 6.
compute notes = notes + 1.
end if.
end if.
do if (criterr=0).
release fulldat,x,y.
end if.

.
do if (criterr = 0 and nms > 0).
compute paths=paths(:,2:ncol(paths)).
compute pathsw=pathsw(:,2:ncol(pathsw)).
compute pathsz=pathsz(:,2:ncol(pathsz)).
compute pathswz=pathswz(:,2:ncol(pathswz)).
compute pathsmod=pathsw+pathsz+pathswz.
compute pathsdv=pathsdv(:,2:ncol(pathsdv)).
compute pathsfoc=pathsfoc(:,2:ncol(pathsfoc)).
compute pathtype=pathtype(:,2:ncol(pathtype)).
compute anymod=(rsum(pathsmod) > 0).
compute obscoeff=obscoeff(1,2:ncol(obscoeff)).
do if (dototal = 0).
print/title = '****************** DIRECT AND INDIRECT EFFECTS OF X ON Y *****************'.
end if.
do if (dototal = 1).
print/title = '************** TOTAL, DIRECT, AND INDIRECT EFFECTS OF X ON Y **************'.
compute totefflb(1,1)='Effect'.
do if (counterf <> 1).
do if (effsize=1).
compute toteffsz=toteff(:,1)/ysd.
compute totefflb={totefflb,'c_ps'}.
do if (xdich = 0 and mcx = 0).
compute toteffsz={toteffsz, (toteffsz*xsd)}.
compute totefflb={totefflb,'c_cs'}.
end if.
compute toteff={toteff,toteffsz}.
end if.
do if (nxvls > 1).
print toteff/title='Relative total effects of X on Y:'/rnames=toteffl2/cnames=totefflb/format= F10.4.
compute clabtmp={'R2-chng', hcflab, 'df1','df2','p'}.
print totomni/title='Omnibus test of total effect of X on Y:'/cnames=clabtmp/format= F10.4.
print/title= '----------'/space=0.
else.
print toteff/title='Total effect of X on Y'/cnames=totefflb/format= F10.4.
end if.
end if.
end if.
compute moddir=wcmat(nrow(bcmat),1)+zcmat(nrow(bcmat),1).
do if (bcmat(nrow(bcmat),1)=1 and counterf <> 1).
do if (moddir=0).
compute direfflb(1,1)='Effect'.
end if.
do if (effsize=1 and moddir=0 and anymod = 0).
compute direffsz=direff(:,1)/ysd.
compute direfflb={direfflb,'c''_ps'}.
do if (xdich = 0 and mcx = 0).
compute direffsz={direffsz, (direffsz*xsd)}.
compute direfflb={direfflb,'c''_cs'}.
end if.
compute direff={direff,direffsz}.
end if.
do if (moddir = 0 and nxvls=1).
print direff/title='Direct effect of X on Y'/cnames=direfflb/format= F10.4.
end if.
do if (moddir = 0 and nxvls>1).
print direff/title='Relative direct effects of X on Y'/rnames=direffl2/cnames=direfflb/format= F10.4.
compute clabtmp={'R2-chng', hcflab, 'df1','df2','p'}.
print diromni/title='Omnibus test of direct effect of X on Y:'/cnames=clabtmp/format= F10.4.
print/title= '----------'/space=0.
end if.
do if (moddir > 0 and nxvls=1).
print direff/title='Conditional direct effect(s) of X on Y:'/cnames=direfflb/format= F10.4.
end if.
do if (moddir > 0 and nxvls>1).
compute direffl2={' '}.
loop i = 1 to nxvls.
loop j = 1 to (nrow(direff)/nxvls).
compute direffl2={direffl2;xcatlab(i,1)}.
end loop.
end loop.
compute direffl2=direffl2(2:nrow(direffl2),1).
print direff/title='Relative conditional direct effect(s) of X on Y:'/cnames=direfflb/rnames=direffl2/format= F10.4.
end if.
end if.
do if (bcmat(nrow(bcmat),1)=0 and counterf <> 1).
print/title='The direct effect of X on Y is fixed to zero.'.
end if.
do if (nms = 1).
compute indmark={2}.
compute indsets={1,2}.
end if.
do if (nms = 2).
compute indmark={2,2,3}.
compute indsets={1,4,2,5,1,3,5}.
compute thetam=1.
end if.
do if (nms = 3).
compute indmark={2,2,2,3,3,3,4}.
compute indsets={1,7,2,8,4,9,1,3,8,1,5,9,2,6,9,1,3,6,9}.
compute thetam={1,2,3}.
end if.
do if (nms = 4).
compute indmark={2,2,2,2,3,3,3,3,3,3,4,4,4,4,5}.
compute indsets={1,11,2,12,4,13,7,14,1,3,12,1,5,13,1,8,14,2,6,13,2,9,14,4,10,14,1,3,6,13,1,3,9,14,1,5,10,14,2,6,10,14,1,3,6,10,14}.
compute thetam={1,2,4,3,5,6}.
end if.
do if (nms = 5).
compute indmark={2,2,2,2,2,3,3,3,3,3,3,3,3,3,3,4,4,4,4,4,4,4,4,4,4,5,5,5,5,5,6}.
compute indsets={1,16,2,17,4,18,7,19,11,20,1,3,17,1,5,18,1,8,19,1,12,20,2,6,18,2,9,19,2,13,20,4,10,19,4,14,20,7,15,20,1,3,6,18, 1,3,9,19,1,3,13,20,1,5,10,19,1,5,14,20,1,8,15,20,2,6,10,19,2,6,14,20,2,9,15,20,4,10,15,20,1,3,6,10,19, 1,3,6,14,20,1,3,9,15,20,1,5,10,15,20,2,6,10,15,20,1,3,6,10,15,20}.
compute thetam={1,2,5,3,6,8,4,7,9,10}.
end if.
do if (nms = 6).
compute indmark={2,2,2,2,2,2,3,3,3,3,3,3,3,3,3,3,3,3,3,3,3,4,4,4,4,4,4,4,4,4,4,4,4,4,4,4,4,4,4,4,4,5,5,5,5,5,5,5,5,5,5,5,5,5,5,5,6,6,6,6,6,6,7}.
compute indsets={1,22,2,23,4,24,7,25,11,26,16,27,1,3,23,1,5,24,1,8,25,1,12,26,1,17,27,2,6,24,2,9,25,2,13,26,2,18,27,4,10,25,4,14,26, 4,19,27,7,15,26,7,20,27,11,21,27,1,3,6,24,1,3,9,25,1,3,13,26,1,3,18,27,1,5,10,25,1,5,14,26,1,5,19,27,1,8,15,26,1,8,20,27, 1,12,21,27,2,6,10,25,2,6,14,26,2,6,19,27,2,9,15,26,2,9,20,27,2,13,21,27,4,10,15,26,4,10,20,27,4,14,21,27,7,15,21,27, 1,3,6,10,25,1,3,6,14,26,1,3,6,19,27,1,3,9,15,26,1,3,9,20,27,1,3,13,21,27,1,5,10,15,26,1,5,10,20,27,1,5,14,21,27, 1,8,15,21,27,2,6,10,15,26,2,6,10,20,27,2,6,14,21,27,2,9,15,21,27,4,10,15,21,27,1,3,6,10,15,26,1,3,6,10,20,27, 1,3,6,14,21,27,1,3,9,15,21,27,1,5,10,15,21,27,2,6,10,15,21,27,1,3,6,10,15,21,27}.
compute thetam={1,2,6,3,7,10,4,8,11,13,5,9,12,14,15}.
end if.
do if (nms = 7).
compute indmark={2,2,2,2,2,2,2}.
compute indsets={1,29,2,30,4,31,7,32,11,33,16,34,22,35}.
end if.
do if (nms = 8).
compute indmark={2,2,2,2,2,2,2,2}.
compute indsets={1,37,2,38,4,39,7,40,11,41,16,42,22,43,29,44}.
end if.
do if (nms = 9).
compute indmark={2,2,2,2,2,2,2,2,2}.
compute indsets={1,46,2,47,4,48,7,49,11,50,16,51,22,52,29,53,37,54}.
end if.
do if (nms=10).
compute indmark={2,2,2,2,2,2,2,2,2,2}.
compute indsets={1,56,2,57,4,58,7,59,11,60,16,61,22,62,29,63,37,64,46,65}.
end if.
compute indlbl = {'Ind1'; 'Ind2'; 'Ind3'; 'Ind4'; 'Ind5'; 'Ind6'; 'Ind7'; 'Ind8'; 'Ind9'; 'Ind10'; 'Ind11'; 'Ind12'; 'Ind13'; 'Ind14'; 'Ind15'}.
compute indlbl = {indlbl; 'Ind16';'Ind17';'Ind18';'Ind19';'Ind20';'Ind21';'Ind22';'Ind23';'Ind24';'Ind25';'Ind26';'Ind27';'Ind28';'Ind29';'Ind30'}.
compute indlbl = {indlbl; 'Ind31';'Ind32';'Ind33';'Ind34';'Ind35';'Ind36';'Ind37';'Ind38';'Ind39';'Ind40';'Ind41';'Ind42';'Ind43';'Ind44';'Ind45'}.
compute indlbl = {indlbl; 'Ind46';'Ind47';'Ind48';'Ind49';'Ind50';'Ind51';'Ind52';'Ind53';'Ind54';'Ind55';'Ind56';'Ind57';'Ind58';'Ind59';'Ind60'}.
compute indlbl = {indlbl; 'Ind61';'Ind62';'Ind63'}.
compute cntname={'(C1)';'(C2)';'(C3)';'(C4)';'(C5)';'(C6)';'(C7)';'(C8)';'(C9)';'(C10)';'(C11)';'(C12)';'(C13)';'(C14)';'(C15)';'(C16)';'(C17)'}.
compute cntname={cntname;'(C18)';'(C19)';'(C20)';'(C21)';'(C22)';'(C23)';'(C24)';'(C25)';'(C26)';'(C27)';'(C28)';'(C29)';'(C30)';'(C31)'}.
compute cntname={cntname;'(C32)';'(C33)';'(C34)';'(C35)';'(C36)';'(C37)';'(C38)';'(C39)';'(C40)';'(C41)';'(C42)';'(C43)';'(C44)';'(C45)'}.
compute cntname={cntname;'(C46)';'(C47)';'(C48)';'(C49)';'(C50)';'(C51)';'(C52)';'(C53)';'(C54)';'(C55)';'(C56)';'(C57)';'(C58)';'(C59)'}.
compute cntname={cntname;'(C60)';'(C61)';'(C62)';'(C63)';'(C64)';'(C65)';'(C66)';'(C67)';'(C68)';'(C69)';'(C70)';'(C71)';'(C72)';'(C73)'}.
compute cntname={cntname;'(C74)';'(C75)';'(C76)';'(C77)';'(C78)';'(C79)';'(C80)';'(C81)';'(C82)';'(C83)';'(C84)';'(C85)';'(C86)';'(C87)'}.
compute cntname={cntname;'(C88)';'(C89)';'(C90)';'(C91)';'(C92)';'(C93)';'(C94)';'(C95)';'(C96)';'(C97)';'(C98)';'(C99)';'(C100)';'(C101)'}.
compute cntname={cntname;'(C102)';'(C103)';'(C104)';'(C105)'}.
compute indmake=make(ncol(indmark),(nms+2),0).
compute indmod=make(ncol(indmark),1,999).
compute indmmm=make(ncol(indmark),1,0).
compute indmmmt=make(ncol(indmark),1,0).
compute start=1.
compute end=0.
compute nindfx=0.
compute indlocs=make(nrow(thetaxmb),ncol(paths),999).
compute indkey=make(ncol(indmark),1+((rmax(indmark)*2)+1),'     ').
compute c1=1.
compute c2=1.
compute c3=1.
loop i = 1 to ncol(paths).
do if (pathtype(1,i)=1).
compute indlocs(:,i)=thetaxmb(:,c1).
compute c1=c1+1.
end if.
do if (pathtype(1,i)=3).
compute indlocs(:,i)=thetamyb(:,c2).
compute c2=c2+1.
end if.
do if (pathtype(1,i)=2 and nms < 7 and serial=1).
compute indlocs(:,i)=thetammb(:,thetam(1,c3)).
compute c3=c3+1.
end if.
end loop.
loop i = 1 to ncol(indlocs).
compute c1=2.
loop j = 2 to nrow(indlocs).
do if (indlocs(j,i) <> 0).
compute indlocs(c1,i)=indlocs(j,i).
compute c1=c1+1.
end if.
end loop.
compute indlocs(1,i)=c1-2.
end loop.
compute indlocs=indlocs(1:rmax((indlocs(1,:))+1),:).
loop i = 1 to ncol(indmark).
compute numget=indmark(1,i).
compute end=end+numget.
compute gotcha=indsets(1,start:end).
compute start=end+1.
compute ok=1.
compute temp=0.
compute repoman=make(4,1,0).
loop j = 1 to ncol(gotcha).
do if paths(1,gotcha(1,j))=0.
compute ok=0.
end if.
do if (pathsmod(1,gotcha(1,j)) > 0).
compute temp=1.
compute temp2={pathsw(1,gotcha(1,j));pathsz(1,gotcha(1,j));pathswz(1,gotcha(1,j));0}.
do if (temp2(1,1)=1 and temp2(2,1)=1 and temp2(3,1)=0).
compute temp2(4,1)=1.
end if.
compute repoman=repoman+temp2.
end if.
end loop.
compute temp=0.
compute tempmmm=0.
compute typemmm=0.
do if ((repoman(1,1) > 0) and (repoman(2,1) = 0)).
compute temp=1.
do if (repoman(1,1)=1).
compute tempmmm=1.
end if.
do if (repoman(1,1) > 1 and (wdich=1 or mcw > 0)).
compute tempmmm=12.
compute typemmm=mcw.
do if (wdich=1).
compute typemmm=1.
end if.
end if.
do if (repoman(1,1) > 1 and (wdich=0 and mcw = 0)).
compute tempmmm=101.
end if.
end if.
do if ((repoman(1,1) = 0) and (repoman(2,1) > 0)).
compute temp=2.
do if (repoman(2,1)=1).
compute tempmmm=2.
end if.
do if (repoman(2,1) > 1 and (zdich = 1 or mcz > 0)).
compute tempmmm=22.
compute typemmm=mcz.
do if (zdich=1).
compute typemmm=1.
end if.
end if.
do if (repoman(2,1) > 1 and (zdich = 0 and mcw = 0)).
compute tempmmm=102.
end if.
end if.
do if (repoman(1,1)>0 and repoman(2,1)>0).
compute temp=3.
do if (repoman(1,1)=1 and repoman(2,1)=1).
do if (repoman(4,1)=1).
compute tempmmm=31.
end if.
do if (repoman(3,1)=1).
compute tempmmm=41.
end if.
end if.
end if.
do if (repoman(1,1)=1 and repoman(2,1)=1 and repoman(3,1)=0 and repoman(4,1)=0).
compute tempmmm=51.
end if.
do if (ok = 1).
compute nindfx=nindfx+1.
compute indmake(nindfx,1)=numget.
compute indmod(nindfx,1)=temp.
compute indmmm(nindfx,1)=tempmmm.
compute indmmmt(nindfx,1)=typemmm.
compute indmake(nindfx,2:(1+numget))=gotcha.
compute indkey(nindfx,1)=xnames.
loop j = 1 to numget.
compute indkey(nindfx,(j*2+1))=pathsdv(1,gotcha(1,j)).
compute indkey(nindfx,(j*2))='   ->   '.
end loop.
end if.
end loop.
compute indkey=indkey(1:nindfx,1:((cmax(indmake(:,1))*2)+1)).
compute indmake=indmake(1:nindfx,1:(cmax(indmake(:,1))+1)).
compute indmod=indmod(1:nrow(indmake),1).
compute indmmm=indmmm(1:nrow(indmake),1).
compute indmmmt=indmmmt(1:nrow(indmake),1).
compute ncpairs = (((nindfx)*(nindfx-1))/2).
do if ((contrast = 1 or contrast = 2) and (ncpairs > 105)).
compute contrast=0.
compute notecode(notes,1) = 13.
compute notes = notes + 1.
end if.
do if (contrast = 3).
do if (ncol(contvec) <> nindfx).
compute contrast=0.
compute notecode(notes,1) = 14.
compute notes = notes + 1.
end if.
end if.
do if (anymod=0).
do if (nms = 1 and contrast > 0).
compute contrast=0.
end if.
compute efloop=(((1-(effsize=0))*2)+1)-(((mcx>0 or xdich=1))*(1-(effsize=0))).
loop kk=1 to efloop.
do if (boot = 0).
compute bootres=obscoeff.
compute indtab=999.
compute inddiff=999.
compute bootysd=ysd.
compute bootxsd=xsd.
end if.
do if (boot > 0).
compute bootres={obscoeff;bootres}.
compute indtab=make(1,4,999).
compute inddiff=make(nrow(bootres),1,999).
end if.
compute indtotal=make(nrow(bootres),1,0).
loop i = 1 to nrow(indmake).
loop j = 1 to nxvls.
compute indtemp=make(nrow(bootres),1,1).
loop k = 1 to indmake(i,1).
compute jtemp=1.
do if (j > 1 and k=1).
compute jtemp=j.
end if.
compute indtemp=indtemp&*bootres(:,pathsfoc(jtemp,indmake(i,(k+1)))).
end loop.
do if (kk = 2).
compute indtemp=indtemp/bootysd.
end if.
do if (kk = 3).
compute indtemp=(bootxsd&*indtemp)/bootysd.
end if.
do if (contrast <> 0).
compute inddiff={inddiff,indtemp}.
end if.
do if (nxvls=1).
compute indtotal=indtotal+indtemp.
end if.
compute indeff=indtemp(1,1).
do if (boot > 0).

.
compute temp = indtemp(2:nrow(indtemp),1).
compute temp(GRADE( indtemp(2:nrow(indtemp),1) )) = indtemp(2:nrow(indtemp),1).
compute badlo = 0.
compute badhi = 0.
do if ( 9999 <> 9999).
compute pv=csum(temp < 9999 )/nrow(temp).
compute ppv = pv.
do if (pv > .5).
compute ppv = 1-pv.
end if.
compute y5=sqrt(-2*ln(ppv)).
compute xp=y5+((((y5*p4+p3)*y5+p2)*y5+p1)*y5+p0)/((((y5*q4+q3)*y5+q2)*y5+q1)*y5+q0).
do if (pv <= .5).
compute xp = -xp.
end if.
compute cilow=rnd(nrow(temp)*(cdfnorm(2*xp+xp2))).
compute cihigh=trunc(nrow(temp)*(cdfnorm(2*xp+(-xp2))))+1.
do if (cilow < 1).
compute cilow = 1.
compute booterr=1.
compute badlo = 1.
end if.
do if (cihigh > nrow(temp)).
compute cihigh = boot.
compute booterr=1.
compute badhi = 1.
end if.
compute llcit=temp(cilow,1).
compute ulcit=temp(cihigh,1).
end if.
do if ( 9999 = 9999).
compute llcit=temp(cilow,1).
compute ulcit=temp(cihigh,1).
end if.
compute bootse=t(sqrt((cssq(temp)-((csum(temp)&**2)/nrow(temp)))/(nrow(temp)-1)))
.
compute indeff={indeff,bootse,llcit,ulcit}.
end if.
compute indtab={indtab;indeff}.
end loop.
end loop.
compute indtab=indtab(2:nrow(indtab),:).
compute rowlbs=indlbl(1:nrow(indtab),1).
do if (mc > 0).
compute inddiff=make(mc,1,-999).
compute indtab2=make(nrow(indtab),4,-999).
compute indtab2(:,1)=indtab.
compute indtab=indtab2.
release indtab2.
compute mcct=0.
compute indtotal=make(mc,1,0).
do if (kk = 1).
compute x1 = sqrt(-2*ln(uniform(mc,nrow(mcsopath))))&*cos((2*3.14159265358979)*uniform(mc,nrow(mcsopath))).
compute x1=x1*chol(indcov).
loop ii=1 to nrow(x1).
compute x1(ii,:)=x1(ii,:)+t(mcsopath).
end loop.
end if.
loop ii=1 to nms.
compute tmpb=x1(:,((nms*nxvls)+ii)).
compute tmpb2=tmpb.
do if (nxvls > 1).
loop jj=1 to (nxvls-1).
compute tmpb2={tmpb2,tmpb}.
end loop.
end if.
compute indtemp=x1(:,(((ii-1)*nxvls)+1):(ii*nxvls))&*tmpb2.
loop jj=1 to ncol(indtemp).
do if (kk = 2).
compute indtemp(:,jj)=indtemp(:,jj)/ysd.
end if.
do if (kk = 3).
compute indtemp(:,jj)=(xsd*indtemp(:,jj))/ysd.
end if.

.
compute temp = indtemp(:,jj).
compute temp(GRADE( indtemp(:,jj) )) = indtemp(:,jj).
compute badlo = 0.
compute badhi = 0.
do if ( 9999 <> 9999).
compute pv=csum(temp < 9999 )/nrow(temp).
compute ppv = pv.
do if (pv > .5).
compute ppv = 1-pv.
end if.
compute y5=sqrt(-2*ln(ppv)).
compute xp=y5+((((y5*p4+p3)*y5+p2)*y5+p1)*y5+p0)/((((y5*q4+q3)*y5+q2)*y5+q1)*y5+q0).
do if (pv <= .5).
compute xp = -xp.
end if.
compute cilow=rnd(nrow(temp)*(cdfnorm(2*xp+xp2))).
compute cihigh=trunc(nrow(temp)*(cdfnorm(2*xp+(-xp2))))+1.
do if (cilow < 1).
compute cilow = 1.
compute booterr=1.
compute badlo = 1.
end if.
do if (cihigh > nrow(temp)).
compute cihigh = boot.
compute booterr=1.
compute badhi = 1.
end if.
compute llcit=temp(cilow,1).
compute ulcit=temp(cihigh,1).
end if.
do if ( 9999 = 9999).
compute llcit=temp(cilow,1).
compute ulcit=temp(cihigh,1).
end if.
compute bootse=t(sqrt((cssq(temp)-((csum(temp)&**2)/nrow(temp)))/(nrow(temp)-1)))
.
compute mcct=mcct+1.
compute indtab(mcct,2:4)={bootse,llcit,ulcit}.
end loop.
do if (nxvls=1).
compute indtotal=indtotal+indtemp.
do if (contrast <> 0).
compute inddiff={inddiff,indtemp}.
end if.
end if.
end loop.
release indtemp,tmpb.
end if.
do if (normal = 1 and sobelok=1).
compute sobelmat=indtab(:,1).
compute sobelmat={sobelmat,(sobelmat/2),sobelmat,sobelmat}.
loop ii=1 to nms.
compute se2b=indcov(((nms*nxvls)+ii),((nms*nxvls)+ii)).
compute bpath2=mcsopath(((nms*nxvls)+ii),1)&**2.
compute se2a=diag(indcov((((ii-1)*nxvls)+1):(ii*nxvls),(((ii-1)*nxvls)+1):(ii*nxvls))).
compute apath2=mcsopath((((ii-1)*nxvls)+1):(ii*nxvls) ,1)&**2.
compute sesobel=sqrt(apath2*se2b+bpath2*se2a+se2a*se2b).
compute sobelmat((((ii-1)*nxvls)+1):(ii*nxvls),2)=sesobel.
end loop.
release se2b,bpath2,se2a,apath2,sesobel,ii.
compute sobelmat(:,3)=sobelmat(:,1)&/sobelmat(:,2).
compute sobelmat(:,4) = 2*(1-cdfnorm(abs(sobelmat(:,3)))).
end if.
do if (serial = 0).
compute rowlbs=t(mnames).
end if.
do if (nxvls=1 and nms > 1).
compute rowlbs={'TOTAL';rowlbs}.
compute indtemp=indtotal(1,1).
do if (boot > 0 and nxvls=1).

.
compute temp = indtotal(2:nrow(indtotal),1).
compute temp(GRADE( indtotal(2:nrow(indtotal),1) )) = indtotal(2:nrow(indtotal),1).
compute badlo = 0.
compute badhi = 0.
do if ( 9999 <> 9999).
compute pv=csum(temp < 9999 )/nrow(temp).
compute ppv = pv.
do if (pv > .5).
compute ppv = 1-pv.
end if.
compute y5=sqrt(-2*ln(ppv)).
compute xp=y5+((((y5*p4+p3)*y5+p2)*y5+p1)*y5+p0)/((((y5*q4+q3)*y5+q2)*y5+q1)*y5+q0).
do if (pv <= .5).
compute xp = -xp.
end if.
compute cilow=rnd(nrow(temp)*(cdfnorm(2*xp+xp2))).
compute cihigh=trunc(nrow(temp)*(cdfnorm(2*xp+(-xp2))))+1.
do if (cilow < 1).
compute cilow = 1.
compute booterr=1.
compute badlo = 1.
end if.
do if (cihigh > nrow(temp)).
compute cihigh = boot.
compute booterr=1.
compute badhi = 1.
end if.
compute llcit=temp(cilow,1).
compute ulcit=temp(cihigh,1).
end if.
do if ( 9999 = 9999).
compute llcit=temp(cilow,1).
compute ulcit=temp(cihigh,1).
end if.
compute bootse=t(sqrt((cssq(temp)-((csum(temp)&**2)/nrow(temp)))/(nrow(temp)-1)))
.
compute indtemp={indtemp, bootse,llcit,ulcit}.
end if.
do if (mc > 0).
compute obtmc=indtab(:,1).
compute indtemp=csum(obtmc).

.
compute temp = indtotal(:,1).
compute temp(GRADE( indtotal(:,1) )) = indtotal(:,1).
compute badlo = 0.
compute badhi = 0.
do if ( 9999 <> 9999).
compute pv=csum(temp < 9999 )/nrow(temp).
compute ppv = pv.
do if (pv > .5).
compute ppv = 1-pv.
end if.
compute y5=sqrt(-2*ln(ppv)).
compute xp=y5+((((y5*p4+p3)*y5+p2)*y5+p1)*y5+p0)/((((y5*q4+q3)*y5+q2)*y5+q1)*y5+q0).
do if (pv <= .5).
compute xp = -xp.
end if.
compute cilow=rnd(nrow(temp)*(cdfnorm(2*xp+xp2))).
compute cihigh=trunc(nrow(temp)*(cdfnorm(2*xp+(-xp2))))+1.
do if (cilow < 1).
compute cilow = 1.
compute booterr=1.
compute badlo = 1.
end if.
do if (cihigh > nrow(temp)).
compute cihigh = boot.
compute booterr=1.
compute badhi = 1.
end if.
compute llcit=temp(cilow,1).
compute ulcit=temp(cihigh,1).
end if.
do if ( 9999 = 9999).
compute llcit=temp(cilow,1).
compute ulcit=temp(cihigh,1).
end if.
compute bootse=t(sqrt((cssq(temp)-((csum(temp)&**2)/nrow(temp)))/(nrow(temp)-1)))
.
compute indtemp={indtemp, bootse,llcit,ulcit}.
end if.
compute indtab={indtemp;indtab}.
end if.
compute bootlbs={'Effect', 'BootSE','BootLLCI','BootULCI'}.
do if (mc > 0).
compute bootlbs={'Effect', 'MC SE','MC LLCI','MC ULCI'}.
end if.
do if (nxvls = 1).
do if (contrast <> 0).
compute inddiff=inddiff(:,2:ncol(inddiff)).
do if (mc > 0).
compute inddiff={t(obtmc);inddiff}.
end if.
do if (contrast = 3).
compute inddifft=inddiff*t(contvec).
compute indtemp=inddifft(1,1).
do if (boot > 0 or mc > 0).

.
compute temp = inddifft(2:nrow(inddifft),1).
compute temp(GRADE( inddifft(2:nrow(inddifft),1) )) = inddifft(2:nrow(inddifft),1).
compute badlo = 0.
compute badhi = 0.
do if ( 9999 <> 9999).
compute pv=csum(temp < 9999 )/nrow(temp).
compute ppv = pv.
do if (pv > .5).
compute ppv = 1-pv.
end if.
compute y5=sqrt(-2*ln(ppv)).
compute xp=y5+((((y5*p4+p3)*y5+p2)*y5+p1)*y5+p0)/((((y5*q4+q3)*y5+q2)*y5+q1)*y5+q0).
do if (pv <= .5).
compute xp = -xp.
end if.
compute cilow=rnd(nrow(temp)*(cdfnorm(2*xp+xp2))).
compute cihigh=trunc(nrow(temp)*(cdfnorm(2*xp+(-xp2))))+1.
do if (cilow < 1).
compute cilow = 1.
compute booterr=1.
compute badlo = 1.
end if.
do if (cihigh > nrow(temp)).
compute cihigh = boot.
compute booterr=1.
compute badhi = 1.
end if.
compute llcit=temp(cilow,1).
compute ulcit=temp(cihigh,1).
end if.
do if ( 9999 = 9999).
compute llcit=temp(cilow,1).
compute ulcit=temp(cihigh,1).
end if.
compute bootse=t(sqrt((cssq(temp)-((csum(temp)&**2)/nrow(temp)))/(nrow(temp)-1)))
.
compute indtemp={indtemp, bootse,llcit,ulcit}.
end if.
compute indtab={indtab;indtemp}.
end if.
do if (contrast = 1 or contrast = 2).
compute conkey=make(1,4,' ').
loop i = 1 to ncol(inddiff)-1.
loop j = (i+1) to ncol(inddiff).
compute inddifft=inddiff(:,i)-inddiff(:,j).
do if (contrast=2).
compute inddifft=abs(inddiff(:,i))-abs(inddiff(:,j)).
end if.
compute indtemp=inddifft(1,1).
compute conkeyt={' ', rowlbs((i+1),1),' minus  ',rowlbs((j+1),1)}.
compute conkey={conkey;conkeyt}.
do if (boot > 0 or mc > 0).

.
compute temp = inddifft(2:nrow(inddifft),1).
compute temp(GRADE( inddifft(2:nrow(inddifft),1) )) = inddifft(2:nrow(inddifft),1).
compute badlo = 0.
compute badhi = 0.
do if ( 9999 <> 9999).
compute pv=csum(temp < 9999 )/nrow(temp).
compute ppv = pv.
do if (pv > .5).
compute ppv = 1-pv.
end if.
compute y5=sqrt(-2*ln(ppv)).
compute xp=y5+((((y5*p4+p3)*y5+p2)*y5+p1)*y5+p0)/((((y5*q4+q3)*y5+q2)*y5+q1)*y5+q0).
do if (pv <= .5).
compute xp = -xp.
end if.
compute cilow=rnd(nrow(temp)*(cdfnorm(2*xp+xp2))).
compute cihigh=trunc(nrow(temp)*(cdfnorm(2*xp+(-xp2))))+1.
do if (cilow < 1).
compute cilow = 1.
compute booterr=1.
compute badlo = 1.
end if.
do if (cihigh > nrow(temp)).
compute cihigh = boot.
compute booterr=1.
compute badhi = 1.
end if.
compute llcit=temp(cilow,1).
compute ulcit=temp(cihigh,1).
end if.
do if ( 9999 = 9999).
compute llcit=temp(cilow,1).
compute ulcit=temp(cihigh,1).
end if.
compute bootse=t(sqrt((cssq(temp)-((csum(temp)&**2)/nrow(temp)))/(nrow(temp)-1)))
.
compute indtemp={indtemp, bootse,llcit,ulcit}.
end if.
compute indtab={indtab;indtemp}.
end loop.
end loop.
end if.
release inddiff.
compute contlbs=cntname(1:(((nindfx)*(nindfx-1))/2),1).
compute rowlbs={rowlbs;contlbs}.
end if.
do if (kk=1).
print indtab/title = 'Indirect effect(s) of X on Y:'/rnames=rowlbs/cnames=bootlbs/format= F10.4.
end if.
do if (kk = 2).
print indtab/title = 'Partially standardized indirect effect(s) of X on Y:'/rnames=rowlbs/cnames=bootlbs/format= F10.4.
end if.
do if (kk = 3).
print indtab/title = 'Completely standardized indirect effect(s) of X on Y:'/rnames=rowlbs/cnames=bootlbs/format= F10.4.
end if.
do if (normal=1 and sobelok=1 and kk=1).
compute sobellab={'Effect',hclab,'Z','p'}.
compute sobelrlb=rowlbs.
do if (nms > 1).
compute sobelrlb=rowlbs(2:(1+nms),1).
end if.
print sobelmat/title='   Normal theory test for indirect effect(s):'/cnames=sobellab/rnames=sobelrlb/format= F10.4.
end if.
do if (contrast <> 0).
do if ((contrast=1 or contrast = 2) and kk=efloop ).
compute conkey=conkey(2:nrow(conkey),:).
print conkey/title = 'Specific indirect effect contrast definition(s):'/rnames=contlbs/format=A8.
end if.
do if (contrast = 3 and kk=efloop).
compute crowlbs=rowlbs(2:(nindfx+1),1).
print contvec/title = 'Specific indirect effect contrast weights:'/cnames=crowlbs/rlabels='(C1)'/format= F10.4.
end if.
do if (contrast = 2 and kk=efloop).
print/title = 'Contrasts are differences between absolute values of indirect effects'.
end if.
end if.
do if (serial = 1 and kk=efloop).
compute rowlbst=rowlbs(2:nrow(rowlbs),1).
print indkey/title = 'Indirect effect key:'/rnames=rowlbst/format = A8.
end if.
else.
do if (kk = 1).
print/title = 'Relative indirect effects of X on Y'.
end if.
do if (kk = 2).
print/title = 'Partially standardized relative indirect effect(s) of X on Y:'.
end if.
do if (kk = 3).
print/title = 'Completely standardized relative indirect effect(s) of X on Y:'.
end if.
loop i = 1 to nrow(indmake).
compute indtabsm=indtab((((i-1)*nxvls)+1):(nxvls*i),:).
compute indkeyt=indkey(i,:).
print indkeyt/title=' '/space=0/format=A8.
print indtabsm/title = ' '/cnames=bootlbs/rnames=direffl2/format= F10.4 /space=0.
do if (normal=1 and sobelok=1 and kk=1).
compute sobelsm=sobelmat((((i-1)*nxvls)+1):(nxvls*i),:).
compute sobellab={'Effect',hclab,'Z','p'}.
print sobelsm/title='      Normal theory test for relative indirect effects:'/cnames=sobellab/rnames=direffl2/format= F10.4.
end if.
end loop.
end if.
do if (effsize = 1 and boot > 0).
compute bootres=bootres(2:nrow(bootres),:).
end if.
end loop.
end if.
do if (anymod > 0).
do if (boot = 0).
compute bootres=obscoeff.
compute indtab=999.
end if.
do if (boot > 0).
compute bootres={obscoeff;bootres}.
compute indtab=make(1,4,999).
end if.
do if (csum((indmod > 0))=nrow(indmod)).
do if (nxvls > 1).
print/title= 'Relative conditional indirect effects of X on Y:'.
end if.
do if (nxvls = 1).
print/title= 'Conditional indirect effects of X on Y:'.
end if.
end if.
do if (csum((indmod > 0)) < nrow(indmod)).
do if (nxvls > 1).
print/title= 'Relative conditional and unconditional indirect effects of X on Y:'.
end if.
do if (nxvls = 1).
print/title= 'Conditional and unconditional indirect effects of X on Y:'.
end if.
end if.
loop i = 1 to nrow(indmake).
compute indtab=indtab(1,:)*0.
print indkey(i,:)/title = 'INDIRECT EFFECT:'/format=A8.
do if (indmod(i,1)=0).
loop j = 1 to nxvls.
compute indtemp=make(nrow(bootres),1,1).
loop k = 1 to indmake(i,1).
compute jtemp=1.
do if (j > 1 and k=1).
compute jtemp=j.
end if.
compute indtemp=indtemp&*bootres(:,pathsfoc(jtemp,indmake(i,(k+1)))).
end loop.
compute indeff=indtemp(1,1).
do if (boot > 0).

.
compute temp = indtemp(2:nrow(indtemp),1).
compute temp(GRADE( indtemp(2:nrow(indtemp),1) )) = indtemp(2:nrow(indtemp),1).
compute badlo = 0.
compute badhi = 0.
do if ( 9999 <> 9999).
compute pv=csum(temp < 9999 )/nrow(temp).
compute ppv = pv.
do if (pv > .5).
compute ppv = 1-pv.
end if.
compute y5=sqrt(-2*ln(ppv)).
compute xp=y5+((((y5*p4+p3)*y5+p2)*y5+p1)*y5+p0)/((((y5*q4+q3)*y5+q2)*y5+q1)*y5+q0).
do if (pv <= .5).
compute xp = -xp.
end if.
compute cilow=rnd(nrow(temp)*(cdfnorm(2*xp+xp2))).
compute cihigh=trunc(nrow(temp)*(cdfnorm(2*xp+(-xp2))))+1.
do if (cilow < 1).
compute cilow = 1.
compute booterr=1.
compute badlo = 1.
end if.
do if (cihigh > nrow(temp)).
compute cihigh = boot.
compute booterr=1.
compute badhi = 1.
end if.
compute llcit=temp(cilow,1).
compute ulcit=temp(cihigh,1).
end if.
do if ( 9999 = 9999).
compute llcit=temp(cilow,1).
compute ulcit=temp(cihigh,1).
end if.
compute bootse=t(sqrt((cssq(temp)-((csum(temp)&**2)/nrow(temp)))/(nrow(temp)-1)))
.
compute indeff={indeff,bootse,llcit,ulcit}.
end if.
compute indtab={indtab;indeff}.
end loop.
do if (nxvls > 1).
compute indefflb=xcatlab(1:nxvls,1).
print indtab(2:nrow(indtab),:)/title = ' '/clabels='Effect', 'BootSE', 'BootLLCI', 'BootULCI'/rnames=indefflb/format= F10.4 /space=0.
end if.
do if (nxvls = 1).
print indtab(2:nrow(indtab),:)/title = ' '/clabels='Effect', 'BootSE', 'BootLLCI', 'BootULCI'/format= F10.4 /space=0.
end if.
end if.
do if (indmod(i,1)>0).
do if (indmod(i,1)=1).
compute indmodva=wmodvals.
compute indprova=wprobval.
compute condlbs={wnames}.
compute printw=1.
else if (indmod(i,1)=2).
compute indmodva=zmodvals.
compute indprova=zprobval.
compute condlbs={znames}.
compute printz=1.
else if (indmod(i,1)=3).
compute cntmp=1.
compute printz=1.
compute printw=1.
compute indmodva=make((nrow(wmodvals)*nrow(zmodvals)),2,999).
loop k7 = 1 to nrow(wmodvals).
loop k8 = 1 to nrow(zmodvals).
compute indmodva(cntmp,:)={wmodvals(k7,1),zmodvals(k8,1)}.
compute cntmp=cntmp+1.
end loop.
end loop.
compute condlbs={wnames,znames}.
end if.
compute condres=make(nrow(indmodva),1,999).
do if (boot > 0).
compute condres=make(nrow(indmodva),4,999).
end if.
compute condres={indmodva,condres}.
loop k4 = 1 to nxvls.
compute imm3=make(nrow(bootres),1,1).
compute imm4=make(nrow(bootres),1,1).
compute indcontr=0.
do if (indmod(i,1)=3).
compute tihsw=wprobval.
compute tihsz=zprobval.
end if.
loop k1=1 to nrow(indmodva).
compute tucker2=make(nrow(bootres),1,1).
compute imm2=make(nrow(bootres),1,1).
compute wfirst=0.
compute zfirst=0.
compute immset=0.
loop k2=1 to indmake(i,1).
compute colnumb=indmake(i,(k2+1)).
do if (k2=1).
compute wbb=make(nrow(bootres),(nwvls*nxvls),0).
compute zbb=make(nrow(bootres),(nzvls*nxvls),0).
compute wzbb=make(nrow(bootres),(nwvls*nzvls*nxvls),0).
end if.
do if (k2<>1).
compute wbb=make(nrow(bootres),nwvls,0).
compute zbb=make(nrow(bootres),nzvls,0).
compute wzbb=make(nrow(bootres),(nwvls*nzvls),0).
end if.
compute cnt=1.
compute tihs=indlocs(2:((indlocs(1,colnumb))+1),colnumb).
do if (k2 = 1).
compute focbb=tihs(1:nxvls,1).
compute focbb=bootres(:,focbb).
do if (indmmm(i,1)>0).
compute imm=focbb(:,k4).
compute condbb=imm.
end if.
compute focaddon=make(1,nxvls,0).
compute focaddon(1,k4)=1.
compute cnt=cnt+nxvls.
compute placeh=nxvls.
do if (indmod(i,1)=1).
compute tihsz=make(nrow(wprobval),(nzvls*nxvls),0).
compute tihswz=make(nrow(wprobval),(nwvls*nzvls*nxvls),0).
do if (pathsw(1,colnumb)=1).
compute temp=make(nrow(wprobval),(nxvls*nwvls),0).
loop k5 = 1 to nrow(wprobval).
loop k6=1 to nwvls.
compute temp(k5, (((k4-1)*nwvls)+k6))=wprobval(k5,k6).
end loop.
end loop.
compute indprova={temp,tihsz,tihswz}.
else.
compute indprova={wprobval,tihsz,tihswz}.
end if.
end if.
do if (indmod(i,1)=2).
compute tihsw=make(nrow(zprobval),(nwvls*nxvls),0).
compute tihswz=make(nrow(zprobval),(nwvls*nzvls*nxvls),0).
do if (pathsz(1,colnumb)=1).
compute temp=make(nrow(zprobval),(nxvls*nzvls),0).
loop k5 = 1 to nrow(zprobval).
loop k6 =1 to nzvls.
compute temp(k5,(((k4-1)*nzvls)+k6))=zprobval(k5,k6).
end loop.
end loop.
compute indprova={tihsw,temp,tihswz}.
else.
compute indprova={tihsw,zprobval,tihswz}.
end if.
end if.
do if (indmod(i,1)=3).
compute indprova=make((nrow(wprobval)*nrow(zprobval)),((ncol(wprobval)*nxvls)+(ncol(zprobval)*nxvls)+(nwvls*nzvls*nxvls)),0).
compute cntemp=1.
loop k7=1 to nrow(wprobval).
loop k8 =1 to nrow(zprobval).
compute temp=wprobval(k7,:)*focaddon(1,k4).
compute indprova(cntemp,(((k4-1)*nwvls)+1):(k4*(nwvls)))=temp.
compute temp=zprobval(k8,:)*focaddon(1,k4).
compute indprova(cntemp, ((((k4-1)*nzvls)+1)+(nxvls*nwvls)) : ((((k4-1)*nzvls)+1)+(nxvls*nwvls)+(nzvls-1)))=temp.
compute cntemp=cntemp+1.
end loop.
end loop.
do if (pathsz(1,colnumb)=0).
compute temp=make(nrow(indprova),(ncol(zprobval)*nxvls),0).
compute indprova(:,((ncol(wprobval)*nxvls)+1):((ncol(wprobval)+ncol(zprobval))*nxvls))=temp.
end if.
do if (pathsw(1,colnumb)=0).
compute temp=make(nrow(indprova),(ncol(wprobval)*nxvls),0).
compute indprova(:,1:(ncol(wprobval)*nxvls))=temp.
end if.
do if (pathswz(1,colnumb)=1).
compute cntemp=(ncol(wprobval)*nxvls)+(ncol(zprobval)*nxvls)+((k4-1)*ncol(wprobval)*ncol(zprobval))+1.
loop k7=1 to ncol(wprobval).
loop k8=1 to ncol(zprobval).
compute indprova(:,cntemp)=indprova(:,((ncol(wprobval)*(k4-1))+k7))&*indprova(:,((((k4-1)*ncol(zprobval))+k8)+(nxvls*ncol(wprobval)))).
compute cntemp=cntemp+1.
end loop.
end loop.
end if.
end if.
end if.
do if (k2 > 1).
compute focbb=tihs(1,1).
compute focbb=bootres(:,focbb).
do if (indmmm(i,1)>0).
compute imm=focbb(:,1).
compute condbb=imm.
end if.
compute focaddon=1.
compute cnt=cnt+1.
compute placeh=1.
do if (indmod(i,1)=1).
compute tihsz=make(nrow(wprobval),nzvls,0).
compute tihswz=make(nrow(wprobval),(nwvls*nzvls),0).
compute indprova={wprobval,tihsz,tihswz}.
end if.
do if (indmod(i,1)=2).
compute tihsw=make(nrow(zprobval),nwvls,0).
compute tihswz=make(nrow(zprobval),(nwvls*nzvls),0).
compute indprova={tihsw,zprobval,tihswz}.
end if.
do if (indmod(i,1)=3).
compute indprova=make((nrow(wprobval)*nrow(zprobval)),((ncol(wprobval)+ncol(zprobval))+(nwvls*nzvls)),0).
compute cntemp=1.
loop k7=1 to nrow(wprobval).
loop k8 =1 to nrow(zprobval).
compute indprova(cntemp,1:(ncol(wprobval)+ncol(zprobval)))={wprobval(k7,:),zprobval(k8,:)}.
compute cntemp=cntemp+1.
end loop.
end loop.
do if (pathsz(1,colnumb)=0).
compute temp=make(nrow(indprova),ncol(zprobval),0).
compute indprova(:,(ncol(wprobval)+1):(ncol(wprobval)+ncol(zprobval)))=temp.
end if.
do if (pathsw(1,colnumb)=0).
compute temp=make(nrow(indprova),ncol(wprobval),0).
compute indprova(:,1:ncol(wprobval))=temp.
end if.
do if (pathswz(1,colnumb)=1).
compute cntemp=ncol(wprobval)+ncol(zprobval)+1.
loop k7=1 to ncol(wprobval).
loop k8=1 to ncol(zprobval).
compute indprova(:,cntemp)=indprova(:,k7)&*indprova(:,(ncol(wprobval)+k8)).
compute cntemp=cntemp+1.
end loop.
end loop.
end if.
end if.
end if.
do if (pathsw(1,colnumb)) = 1.
compute wbb=tihs(cnt:(cnt+(placeh*nwvls)-1),1).
compute wbb=bootres(:,wbb).
compute immlbs2=wcatlab(1:nwvls,1).
do if (zfirst=0).
compute wfirst=1.
end if.
do if (indmmm(i,1)=1 or indmmm(i,1)=31 or indmmm(i,1)=51).
compute imm=wbb(:,1).
loop k7 = 1 to nwvls.
compute imm={imm,wbb(:,(((k4-1)*nwvls*(k2=1))+k7))}.
end loop.
compute imm=imm(:,2:ncol(imm)).
end if.
do if (indmmm(i,1)=41 or indmmm(i,1)=51).
compute condbb=make(nrow(bootres),1,0).
loop k7 = 1 to nwvls.
compute condbb={condbb,wbb(:,(((k4-1)*nwvls*(k2=1))+k7))}.
end loop.
compute condbb=condbb(:,2:ncol(condbb)).
end if.
compute cnt=cnt+(placeh*nwvls).
end if.
do if (pathsz(1,colnumb)) = 1.
compute zbb=tihs(cnt:(cnt+(placeh*nzvls)-1),1).
compute zbb=bootres(:,zbb).
do if (wfirst=0).
compute zfirst=1.
end if.
do if (indmmm(i,1) <> 31).
compute immlbs2=zcatlab(1:nzvls,1).
end if.
do if (indmmm(i,1)=2 or indmmm(i,1)=31 or indmmm(i,1)=51).
do if (indmmm(i,1)=2).
compute imm=zbb(:,1).
end if.
loop k7 = 1 to nzvls.
compute imm={imm,zbb(:,(((k4-1)*nzvls*(k2=1))+k7))}.
end loop.
do if (indmmm(i,1)=2 or indmmm(i,1)=51).
compute imm=imm(:,2:ncol(imm)).
do if (indmmm(i,1)=51).
compute condbb={condbb,imm}.
end if.
end if.
end if.
compute cnt=cnt+(placeh*nzvls).
end if.
do if (pathswz(1,colnumb)) = 1.
compute wzbb=tihs(cnt:(cnt+(placeh*nwvls*nzvls)-1),1).
compute wzbb=bootres(:,wzbb).
do if (indmmm(i,1)=41).
compute imm=wzbb(:,1).
loop k7=1 to nwvls*nzvls.
compute imm={imm,wzbb(:,(((k4-1)*nzvls*nwvls*(k2=1))+k7))}.
end loop.
end if.
do if (indmmm(i,1)=41).
compute imm=imm(:,2:ncol(imm)).
compute condbb={condbb,imm(:,(ncol(imm)-(nwvls*nzvls)+1):ncol(imm))}.
end if.
compute cnt=cnt+(placeh*nzvls*nwvls).
end if.
compute indprobe={focaddon,indprova(k1,:)}.
compute tucker={focbb,wbb,zbb,wzbb}.
loop k3=1 to ncol(indprobe).
compute tucker(:,k3)=tucker(:,k3)*indprobe(1,k3).
end loop.
compute tucker2=tucker2&*rsum(tucker).
do if (indmmm(i,1) = 1 or indmmm(i,1)=2 or indmmm(i,1)=31 or indmmm(i,1)=41 or indmmm(i,1)=51).
do if (immset=1).
do if (ncol(imm2)=1 and ncol(imm) = 1).
compute imm2=imm2&*imm.
end if.
do if (indmmm(i,1)=41 or indmmm(i,1)=51).
do if ((ncol(condbb2) > 1) and (ncol(condbb)>1)).
compute condbb2t=make(nrow(condbb2),(ncol(condbb2)*ncol(condbb)),-999999).
compute k9=1.
do if (wfirst=1).
loop k7=1 to ncol(condbb2).
loop k8 = 1 to ncol(condbb).
compute condbb2t(:,k9)=condbb2(:,k7)&*condbb(:,k8).
compute k9=k9+1.
end loop.
end loop.
end if.
do if (zfirst=1).
loop k7=1 to ncol(condbb).
loop k8 = 1 to ncol(condbb2).
compute imm2t(:,k9)=condbb(:,k7)&*condbb2(:,k8).
compute k9=k9+1.
end loop.
end loop.
end if.
compute condbb2=condbb2t.
release condbb2t.
end if.
do if ((ncol(condbb2) > 1) and (ncol(condbb)=1)).
loop k7 = 1 to ncol(condbb2).
compute condbb2(:,k7)=condbb2(:,k7)&*condbb.
end loop.
end if.
do if ((ncol(condbb2) = 1) and (ncol(condbb)>1)).
loop k7 = 1 to ncol(condbb).
compute condbb(:,k7)=condbb2&*condbb(:,k7).
end loop.
compute condbb2=condbb.
end if.
end if.
do if (ncol(imm2) <> 1 and ncol(imm) <> 1).
compute imm2t=make(nrow(imm2),(ncol(imm2)*ncol(imm)),-999999).
compute k9=1.
do if (wfirst=1).
loop k7=1 to ncol(imm2).
loop k8 = 1 to ncol(imm).
compute imm2t(:,k9)=imm2(:,k7)&*imm(:,k8).
compute k9=k9+1.
end loop.
end loop.
end if.
do if (zfirst=1).
loop k7=1 to ncol(imm).
loop k8 = 1 to ncol(imm2).
compute imm2t(:,k9)=imm(:,k7)&*imm2(:,k8).
compute k9=k9+1.
end loop.
end loop.
end if.
compute imm2=imm2t.
release imm2t.
end if.
do if ((ncol(imm2) > 1) and (ncol(imm)=1)).
loop k7=1 to ncol(imm2).
compute imm2(:,k7)=imm2(:,k7)&*imm.
end loop.
end if.
do if ((ncol(imm2) = 1) and (ncol(imm) > 1)).
loop k7=1 to ncol(imm).
compute imm(:,k7)=imm2&*imm(:,k7).
end loop.
compute imm2=imm.
end if.
end if.
do if (immset=0).
compute imm2=imm.
do if (indmmm(i,1)=41 or indmmm(i,1)=51).
compute condbb2=condbb.
end if.
compute immset=1.
end if.
end if.
end loop.
compute indtemp=tucker2(1,1).
do if (indmmm(i,1)=12 or indmmm(i,1)=22).
compute imm3={imm3,tucker2}.
do if (k1=nrow(indmodva)).
compute imm3=imm3(:,2:ncol(imm3)).
compute immstop=ncol(imm3).
loop k8=2 to immstop.
do if (indmmmt(i,1)=1).
compute imm3={imm3,(imm3(:,k8)-imm3(:,1))}.
end if.
do if (indmmmt(i,1)=2).
compute imm3={imm3,(imm3(:,k8)-imm3(:,(k8-1)))}.
end if.
do if (indmmmt(i,1)=3).
compute imm3={imm3,((rsum(imm3(:,(k8:immstop)))/(immstop-k8+1))-imm3(:,(k8-1)))}.
end if.
do if (indmmmt(i,1)=4).
compute imm3={imm3,(imm3(:,k8)-(rsum(imm3(:,1:immstop))/immstop))}.
end if.
end loop.
do if (indmmmt(i,1)<5).
compute imm2=imm3(:,(immstop+1):ncol(imm3)).
release imm3.
end if.
end if.
end if.
do if (indmmm(i,1)>-1 and (contrast = 1 or contrast = 2)).
compute imm4={imm4,tucker2}.
do if (k1=nrow(indmodva) and k1 > 1).
compute imm4=imm4(:,2:ncol(imm4)).
compute immstop=ncol(imm4).
compute condcont=make((immstop*(immstop-1)/2),6,-999).
loop k8 = 1 to (immstop-1).
loop k9 = (k8+1) to immstop.
do if (contrast=1).
compute imm4={imm4,(imm4(:,k9)-imm4(:,k8))}.
end if.
do if (contrast=2).
compute imm4={imm4,(abs(imm4(:,k9))-abs(imm4(:,k8)))}.
end if.
compute condcont((ncol(imm4)-immstop),1)=imm4(1,k9).
compute condcont((ncol(imm4)-immstop),2)=imm4(1,k8).
end loop.
end loop.
compute imm4=imm4(:,(immstop+1):ncol(imm4)).
loop k8=1 to ncol(imm4).
compute condcont(k8,3)=imm4(1,k8).
do if (boot > 0).

.
compute temp = imm4(2:nrow(imm4),k8).
compute temp(GRADE( imm4(2:nrow(imm4),k8) )) = imm4(2:nrow(imm4),k8).
compute badlo = 0.
compute badhi = 0.
do if ( 9999 <> 9999).
compute pv=csum(temp < 9999 )/nrow(temp).
compute ppv = pv.
do if (pv > .5).
compute ppv = 1-pv.
end if.
compute y5=sqrt(-2*ln(ppv)).
compute xp=y5+((((y5*p4+p3)*y5+p2)*y5+p1)*y5+p0)/((((y5*q4+q3)*y5+q2)*y5+q1)*y5+q0).
do if (pv <= .5).
compute xp = -xp.
end if.
compute cilow=rnd(nrow(temp)*(cdfnorm(2*xp+xp2))).
compute cihigh=trunc(nrow(temp)*(cdfnorm(2*xp+(-xp2))))+1.
do if (cilow < 1).
compute cilow = 1.
compute booterr=1.
compute badlo = 1.
end if.
do if (cihigh > nrow(temp)).
compute cihigh = boot.
compute booterr=1.
compute badhi = 1.
end if.
compute llcit=temp(cilow,1).
compute ulcit=temp(cihigh,1).
end if.
do if ( 9999 = 9999).
compute llcit=temp(cilow,1).
compute ulcit=temp(cihigh,1).
end if.
compute bootse=t(sqrt((cssq(temp)-((csum(temp)&**2)/nrow(temp)))/(nrow(temp)-1)))
.
compute condcont(k8,4:6)={bootse,llcit,ulcit}.
end if.
end loop.
do if (boot=0).
compute condcont=condcont(:,1:3).
end if.
compute indcontr=1.
end if.
end if.
do if (boot > 0).

.
compute temp = tucker2(2:nrow(tucker2),1).
compute temp(GRADE( tucker2(2:nrow(tucker2),1) )) = tucker2(2:nrow(tucker2),1).
compute badlo = 0.
compute badhi = 0.
do if ( 9999 <> 9999).
compute pv=csum(temp < 9999 )/nrow(temp).
compute ppv = pv.
do if (pv > .5).
compute ppv = 1-pv.
end if.
compute y5=sqrt(-2*ln(ppv)).
compute xp=y5+((((y5*p4+p3)*y5+p2)*y5+p1)*y5+p0)/((((y5*q4+q3)*y5+q2)*y5+q1)*y5+q0).
do if (pv <= .5).
compute xp = -xp.
end if.
compute cilow=rnd(nrow(temp)*(cdfnorm(2*xp+xp2))).
compute cihigh=trunc(nrow(temp)*(cdfnorm(2*xp+(-xp2))))+1.
do if (cilow < 1).
compute cilow = 1.
compute booterr=1.
compute badlo = 1.
end if.
do if (cihigh > nrow(temp)).
compute cihigh = boot.
compute booterr=1.
compute badhi = 1.
end if.
compute llcit=temp(cilow,1).
compute ulcit=temp(cihigh,1).
end if.
do if ( 9999 = 9999).
compute llcit=temp(cilow,1).
compute ulcit=temp(cihigh,1).
end if.
compute bootse=t(sqrt((cssq(temp)-((csum(temp)&**2)/nrow(temp)))/(nrow(temp)-1)))
.
compute indtemp={indtemp, bootse,llcit,ulcit}.
end if.
compute condres(k1,(ncol(indmodva)+1):ncol(condres))=indtemp.
end loop.
compute condlbs={condlbs,'Effect'}.
do if (boot > 0).
compute condlbs={condlbs,'BootSE', 'BootLLCI', 'BootULCI'}.
end if.
do if (nxvls=1).
print condres/title=' '/cnames=condlbs/format= F10.4 /space=0.
else.
compute condrlb=make(nrow(condres),1,xcatlab(k4,1)).
print condres/title=' '/cnames=condlbs/rnames=condrlb/format= F10.4 /space=0.
end if.
compute dichadj=0.
compute immcat=0.
do if (indmmm(i,1)>0).
do if (indmmm(i,1)=1 or indmmm(i,1)=12 or indmmm(i,1)=31).
do if (wdich=1 and mcw=0).
do if (indmmm(i,1) <> 12).
compute imm2(:,1)=imm2(:,1)*(wmax-wmin).
end if.
do if (indmmm(i,1) <> 31).
compute dichadj=1.
end if.
end if.
do if ((mcw = 1 or mcw = 2) and indmmm(i,1) <> 31)).
compute immcat=1.
end if.
end if.
do if (indmmm(i,1)=2 or indmmm(i,1)=22 or indmmm(i,1)=31).
do if (zdich=1 and mcz=0).
do if (indmmm(i,1) = 31).
compute imm2(:,(nwvls+1):ncol(imm2))=imm2(:,(nwvls+1):ncol(imm2))*(zmax-zmin).
end if.
do if (indmmm(i,1) = 2).
compute imm2(:,1)=imm2(:,1)*(zmax-zmin).
end if.
do if (indmmm(i,1) <> 31).
compute dichadj=1.
end if.
end if.
do if ((mcz = 1 or mcz = 2) and indmmm(i,1) <> 31)).
compute immcat=1.
end if.
end if.
compute immtemp2=t(imm2(1,:)).
compute immtemp=immtemp2.
compute immlbs={'Index'}.
do if (boot > 0).
compute immtemp=make(1,3,0).
loop k7=1 to ncol(imm2).

.
compute temp = imm2(2:nrow(imm2),k7).
compute temp(GRADE( imm2(2:nrow(imm2),k7) )) = imm2(2:nrow(imm2),k7).
compute badlo = 0.
compute badhi = 0.
do if ( 9999 <> 9999).
compute pv=csum(temp < 9999 )/nrow(temp).
compute ppv = pv.
do if (pv > .5).
compute ppv = 1-pv.
end if.
compute y5=sqrt(-2*ln(ppv)).
compute xp=y5+((((y5*p4+p3)*y5+p2)*y5+p1)*y5+p0)/((((y5*q4+q3)*y5+q2)*y5+q1)*y5+q0).
do if (pv <= .5).
compute xp = -xp.
end if.
compute cilow=rnd(nrow(temp)*(cdfnorm(2*xp+xp2))).
compute cihigh=trunc(nrow(temp)*(cdfnorm(2*xp+(-xp2))))+1.
do if (cilow < 1).
compute cilow = 1.
compute booterr=1.
compute badlo = 1.
end if.
do if (cihigh > nrow(temp)).
compute cihigh = boot.
compute booterr=1.
compute badhi = 1.
end if.
compute llcit=temp(cilow,1).
compute ulcit=temp(cihigh,1).
end if.
do if ( 9999 = 9999).
compute llcit=temp(cilow,1).
compute ulcit=temp(cihigh,1).
end if.
compute bootse=t(sqrt((cssq(temp)-((csum(temp)&**2)/nrow(temp)))/(nrow(temp)-1)))
.
compute temp={bootse,llcit,ulcit}.
compute immtemp={immtemp;temp}.
end loop.
compute immtemp=immtemp(2:nrow(immtemp),:).
compute immtemp={immtemp2,immtemp}.
compute immlbs={immlbs,'BootSE', 'BootLLCI', 'BootULCI'}.
end if.
do if (dichadj=0 and immcat=0 and indmmmt(i,1)<>5 and indmmm(i,1) < 100 ).
do if (indmmm(i,1) < 30).
print immtemp/title='      Index of moderated mediation:'/cnames=immlbs/rnames=immlbs2/format= F10.4.
end if.
do if (indmmm(i,1) = 31).
compute immlbs2={immlbs2;zcatlab(1:nzvls,1)}.
print immtemp/title='      Indices of partial moderated mediation:'/cnames=immlbs/rnames=immlbs2/format= F10.4.
end if.
do if (indmmm(i,1) = 41 or indmmm(i,1)=51).
loop k7=1 to nwvls.
compute immlbs2=zcatlab(1:nzvls,1).
compute immtemp2=immtemp((((k7-1)*nzvls)+1):(((k7-1)*nzvls)+nzvls),:).
do if (nwvls > 1).
compute primodv={'        ', wcatlab(k7,1)}.
print primodv/title='      Primary moderator:'/format=A8.
end if.
do if (nzvls=1).
print immtemp2/title='      Index of moderated moderated mediation'/cnames=immlbs/format= F10.4.
else.
print immtemp2/title='      Indices of moderated moderated mediation'/cnames=immlbs/rnames=immlbs2/format= F10.4.
end if.
compute cmmtemp=make(nrow(zprobval),4,0).
loop k8=1 to nrow(zprobval).
compute condbb3=condbb2(:,((nwvls+1)+((k7-1)*nzvls)):((nwvls+1)+((k7-1)*nzvls)+(nzvls-1))).
do if (ncol(zprobval) > 1).
compute condbb3=condbb3*mdiag(zprobval(k8,:)).
else.
compute condbb3=condbb3&*zprobval(k8,:).
end if.
compute condbb3={condbb2(:,k7),condbb3}.
compute icmm=rsum(condbb3).
compute cmmtemp(k8,1)=icmm(1,1).
do if (boot > 0).

.
compute temp = icmm(2:nrow(icmm),1).
compute temp(GRADE( icmm(2:nrow(icmm),1) )) = icmm(2:nrow(icmm),1).
compute badlo = 0.
compute badhi = 0.
do if ( 9999 <> 9999).
compute pv=csum(temp < 9999 )/nrow(temp).
compute ppv = pv.
do if (pv > .5).
compute ppv = 1-pv.
end if.
compute y5=sqrt(-2*ln(ppv)).
compute xp=y5+((((y5*p4+p3)*y5+p2)*y5+p1)*y5+p0)/((((y5*q4+q3)*y5+q2)*y5+q1)*y5+q0).
do if (pv <= .5).
compute xp = -xp.
end if.
compute cilow=rnd(nrow(temp)*(cdfnorm(2*xp+xp2))).
compute cihigh=trunc(nrow(temp)*(cdfnorm(2*xp+(-xp2))))+1.
do if (cilow < 1).
compute cilow = 1.
compute booterr=1.
compute badlo = 1.
end if.
do if (cihigh > nrow(temp)).
compute cihigh = boot.
compute booterr=1.
compute badhi = 1.
end if.
compute llcit=temp(cilow,1).
compute ulcit=temp(cihigh,1).
end if.
do if ( 9999 = 9999).
compute llcit=temp(cilow,1).
compute ulcit=temp(cihigh,1).
end if.
compute bootse=t(sqrt((cssq(temp)-((csum(temp)&**2)/nrow(temp)))/(nrow(temp)-1)))
.
compute cmmtemp(k8,2:4)={bootse,llcit,ulcit}.
end if.
end loop.
compute cmmtemp={zmodvals,cmmtemp}.
do if (boot=0).
compute cmmtemp=cmmtemp(:,1:2).
end if.
compute cmmlbs={znames,immlbs}.
print cmmtemp/title='      Indices of conditional moderated mediation by W'/cnames=cmmlbs/format= F10.4.
end loop.
end if.
end if.
do if (dichadj=1 or immcat=1 and indmmm(i,1) < 30).
print immtemp/title='Index of moderated mediation (difference between conditional indirect effects):'/cnames=immlbs/rnames=immlbs2/format= F10.4.
end if.
end if.
do if (indcontr=1).
compute condctlb={'Effect1','Effect2','Contrast','BootSE', 'BootLLCI','BootULCI'}.
print condcont/title=' Pairwise contrasts between conditional indirect effects (Effect1 minus Effect2)'/cnames=condctlb/format= F10.4.
end if.
print/title= '---'/space=0.
end loop.
end if.
end loop.
end if.
end if
.
do if (criterr=0 and (saveest = 1 or saveboot=1)).
compute coeffsav=coeffmat(2:nrow(coeffmat),:).
compute conseq=conseq(2:nrow(conseq),1).
compute dfmat=dfmat(2:nrow(dfmat),1).
compute dfmat=dfmat.
compute saven={'conseqnt','antecdnt','coeff','se','t','p','LLCI','ULCI','df'}.
do if (saveest=1).
save {conseq,vlabs,coeffsav,dfmat}/outfile = */names=saven/strings=conseqnt antecdnt.
end if.
do if (saveboot=1 and boot > 0 ).
print/title = '**************************************************************************'.
print/title='Bootstrap estimates were saved to a file'/space=0.
compute colslab=make(300,1,' ').


compute colslab( 1 ,1)= 'COL1'.


compute colslab( 2 ,1)= 'COL2'.


compute colslab( 3 ,1)= 'COL3'.


compute colslab( 4 ,1)= 'COL4'.


compute colslab( 5 ,1)= 'COL5'.


compute colslab( 6 ,1)= 'COL6'.


compute colslab( 7 ,1)= 'COL7'.


compute colslab( 8 ,1)= 'COL8'.


compute colslab( 9 ,1)= 'COL9'.


compute colslab( 10 ,1)= 'COL10'.


compute colslab( 11 ,1)= 'COL11'.


compute colslab( 12 ,1)= 'COL12'.


compute colslab( 13 ,1)= 'COL13'.


compute colslab( 14 ,1)= 'COL14'.


compute colslab( 15 ,1)= 'COL15'.


compute colslab( 16 ,1)= 'COL16'.


compute colslab( 17 ,1)= 'COL17'.


compute colslab( 18 ,1)= 'COL18'.


compute colslab( 19 ,1)= 'COL19'.


compute colslab( 20 ,1)= 'COL20'.


compute colslab( 21 ,1)= 'COL21'.


compute colslab( 22 ,1)= 'COL22'.


compute colslab( 23 ,1)= 'COL23'.


compute colslab( 24 ,1)= 'COL24'.


compute colslab( 25 ,1)= 'COL25'.


compute colslab( 26 ,1)= 'COL26'.


compute colslab( 27 ,1)= 'COL27'.


compute colslab( 28 ,1)= 'COL28'.


compute colslab( 29 ,1)= 'COL29'.


compute colslab( 30 ,1)= 'COL30'.


compute colslab( 31 ,1)= 'COL31'.


compute colslab( 32 ,1)= 'COL32'.


compute colslab( 33 ,1)= 'COL33'.


compute colslab( 34 ,1)= 'COL34'.


compute colslab( 35 ,1)= 'COL35'.


compute colslab( 36 ,1)= 'COL36'.


compute colslab( 37 ,1)= 'COL37'.


compute colslab( 38 ,1)= 'COL38'.


compute colslab( 39 ,1)= 'COL39'.


compute colslab( 40 ,1)= 'COL40'.


compute colslab( 41 ,1)= 'COL41'.


compute colslab( 42 ,1)= 'COL42'.


compute colslab( 43 ,1)= 'COL43'.


compute colslab( 44 ,1)= 'COL44'.


compute colslab( 45 ,1)= 'COL45'.


compute colslab( 46 ,1)= 'COL46'.


compute colslab( 47 ,1)= 'COL47'.


compute colslab( 48 ,1)= 'COL48'.


compute colslab( 49 ,1)= 'COL49'.


compute colslab( 50 ,1)= 'COL50'.


compute colslab( 51 ,1)= 'COL51'.


compute colslab( 52 ,1)= 'COL52'.


compute colslab( 53 ,1)= 'COL53'.


compute colslab( 54 ,1)= 'COL54'.


compute colslab( 55 ,1)= 'COL55'.


compute colslab( 56 ,1)= 'COL56'.


compute colslab( 57 ,1)= 'COL57'.


compute colslab( 58 ,1)= 'COL58'.


compute colslab( 59 ,1)= 'COL59'.


compute colslab( 60 ,1)= 'COL60'.


compute colslab( 61 ,1)= 'COL61'.


compute colslab( 62 ,1)= 'COL62'.


compute colslab( 63 ,1)= 'COL63'.


compute colslab( 64 ,1)= 'COL64'.


compute colslab( 65 ,1)= 'COL65'.


compute colslab( 66 ,1)= 'COL66'.


compute colslab( 67 ,1)= 'COL67'.


compute colslab( 68 ,1)= 'COL68'.


compute colslab( 69 ,1)= 'COL69'.


compute colslab( 70 ,1)= 'COL70'.


compute colslab( 71 ,1)= 'COL71'.


compute colslab( 72 ,1)= 'COL72'.


compute colslab( 73 ,1)= 'COL73'.


compute colslab( 74 ,1)= 'COL74'.


compute colslab( 75 ,1)= 'COL75'.


compute colslab( 76 ,1)= 'COL76'.


compute colslab( 77 ,1)= 'COL77'.


compute colslab( 78 ,1)= 'COL78'.


compute colslab( 79 ,1)= 'COL79'.


compute colslab( 80 ,1)= 'COL80'.


compute colslab( 81 ,1)= 'COL81'.


compute colslab( 82 ,1)= 'COL82'.


compute colslab( 83 ,1)= 'COL83'.


compute colslab( 84 ,1)= 'COL84'.


compute colslab( 85 ,1)= 'COL85'.


compute colslab( 86 ,1)= 'COL86'.


compute colslab( 87 ,1)= 'COL87'.


compute colslab( 88 ,1)= 'COL88'.


compute colslab( 89 ,1)= 'COL89'.


compute colslab( 90 ,1)= 'COL90'.


compute colslab( 91 ,1)= 'COL91'.


compute colslab( 92 ,1)= 'COL92'.


compute colslab( 93 ,1)= 'COL93'.


compute colslab( 94 ,1)= 'COL94'.


compute colslab( 95 ,1)= 'COL95'.


compute colslab( 96 ,1)= 'COL96'.


compute colslab( 97 ,1)= 'COL97'.


compute colslab( 98 ,1)= 'COL98'.


compute colslab( 99 ,1)= 'COL99'.


compute colslab( 100 ,1)= 'COL100'.


compute colslab( 101 ,1)= 'COL101'.


compute colslab( 102 ,1)= 'COL102'.


compute colslab( 103 ,1)= 'COL103'.


compute colslab( 104 ,1)= 'COL104'.


compute colslab( 105 ,1)= 'COL105'.


compute colslab( 106 ,1)= 'COL106'.


compute colslab( 107 ,1)= 'COL107'.


compute colslab( 108 ,1)= 'COL108'.


compute colslab( 109 ,1)= 'COL109'.


compute colslab( 110 ,1)= 'COL110'.


compute colslab( 111 ,1)= 'COL111'.


compute colslab( 112 ,1)= 'COL112'.


compute colslab( 113 ,1)= 'COL113'.


compute colslab( 114 ,1)= 'COL114'.


compute colslab( 115 ,1)= 'COL115'.


compute colslab( 116 ,1)= 'COL116'.


compute colslab( 117 ,1)= 'COL117'.


compute colslab( 118 ,1)= 'COL118'.


compute colslab( 119 ,1)= 'COL119'.


compute colslab( 120 ,1)= 'COL120'.


compute colslab( 121 ,1)= 'COL121'.


compute colslab( 122 ,1)= 'COL122'.


compute colslab( 123 ,1)= 'COL123'.


compute colslab( 124 ,1)= 'COL124'.


compute colslab( 125 ,1)= 'COL125'.


compute colslab( 126 ,1)= 'COL126'.


compute colslab( 127 ,1)= 'COL127'.


compute colslab( 128 ,1)= 'COL128'.


compute colslab( 129 ,1)= 'COL129'.


compute colslab( 130 ,1)= 'COL130'.


compute colslab( 131 ,1)= 'COL131'.


compute colslab( 132 ,1)= 'COL132'.


compute colslab( 133 ,1)= 'COL133'.


compute colslab( 134 ,1)= 'COL134'.


compute colslab( 135 ,1)= 'COL135'.


compute colslab( 136 ,1)= 'COL136'.


compute colslab( 137 ,1)= 'COL137'.


compute colslab( 138 ,1)= 'COL138'.


compute colslab( 139 ,1)= 'COL139'.


compute colslab( 140 ,1)= 'COL140'.


compute colslab( 141 ,1)= 'COL141'.


compute colslab( 142 ,1)= 'COL142'.


compute colslab( 143 ,1)= 'COL143'.


compute colslab( 144 ,1)= 'COL144'.


compute colslab( 145 ,1)= 'COL145'.


compute colslab( 146 ,1)= 'COL146'.


compute colslab( 147 ,1)= 'COL147'.


compute colslab( 148 ,1)= 'COL148'.


compute colslab( 149 ,1)= 'COL149'.


compute colslab( 150 ,1)= 'COL150'.


compute colslab( 151 ,1)= 'COL151'.


compute colslab( 152 ,1)= 'COL152'.


compute colslab( 153 ,1)= 'COL153'.


compute colslab( 154 ,1)= 'COL154'.


compute colslab( 155 ,1)= 'COL155'.


compute colslab( 156 ,1)= 'COL156'.


compute colslab( 157 ,1)= 'COL157'.


compute colslab( 158 ,1)= 'COL158'.


compute colslab( 159 ,1)= 'COL159'.


compute colslab( 160 ,1)= 'COL160'.


compute colslab( 161 ,1)= 'COL161'.


compute colslab( 162 ,1)= 'COL162'.


compute colslab( 163 ,1)= 'COL163'.


compute colslab( 164 ,1)= 'COL164'.


compute colslab( 165 ,1)= 'COL165'.


compute colslab( 166 ,1)= 'COL166'.


compute colslab( 167 ,1)= 'COL167'.


compute colslab( 168 ,1)= 'COL168'.


compute colslab( 169 ,1)= 'COL169'.


compute colslab( 170 ,1)= 'COL170'.


compute colslab( 171 ,1)= 'COL171'.


compute colslab( 172 ,1)= 'COL172'.


compute colslab( 173 ,1)= 'COL173'.


compute colslab( 174 ,1)= 'COL174'.


compute colslab( 175 ,1)= 'COL175'.


compute colslab( 176 ,1)= 'COL176'.


compute colslab( 177 ,1)= 'COL177'.


compute colslab( 178 ,1)= 'COL178'.


compute colslab( 179 ,1)= 'COL179'.


compute colslab( 180 ,1)= 'COL180'.


compute colslab( 181 ,1)= 'COL181'.


compute colslab( 182 ,1)= 'COL182'.


compute colslab( 183 ,1)= 'COL183'.


compute colslab( 184 ,1)= 'COL184'.


compute colslab( 185 ,1)= 'COL185'.


compute colslab( 186 ,1)= 'COL186'.


compute colslab( 187 ,1)= 'COL187'.


compute colslab( 188 ,1)= 'COL188'.


compute colslab( 189 ,1)= 'COL189'.


compute colslab( 190 ,1)= 'COL190'.


compute colslab( 191 ,1)= 'COL191'.


compute colslab( 192 ,1)= 'COL192'.


compute colslab( 193 ,1)= 'COL193'.


compute colslab( 194 ,1)= 'COL194'.


compute colslab( 195 ,1)= 'COL195'.


compute colslab( 196 ,1)= 'COL196'.


compute colslab( 197 ,1)= 'COL197'.


compute colslab( 198 ,1)= 'COL198'.


compute colslab( 199 ,1)= 'COL199'.


compute colslab( 200 ,1)= 'COL200'.


compute colslab( 201 ,1)= 'COL201'.


compute colslab( 202 ,1)= 'COL202'.


compute colslab( 203 ,1)= 'COL203'.


compute colslab( 204 ,1)= 'COL204'.


compute colslab( 205 ,1)= 'COL205'.


compute colslab( 206 ,1)= 'COL206'.


compute colslab( 207 ,1)= 'COL207'.


compute colslab( 208 ,1)= 'COL208'.


compute colslab( 209 ,1)= 'COL209'.


compute colslab( 210 ,1)= 'COL210'.


compute colslab( 211 ,1)= 'COL211'.


compute colslab( 212 ,1)= 'COL212'.


compute colslab( 213 ,1)= 'COL213'.


compute colslab( 214 ,1)= 'COL214'.


compute colslab( 215 ,1)= 'COL215'.


compute colslab( 216 ,1)= 'COL216'.


compute colslab( 217 ,1)= 'COL217'.


compute colslab( 218 ,1)= 'COL218'.


compute colslab( 219 ,1)= 'COL219'.


compute colslab( 220 ,1)= 'COL220'.


compute colslab( 221 ,1)= 'COL221'.


compute colslab( 222 ,1)= 'COL222'.


compute colslab( 223 ,1)= 'COL223'.


compute colslab( 224 ,1)= 'COL224'.


compute colslab( 225 ,1)= 'COL225'.


compute colslab( 226 ,1)= 'COL226'.


compute colslab( 227 ,1)= 'COL227'.


compute colslab( 228 ,1)= 'COL228'.


compute colslab( 229 ,1)= 'COL229'.


compute colslab( 230 ,1)= 'COL230'.


compute colslab( 231 ,1)= 'COL231'.


compute colslab( 232 ,1)= 'COL232'.


compute colslab( 233 ,1)= 'COL233'.


compute colslab( 234 ,1)= 'COL234'.


compute colslab( 235 ,1)= 'COL235'.


compute colslab( 236 ,1)= 'COL236'.


compute colslab( 237 ,1)= 'COL237'.


compute colslab( 238 ,1)= 'COL238'.


compute colslab( 239 ,1)= 'COL239'.


compute colslab( 240 ,1)= 'COL240'.


compute colslab( 241 ,1)= 'COL241'.


compute colslab( 242 ,1)= 'COL242'.


compute colslab( 243 ,1)= 'COL243'.


compute colslab( 244 ,1)= 'COL244'.


compute colslab( 245 ,1)= 'COL245'.


compute colslab( 246 ,1)= 'COL246'.


compute colslab( 247 ,1)= 'COL247'.


compute colslab( 248 ,1)= 'COL248'.


compute colslab( 249 ,1)= 'COL249'.


compute colslab( 250 ,1)= 'COL250'.


compute colslab( 251 ,1)= 'COL251'.


compute colslab( 252 ,1)= 'COL252'.


compute colslab( 253 ,1)= 'COL253'.


compute colslab( 254 ,1)= 'COL254'.


compute colslab( 255 ,1)= 'COL255'.


compute colslab( 256 ,1)= 'COL256'.


compute colslab( 257 ,1)= 'COL257'.


compute colslab( 258 ,1)= 'COL258'.


compute colslab( 259 ,1)= 'COL259'.


compute colslab( 260 ,1)= 'COL260'.


compute colslab( 261 ,1)= 'COL261'.


compute colslab( 262 ,1)= 'COL262'.


compute colslab( 263 ,1)= 'COL263'.


compute colslab( 264 ,1)= 'COL264'.


compute colslab( 265 ,1)= 'COL265'.


compute colslab( 266 ,1)= 'COL266'.


compute colslab( 267 ,1)= 'COL267'.


compute colslab( 268 ,1)= 'COL268'.


compute colslab( 269 ,1)= 'COL269'.


compute colslab( 270 ,1)= 'COL270'.


compute colslab( 271 ,1)= 'COL271'.


compute colslab( 272 ,1)= 'COL272'.


compute colslab( 273 ,1)= 'COL273'.


compute colslab( 274 ,1)= 'COL274'.


compute colslab( 275 ,1)= 'COL275'.


compute colslab( 276 ,1)= 'COL276'.


compute colslab( 277 ,1)= 'COL277'.


compute colslab( 278 ,1)= 'COL278'.


compute colslab( 279 ,1)= 'COL279'.


compute colslab( 280 ,1)= 'COL280'.


compute colslab( 281 ,1)= 'COL281'.


compute colslab( 282 ,1)= 'COL282'.


compute colslab( 283 ,1)= 'COL283'.


compute colslab( 284 ,1)= 'COL284'.


compute colslab( 285 ,1)= 'COL285'.


compute colslab( 286 ,1)= 'COL286'.


compute colslab( 287 ,1)= 'COL287'.


compute colslab( 288 ,1)= 'COL288'.


compute colslab( 289 ,1)= 'COL289'.


compute colslab( 290 ,1)= 'COL290'.


compute colslab( 291 ,1)= 'COL291'.


compute colslab( 292 ,1)= 'COL292'.


compute colslab( 293 ,1)= 'COL293'.


compute colslab( 294 ,1)= 'COL294'.


compute colslab( 295 ,1)= 'COL295'.


compute colslab( 296 ,1)= 'COL296'.


compute colslab( 297 ,1)= 'COL297'.


compute colslab( 298 ,1)= 'COL298'.


compute colslab( 299 ,1)= 'COL299'.


compute colslab( 300 ,1)= 'COL300'.

compute colslab=colslab(1:ncol(bootres),1).
compute colslab={colslab,conseq,vlabs}.
print colslab/title='Map of column names to model coefficients:'/clabels=' ','Conseqnt','Antecdnt'/format=a8.
end if.
end if.
do if (criterr=0 and boot > 0 and modelbt=1).
compute labstart=1.
print/title = '*********** BOOTSTRAP RESULTS FOR REGRESSION MODEL PARAMETERS ************'.
loop iboot = 1 to (nms+nys).
print outnames(1,iboot)/title = 'OUTCOME VARIABLE:'/format = A8.
compute vlabsm=vlabs(labstart:(labstart+(nump(1,iboot)-1)),1).
print bootcim(labstart:(labstart+(nump(1,iboot)-1)),:)/title=' '/rnames=vlabsm/ clabels='Coeff' 'BootMean' 'BootSE' 'BootLLCI' 'BootULCI'/format= F10.4 /space=0.
compute labstart=labstart+nump(1,iboot).
do if (iboot < (nms+nys)).
print/title= '----------'.
end if.
end loop.
end if.
do if (criterr = 0 and matrices=1).
print/title = '************************ MODEL DEFINITION MATRICES ************************'.
print/title = 'FROM variables are columns, TO variables are rows.'.
compute temp2=make(nrow(bcmat),ncol(bcmat),'0').
loop i = 2 to nrow(bcmat).
loop j = 1 to (ncol(bcmat)-1).
do if (bcmat(i,j)=1).
compute temp2(i,j)='1'.
end if.
do if (j >= i).
compute temp2(i,j)=' '.
end if.
end loop.
end loop.
compute temp2=temp2(2:nrow(bcmat),(1:(ncol(bcmat)-1))).
do if (nms > 0).
compute cmatlabs={xnames,mnames}.
compute rmatlabs={mnames,ynames}.
end if.
do if (nms = 0).
compute cmatlabs={xnames}.
compute rmatlabs={ynames}.
end if.
print temp2/title='BMATRIX: Paths freely estimated (1) and fixed to zero (0):'/cnames=cmatlabs/rnames=rmatlabs/format A3.
compute z=0.
do if (rsum(csum(wcmat))<>0).
compute temp2=make(nrow(wcmat),ncol(wcmat),'0').
loop i = 2 to nrow(wcmat).
loop j = 1 to (ncol(wcmat)-1).
do if (wcmat(i,j)=1).
compute temp2(i,j)='1'.
end if.
do if (j >= i).
compute temp2(i,j)=' '.
end if.
end loop.
end loop.
compute temp2=temp2(2:nrow(wcmat),(1:(ncol(wcmat)-1))).
print temp2/title='WMATRIX: Paths moderated (1) and not moderated (0) by W:'/cnames=cmatlabs/rnames=rmatlabs/format A3.
end if.
do if (rsum(csum(zcmat))<>0).
compute temp2=make(nrow(zcmat),ncol(zcmat),'0').
loop i = 2 to nrow(zcmat).
loop j = 1 to (ncol(zcmat)-1).
do if (zcmat(i,j)=1).
compute temp2(i,j)='1'.
end if.
do if (j >= i).
compute temp2(i,j)=' '.
end if.
end loop.
end loop.
compute temp2=temp2(2:nrow(zcmat),(1:(ncol(zcmat)-1))).
print temp2/title='ZMATRIX: Paths moderated (1) and not moderated (0) by Z:'/cnames=cmatlabs/rnames=rmatlabs/format a3.
end if.
do if (rsum(csum(wzcmat))<>0).
compute temp2=make(nrow(wzcmat),ncol(wzcmat),'0').
loop i = 2 to nrow(wzcmat).
loop j = 1 to (ncol(wzcmat)-1).
do if (wzcmat(i,j)=1).
compute temp2(i,j)='1'.
end if.
do if (j >= i).
compute temp2(i,j)=' '.
end if.
end loop.
end loop.
compute temp2=temp2(2:nrow(wzcmat),(1:(ncol(wzcmat)-1))).
print temp2/title='WZMATRIX: W moderated paths moderated (1) and not moderated (0) by Z:'/cnames=cmatlabs/rnames=rmatlabs/format a3.
end if.
do if (ncs > 0).
print ccmat/title='CMATRIX: Covariates (columns) in (1) and not in (0) the models of M and Y (rows):'/rnames=rmatlabs/cnames=covnames.
end if.
end if.
print/title = '*********************** ANALYSIS NOTES AND ERRORS ************************'.
do if (criterr=0).
print conf/title = 'Level of confidence for all confidence intervals in output:'/format = F8.4.
do if (boot > 0).
do if (goodboot = boot).
print boot/title='Number of bootstrap samples for percentile bootstrap confidence intervals:'.
end if.
end if.
do if (mc > 0).
print mc/title='Number of samples for Monte Carlo confidence intervals:'.
end if.
do if (wnotev > 0 and printw=1).
do if (wnotev=1).
print/title = 'W values in conditional tables are the 16th, 50th, and 84th percentiles.'.
else if (wnotev=2).
do if (minwwarn=0 and maxwwarn=0).
print/title = 'W values in conditional tables are the mean and +/- SD from the mean.'.
end if.
do if (minwwarn=1).
print/title = 'W values in conditional tables are the minimum, the mean, and 1 SD above the mean.'.
end if.
do if (maxwwarn=1).
print/title = 'W values in conditional tables are 1 SD below the mean, the mean, and the maximum.'.
end if.
end if.
end if.
do if (znotev > 0 and printz=1).
do if (znotev=1).
print/title = 'Z values in conditional tables are the 16th, 50th, and 84th percentiles.'.
else if (znotev=2).
do if (minzwarn=0 and maxzwarn=0).
print/title = 'Z values in conditional tables are the mean and +/- SD from the mean.'.
end if.
do if (minzwarn=1).
print/title = 'Z values in conditional tables are the minimum, the mean, and 1 SD above the mean.'.
end if.
do if (maxzwarn=1).
print/title = 'Z values in conditional tables are 1 SD below the mean, the mean, and the maximum.'.
end if.
end if.
end if.
do if (minwwarn > 0).
print/title = 'NOTE: One SD below the mean is below the minimum observed in the data for W,'.
print/title = '      so the minimum measurement on W is used for conditioning instead.'/space=0.
end if.
do if (maxwwarn > 0).
print/title = 'NOTE: One SD above the mean is above the maximum observed in the data for W,'.
print/title = '      so the maximum measurement for W is used for conditioning instead.'/space=0.
end if.
do if (minzwarn > 0).
print/title = 'NOTE: One SD below the mean is below the minimum observed in the data for Z,'.
print/title = '      so the minimum measurement for Z is used for conditioning instead.'/space=0.
end if.
do if (maxzwarn > 0).
print/title = 'NOTE: One SD above the mean is above the maximum observed in the data for Z,'.
print/title = '      so the maximum measurement for Z is used for conditioning instead.'/space=0.
end if.
loop i = 1 to 100.
do if (notecode(i,1)=1).
print/title = 'NOTE: COVMY is ignored when using CMATRIX option.'.
end if.
do if (notecode(i,1)=2).
print/title = 'NOTE: Confidence level restricted to between 50 and 99.9999%.  95% confidence is provided in output'.
end if.
do if (notecode(i,1)=3).
print centvar/title = 'NOTE: The following variables were mean centered prior to analysis:'/format = a8.
end if.
do if (notecode(i,1) = 4).
print/title = 'NOTE: A heteroscedasticity consistent standard error and covariance matrix estimator was used.'.
end if.
do if (notecode(i,1) = 5).
print/title = 'NOTE: The HC3 option has been replaced with HC.  See the documentation.'.
end if.
do if (notecode(i,1) = 6).
print/title = 'NOTE: Due to estimation problems, some bootstrap samples had to be replaced.'.
print badboot/title='      The number of times this happened was:'/space=0/format=F8.0.
end if.
do if (notecode(i,1) = 7).
print/title = 'NOTE: The bootstrapping was not completed due to problematic bootstrap samples.'.
print/title = '      Bootstrap confidence intervals are therefore suppressed.'/space=0.
end if.
do if (notecode(i,1) = 8).
print/title = 'NOTE: The number of bootstrap samples was adjusted upward given your desired confidence.'.
end if.
do if (notecode(i,1) = 9).
print/title = 'NOTE: WMODVAL is ignored when W is specified as multicategorical.'.
end if.
do if (notecode(i,1) = 10).
print/title = 'NOTE: ZMODVAL is ignored when Z is specified as multicategorical.'.
end if.
do if (notecode(i,1) = 11).
print/title = 'NOTE: Total effect model and estimate generated only when all covariates are specified in all'.
print/title = '      models of M and Y.'/space=0.
end if.
do if (notecode(i,1) = 12).
print/title = 'NOTE: Total effect model and estimate generated only when X is freely estimated to affect each M'.
print/title = '      and both X and M are freely estimated to affect Y'/space=0.
end if.
do if (notecode(i,1) = 13).
print/title = 'NOTE: There are too many pairwise contrasts to conduct with this model.'.
end if.
do if (notecode(i,1) = 14).
print/title = 'NOTE: The number of contrast weights must equal the number of indirect effects.'.
end if.
do if (notecode(i,1) = 15).
print/title = 'NOTE: Monte Carlo confidence intervals not available for this model.'.
print/title = '      Bootstrapping is used instead.'/space=0.
end if.
do if (notecode(i,1) = 16).
print/title = 'NOTE: The number of Monte Carlo samples was adjusted upward given your desired confidence.'.
end if.
do if (notecode(i,1) = 19).
print/title = 'NOTE: Your contrast matrix is invalid or not applicable to this model.'.
end if.
do if (notecode(i,1) = 20).
print/title = 'NOTE: One of the groups specified by your contrast matrix does not exist in the data.'.
end if.
do if (notecode(i,1) = 21).
print/title = 'NOTE: The VARORDER option is not available in this release.'.
end if.
do if (notecode(i,1) = 22).
print/title = 'NOTE: The VMODVAL and QMODVAL options are not available in this release.'.
end if.
do if (notecode(i,1) = 23).
print/title = 'NOTE: The QUANTILE option is not available in this release.'.
end if.
end loop.
do if (toomany=1).
print/title='NOTE: Variables names longer than eight characters can produce incorrect output.'.
print/title='      Shorter variable names are recommended.'/space=0.
end if.
end if.
loop i = 1 to 100.
do if (errcode(i,1)=1).
print/title = 'ERROR: You must specify a Y and an X variable.'.
end if.
do if (errcode(i,1)=2).
print/title = 'ERROR: A variable can appear only once in a PROCESS command.'.
do if (toomany = 1).
print/title = '       This might be caused by the use of variables names longer'/space=0.
print varnames/title = '       than eight characters. Here are the variables I see:'/space=0/format=A8.
end if.
end if.
do if (errcode(i,1)=3).
print/title = 'ERROR: You have specified more than one variable for W, Y, M, or Z'.
end if.
do if (errcode(i,1)=4).
print/title = 'ERROR: A variable specified as multicategorical has more than nine categories.'.
end if.
do if (errcode(i,1)=5).
print/title = 'ERROR: One of the categories contains only a single case.'.
end if.
do if (errcode(i,1)=6).
print/title = 'ERROR: Invalid model number in this version of PROCESS.'.
end if.
do if (errcode(i,1)=7).
print/title = 'ERROR: Invalid model number.'.
end if.
do if (errcode(i,1)=8).
print/title = 'ERROR: You must specify an M variable for this model.'.
end if.
do if (errcode(i,1)=9).
print/title = 'ERROR: You have specified an M variable in a model that does not use it.'.
print/title = 'In this release of PROCESS, moderators are W and Z in models 1, 2, and 3.'/space=0.
end if.
do if (errcode(i,1)=10).
print/title = 'ERROR: You have specified a W variable in a model that does not use it.'.
end if.
do if (errcode(i,1)=11).
print/title = 'ERROR: You have not specified a W variable in a model that requires it.'.
end if.
do if (errcode(i,1)=12).
print/title = 'ERROR: You have specified a Z variable in a model that does not use it.'.
end if.
do if (errcode(i,1)=13).
print/title = 'ERROR: You have not specified a Z variable in a model that requires it.'.
end if.
do if (errcode(i,1)=14).
print/title = 'ERROR: V and Q are not proper specifications in this release of PROCESS.'.
print/title = '       Moderators must be specified as W and/or Z.'/space=0.
end if.
do if (errcode(i,1)=15).
print/title = 'ERROR: One of your model variables exhibits no variation (it is a constant).'.
end if.
do if (errcode(i,1)=16).
print/title = 'ERROR: BMATRIX is not the correct length or is otherwise invalid.'.
end if.
do if (errcode(i,1)=17).
print/title = 'ERROR: WMATRIX is not the correct length or is otherwise invalid.'.
end if.
do if (errcode(i,1)=18).
print/title = 'ERROR: ZMATRIX is not the correct length or is otherwise invalid.'.
end if.
do if (errcode(i,1)=19).
print/title = 'ERROR: WZMATRIX is not the correct length or is otherwise invalid.'.
end if.
do if (errcode(i,1)=20).
print/title = 'ERROR: A path fixed at zero cannot be moderated.'.
end if.
do if (errcode(i,1)=21).
print/title = 'ERROR: If only one moderator is specified, it must be specified as W.'.
end if.
do if (errcode(i,1)=22).
print/title = 'ERROR: In BMATRIX, X must be specified to affect at least one variable.'.
end if.
do if (errcode(i,1)=23).
print/title = 'ERROR: In BMATRIX, at least one variable must be specified to affect Y.'.
end if.
do if (errcode(i,1)=24).
print/title = 'ERROR: You must specify a model number or a custom BMATRIX specification.'.
end if.
do if (errcode(i,1)=25).
print/title = 'ERROR: BMATRIX cannot be used in conjunction with a model number.'.
end if.
do if (errcode(i,1)=26).
print/title = 'ERROR: Your model has a dangling mediator (all Ms must affect and be affected).'.
end if.
do if (errcode(i,1)=27).
print/title = 'ERROR: CLUSTER is not available on this release of PROCESS.'.
end if.
do if (errcode(i,1)=29).
print/title = 'ERROR: CMATRIX is not the correct length or is otherwise invalid.'.
end if.
do if (errcode(i,1)=30).
print/title = 'ERROR: In CMATRIX, all covariates must be assigned to an M or a Y.'.
end if.
do if (errcode(i,1)=31).
print/title = 'ERROR: A linear or near linear dependency (singularity) exists in the data.'.
end if.
do if (errcode(i,1)=32).
print/title = 'ERROR: Models 80 and 81 require between 3 and 6 mediators.'.
end if.
do if (errcode(i,1)=33).
print/title = 'ERROR: Model 82 requires 4 mediators.'.
end if.
do if (errcode(i,1)=34).
print/title = 'ERROR: This model number requires between 2 and 6 mediators.'.
end if.
do if (errcode(i,1)=35).
print/title = 'ERROR: In a model with only one moderator, that moderator must be W.'.
end if.
do if (errcode(i,1)=36).
print/title = 'ERROR: A serial mediation model cannot have more than 6 mediators.'.
end if.
do if (errcode(i,1)=37).
print/title = 'ERROR: No more than 10 mediators are allowed in a PROCESS command.'.
end if.
do if (errcode(i,1)=38).
print/title = 'ERROR: XCATCODE is not provided, not the correct length, or is otherwise invalid.'.
end if.
do if (errcode(i,1)=39).
print/title = 'ERROR: WCATCODE is not provided, not the correct length, or is otherwise invalid.'.
end if.
do if (errcode(i,1)=40).
print/title = 'ERROR: ZCATCODE is not provided, not the correct length, or is otherwise invalid.'.
end if.
do if (errcode(i,1)=41).
print/title = 'ERROR: Models 1, 2, 3, and 74 cannot be customized.'.
end if.
do if (errcode(i,1)=42).
print/title = 'ERROR: WS option available only in PROCESS v2. Or use the MEMORE macro instead.'.
print/title = '       MEMORE can be downloaded from www.akmontoya.com.'/space=0.
end if.
do if (errcode(i,1)=43).
print/title = 'ERROR: PROCESS does not allow dichotomous mediators.'.
end if.
do if (errcode(i,1)=44).
print/title = 'ERROR: This release of PROCESS does not allow a dichotomous Y.'.
end if.
do if (errcode(i,1)=45).
print/title = 'ERROR: In model 74, X and W must be the same variable.'.
end if.
do if (errcode(i,1)=46).
print/title = 'ERROR: Model 74 is temporarily disabled in this release of PROCESS.'.
end if.
end loop.
END MATRIX.	
资源	处理器时间	00:00:03.23	
	已用时间	00:00:03.25	


[数据集1] F:\准备投稿的文章\亲社会动画与攻击行为：动机中介-辣酱范式168\投搞材料\男生.sav


Run MATRIX procedure:

**************** PROCESS Procedure for SPSS Version 3.00 *****************

          Written by Andrew F. Hayes, Ph.D.       www.afhayes.com
    Documentation available in Hayes (2018). www.guilford.com/p/hayes3

**************************************************************************
Model  : 4
    Y  : Z后AB
    X  : Zcartoon
    M  : Zmotivat

Covariates:
 age

Sample
Size:  84

**************************************************************************
OUTCOME VARIABLE:
 Zmotivat

Model Summary
          R       R-sq        MSE          F        df1        df2          p
      .4786      .2291      .7864    12.0343     2.0000    81.0000      .0000

Model
              coeff         se          t          p       LLCI       ULCI
constant      .0706     1.2668      .0557      .9557    -2.4500     2.5912
Zcartoon     -.4783      .0976    -4.9007      .0000     -.6725     -.2841
age          -.0286      .2144     -.1336      .8941     -.4553      .3980

**************************************************************************
OUTCOME VARIABLE:
 Z后AB

Model Summary
          R       R-sq        MSE          F        df1        df2          p
      .6361      .4046      .7666    18.1196     3.0000    80.0000      .0000

Model
              coeff         se          t          p       LLCI       ULCI
constant      .5054     1.2508      .4041      .6872    -1.9838     2.9946
Zcartoon     -.4364      .1097    -3.9774      .0002     -.6548     -.2181
Zmotivat      .3894      .1097     3.5499      .0006      .1711      .6077
age          -.0784      .2118     -.3702      .7122     -.4998      .3430

****************** DIRECT AND INDIRECT EFFECTS OF X ON Y *****************

Direct effect of X on Y
     Effect         se          t          p       LLCI       ULCI
     -.4364      .1097    -3.9774      .0002     -.6548     -.2181

Indirect effect(s) of X on Y:
             Effect     BootSE   BootLLCI   BootULCI
Zmotivat     -.1863      .0730     -.3517     -.0701

*********************** ANALYSIS NOTES AND ERRORS ************************

Level of confidence for all confidence intervals in output:
  95.0000

Number of bootstrap samples for percentile bootstrap confidence intervals:
  5000

NOTE: Variables names longer than eight characters can produce incorrect output.
      Shorter variable names are recommended.

------ END MATRIX -----
